# Elucidating Formation Mechanisms of Inorganic Sulfates upon OH Radical Oxidation of Organosulfates via Non-Sulfur Radical Pathways: Insights from Methyl Sulfate

**DOI:** 10.1021/jacs.6c01898

**Published:** 2026-06-24

**Authors:** Tianren Zhu, Sheung Ki Suki Chan, Lena Gerritz, Sergey A. Nizkorodov, Manabu Shiraiwa, Man Nin Chan, Ying-Lung Steve Tse

**Affiliations:** † Department of Chemistry, 26451 The Chinese University of Hong Kong, Sha Tin, Hong Kong, China; ‡ Department of Earth and Environmental Sciences, The Chinese University of Hong Kong, Sha Tin, Hong Kong, China; § Department of Chemistry, 8788 University of California, Irvine, Irvine, California 92697, United States

## Abstract

Organosulfates are major reservoirs of sulfur in atmospheric aerosols, yet the molecular steps by which hydroxyl radicals (OH) convert organosulfates into inorganic sulfate in aqueous particles remain poorly constrained. This uncertainty limits the mechanistic interpretation of organosulfate chemical aging and its representation in atmospheric models. Here, we use methyl sulfate, CH_3_OSO_3_^–^, the simplest organosulfate, as a model system to elucidate OH-initiated aqueous oxidation chemistry through integrated quantum-chemical calculations, *in situ* electron paramagnetic resonance spin trapping, and high-resolution mass spectrometry. Calculations show that OH oxidation is initiated predominantly by methyl H-abstraction rather than direct attack at the sulfate ester, producing a carbon-centered radical that rapidly adds O_2_ to form a peroxy radical. Subsequent peroxy–peroxy chemistry forms a tetroxide intermediate whose decomposition favors stepwise O–O bond scission to alkoxy sulfate radicals, while classical Russell and Bennett–Summers pathways are kinetically inaccessible. The key sulfate-forming step is a water-assisted, proton-coupled C–O bond cleavage of the alkoxy sulfate radical. Extended hydrogen-bond networks markedly lower the barrier for this fragmentation, enabling direct formation of bisulfate/sulfate and a formyl radical without requiring sulfite or sulfate radical anions as obligatory intermediates. Experimentally, mass spectrometry detects a BMPO-trapped methyl sulfate radical and an adduct consistent with formyl-radical formation, whereas sulfur-centered radical adducts are minor under our conditions. These combined results reveal a non-sulfur radical pathway for inorganic sulfate formation from organosulfates and highlight the central role of explicit solvation and proton transfer in aqueous aerosol sulfur chemistry.

## Graphical abstract

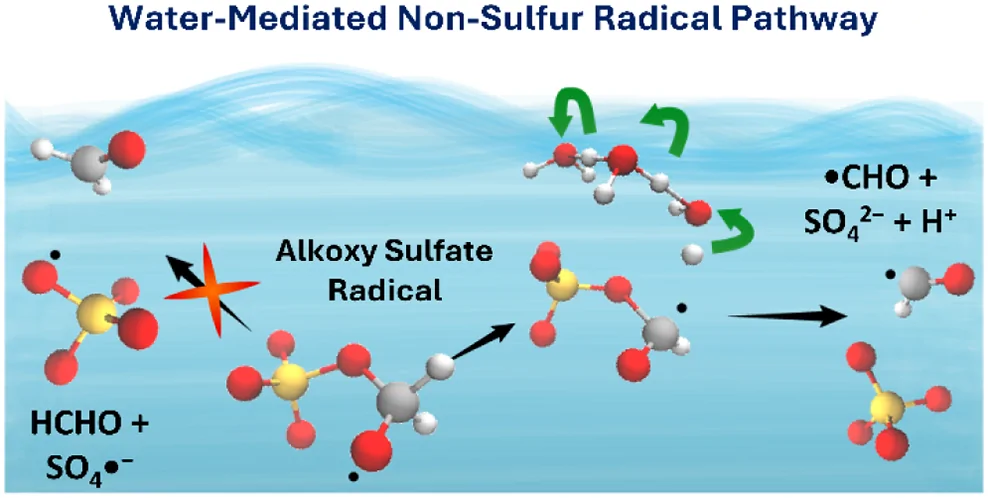

## 1 Introduction

There is increasing evidence that sulfur in atmospheric aerosols can exist in the form of organosulfur compounds.^1^ By measuring the difference between total sulfur and inorganic sulfur using X-ray fluorescence and ion chromatography, it has been determined that organosulfur compounds can account for up to 30% of the total organic aerosol mass at a forest site in K-puszta, Hungary.^2^ A similar approach applied to the National Park Service IMPROVE PM2.5 database by Tolocka and Turpin revealed that organosulfur compounds contribute 5–10% to the total organic aerosol mass across various regions in the United States.^3,4^ Among the organosulfur compounds, sulfuric acid esters (ROSO_3_H), known in the atmospheric chemistry community as “organosulfates” (OSs), have been found to contribute a significant portion of the organic mass of atmospheric aerosols.^5–12^

The formation mechanisms of OSs have been extensively studied and are summarized elsewhere.^13,14^ Once formed, OSs can undergo continuous transformation in the atmosphere. Certain OSs, particularly tertiary OSs, tend to hydrolyze, producing polyols and sulfuric acid on time scales ranging from minutes to tens of hours under typical atmospheric conditions.^15–17^ Recently, efficient oxidation of OSs by gas-phase OH radicals near the aerosol surface has been reported.^18–24^ In these studies, OH oxidation of OSs produces not only new organic species but also inorganic sulfur species, such as bisulfate (HSO_4_^–^) and sulfate (SO_4_^2–^) ions. This process provides an avenue for the interconversion of aerosol sulfur between its organic and inorganic forms.^25^ Given the distinct physicochemical properties of sulfur in its organic and inorganic forms, quantifying the partitioning between organic and inorganic sulfur is important because these sulfur pools are represented differently in atmospheric models and can influence different aerosol properties and chemical processes.^26–28^ Inorganic sulfate is closely linked to aerosol acidity, inorganic ion balance, and water uptake, whereas organosulfates contribute to organic aerosol mass and can differ in hygroscopicity, surface activity, and chemical lifetime. Therefore, oxidation-driven conversion of organosulfates to inorganic sulfate/bisulfate can alter aerosol composition, acidity, water content, and multiphase reactivity during atmospheric processing. We do not imply that organic–inorganic sulfur partitioning is necessarily the dominant uncertainty in predictions of aerosol optical or cloud properties; rather, mechanistic constraints on this conversion are needed to improve representation of atmospheric sulfur cycling and aerosol chemical evolution.

Arising from anthropogenic emissions of alkanes or detergents, alkyl OSs are prevalent in urban and industrial atmospheres.^25^ While the formation of inorganic sulfur species from OH oxidation has been observed, the detailed transformation pathways, particularly for alkyl organosulfates, remain unclear. To bridge this gap of knowledge, we study in this paper the oxidation pathways of methyl sulfate anion (CH_3_OSO_3_^–^), the smallest alkyl organosulfate identified in atmospheric aerosol particles.^29^

In the OH oxidation of methyl sulfate anion, fragmentation was previously proposed as the predominant process, as illustrated in Scheme 1.^18^ The absence of functionalization products has been ascribed to the higher energy barriers encountered during rearrangement of the tetroxide intermediate (ROOOOR) into appropriate configurations necessary for Bennett–Summers mechanisms and Russell reactions. This hindrance could be attributed to the bulky sulfate ester (−OSO_3_^–^) group connected to −CH_2_O_2_•. The oxidation primarily results in the formation and bond cleavage of the −CH_2_O• radical, formed after the tetroxide decomposition, through two pathways: (1) rearrangement of a proton from the radical carbon atom to the methoxy group, leading to decomposition into a formyl radical (•CHO) and HSO_4_^–^, or (2) decomposition into formaldehyde (HCHO) and sulfate radical (SO_4_•^–^). The •CHO promptly converts into carbon monoxide (CO), which returns to the gas phase.^18^ Meanwhile, sulfur-containing fragments and radicals (i.e., SO_4_•^–^) tend to reside in the particle phase due to their low volatility, subsequently transforming into inorganic sulfates (e.g., HSO_4_^–^ and SO_4_^2–^) through a sequence of particle-phase reactions.^30–34^

**Scheme 1:**
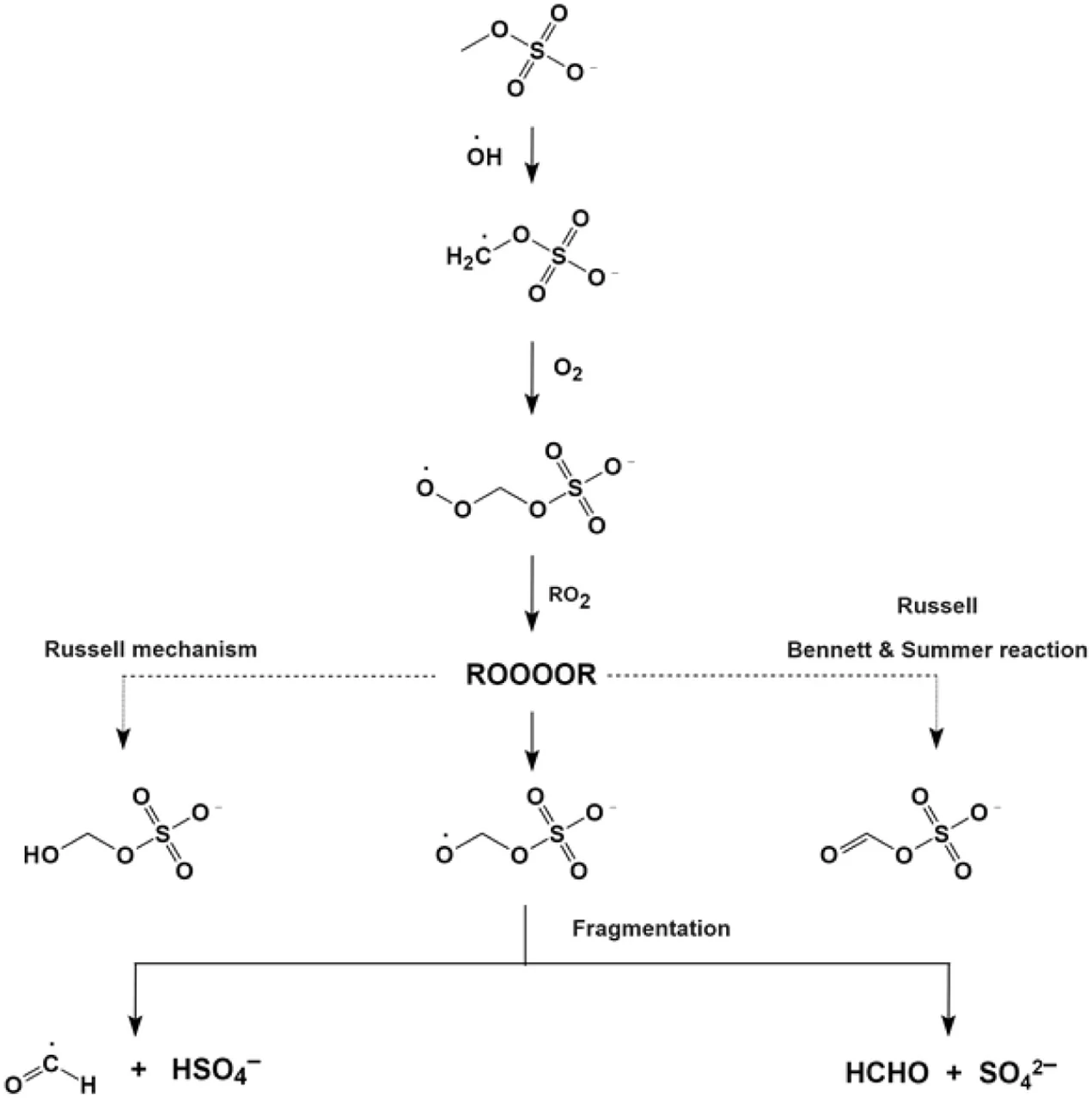
Previously Proposed Reaction Mechanisms for Heterogeneous OH Oxidation of Sodium Methyl Sulfate (Gray Arrows Denote Minor Pathways)^18^

In this work, the structural simplicity of methyl sulfate anion (CH_3_OSO_3_^–^) enables a more comprehensive exploration of the possible chemical transformation pathways of OSs by quantum mechanical (QM) calculations complemented by electron paramagnetic resonance (EPR) spectroscopy and mass spectrometry (MS) experiments. Beyond simply identifying intermediates, we aim to elucidate the mechanism of inorganic sulfate formation from OH-initiated oxidation of methyl sulfate, with particular attention to the relative roles of sulfur radical and conventional non-sulfur radical pathways. The calculations show that OH-initiated chemistry is funneled through hydrogen abstraction, O_2_ addition, RO_2_ self-reaction, and tetroxide decomposition that favors stepwise O–O bond scission over Russell and Bennett–Summers termination. The resulting alkoxy sulfate radical undergoes a *water-mediated, proton-coupled* C–O cleavage enabled by an extended hydrogen-bond network, directly yielding HSO_4_^–^/SO_4_^2–^ together with a formyl radical. This distinguishes the chemistry of organosulfates from that of typical aqueous sulfur oxidation chemistry like S­(IV) autoxidation^30,34,35^ and sulfate-radical–based advanced oxidation processes,^31,32,36^ where sulfur-centered radicals (SO_3_•^–^, SO_5_•^–^, and/or SO_4_•^–^) are the canonical intermediates. Experimentally, we observe BMPO (5-tert-butoxycarbonyl-5-methyl-1-pyrroline-N-oxide) adduct chemistry consistent with formyl-radical formation, while sulfate/sulfite radical adducts are minor under our conditions. Together, these results provide a mechanistic basis for rapid organosulfate to inorganic sulfate conversion in the aqueous phase and emphasize the controlling roles of solvation and proton transfer in organosulfate oxidation. These findings highlight the importance of non-sulfur radical pathways in driving the interconversion of aerosol sulfur and provide new insights into dynamic partitioning between organic and inorganic sulfur in the atmosphere.

## 2 Computational Details

### 2.1 Hybrid Solvation Model

As supported by our previous work, OH oxidation in aerosols is more likely to proceed after OH is absorbed into the aqueous phase from the interface, followed by subsequent diffusion and reaction with OS species inside the aerosol rather than via direct collisions with surface OS species.^24,37^ This scenario is particularly relevant to methyl sulfate, which, owing to its high water solubility and short alkyl chain, is expected to partition into the bulk water phase of droplets.^28^ Therefore, to better understand OH oxidation of methyl sulfate ion, we adopted hybrid solvation models to study the reaction in the aqueous phase.

The methyl sulfate ion (CH_3_OSO_3_^–^) carries a negative charge and forms strong hydrogen bonds with surrounding water molecules, leading to a compact and highly hydrated solvation shell. Moreover, its oxidation may involve sulfate/sulfite radicals (SO_4_•^–^/SO_3_•^–^), which are known to be potent oxidants in the aqueous phase.^32^ These characteristics suggest that water molecules play an active role in the reaction mechanism, particularly in pathways involving proton transfer steps. To account for possible interactions with water, we employed a hybrid solvation model that combined 6 explicit water molecules with the implicit SMD water model.^38^ The number of explicit water molecules was chosen based on both experimental and theoretical considerations. X-ray scattering and molecular dynamics simulations indicate that the coordination number of sulfate ions (SO_4_^2–^) in water typically ranges from 8 to 14, with an average around 11.^39,40^ Additionally, studies on SO_4_^2–^ (H_2_O)_n_ clusters at 21 °C (for *n* = 6–17) show that water loss dominates the dissociation pathway, while charge separation becomes significant for *n* < 4.^41^ Considering these findings and the presence of methyl group in our system, we selected 6 explicit water molecules as a reasonable compromise between accuracy and computational cost.

### 2.2 DFT Computational Details

Within this aqueous-phase framework, we further considered the possible singlet/triplet biradical intermediates in the presence of water (Scheme 2 shows the overall pathway, more details such as the spin states are presented in subsequent figures). We followed the approach by Bach, Dmitrenko and Estévez, who employed broken-symmetry unrestricted DFT to locate metastable singlet caged biradicals, including the O–O bond homolysis transition state, with computed energies comparable to the more accurate but expensive multi-reference CASSCF method.^42,43^ The initial conformational search was carried out using Molclus^44^ to select the most thermodynamically favorable conformer prior to locating the transition state. Geometry optimizations for all reactants, pre-reactive complexes, transition states, and products were carried out using Gaussian 16 C.02 ^45^ at the calculation level of M06-2X hybrid density functional^46^ with D3(0) dispersion corrections^47^ and the def2-TZVP basis set.^48^ Frequency analysis was performed at the same level to confirm the nature of stationary points. The stable reactants, intermediates, and products exhibited no imaginary frequencies, while transition states were validated by intrinsic reaction coordinate (IRC)^49^ calculations to ensure proper connectivity between reactants and products. Single-point energy calculations were performed at the M06-2X-D3(0)/ma-def2-QZVPP^48,50^ level without the implicit SMD water model. To account for free energy corrections due to solvation effects, we evaluated the energy differences between gas-phase and solvated environments using the M05-2X functional^51^ with the 6-31G*^52^ basis set, following the methodology established in the original SMD framework and the study by Coote *et al*.^53^ The final Gibbs free energy *G* was computed in Shermo^54^ as:

$$G=E_{elec}+\Delta G_{corr}+\Delta G_{solv}$$

where *E*_elec_ is the gas-phase single-point energy, Δ*G*_solv_ is the solvation free energy, and Δ*G*_corr_ was obtained from frequency analysis using the quasi-rigid rotor-harmonic oscillator (quasi-RRHO)^55^ approximation, including zero-point vibrational energy with rigid rotor-harmonic oscillator approximation and finite-temperature corrections at 298 K and 1 bar. The computed structures were visualized using CYLview^56^ and VMD v1.9.3.^57^ All the free energy profiles were plotted using the Energy Diagram Plotter (CDXML) developed by Li.^58^ Rate constants were also calculated for all relevant reactions. Details are provided in the “Oxidation Kinetics: Rate Constants for the Kinetic Model at 298 K” in the Supporting Information (SI) for details.

**Scheme 2:**
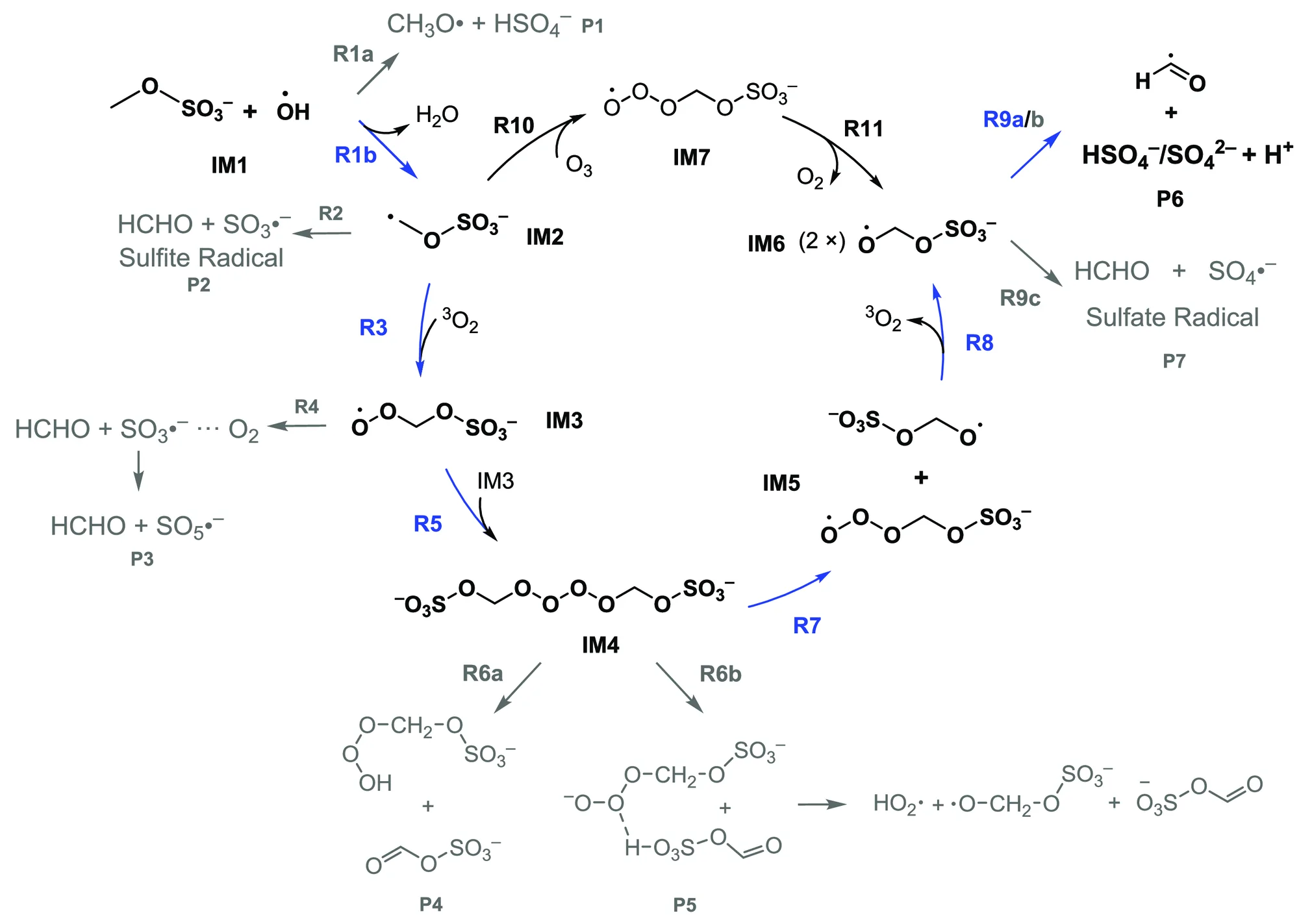
Proposed Reaction Mechanism for Aqueous OH Oxidation of Methyl Sulfate^a^

a Blue arrows indicate dominant reaction pathways, while gray arrows denote kinetically or thermodynamically unfavorable minor paths and products.

## 3 Experimental Methods

To identify both the radical intermediates and final products formed during the reaction, we conducted aqueous-phase OH oxidation experiments with sodium methyl sulfate. Throughout the reactions, newly generated radicals were trapped using BMPO and analyzed *in situ* via EPR spectroscopy. In addition, the reaction solutions were analyzed by electrospray ionization mass spectrometry (*m/z* 50–750, negative/positive ion mode) to detect potential sulfate species or trapped organic radical species formed during the reactions. The parameters and methods for the mass spectrometry analysis were the same as previous work.^59^ Please refer to the SI for electron paramagnetic resonance spectroscopy details.

## 4 Results and Discussion

Referring to Scheme 2, the oxidation of the methyl sulfate anion (CH_3_OSO_3_^–^) by OH can proceed via at least two possible initial pathways: (1) a direct attack by OH on the sulfate ester group, leading to the formation of HSO_4_^–^ via **R1a**, or (2) hydrogen abstraction from the methyl group by OH, forming •CH_2_OSO_3_^–^ (**IM2**) via **R1b**. From **IM2**, three distinct pathways can be identified. The first route involves unimolecular fragmentation to produce SO_3_•^–^ and formaldehyde (HCHO) via **R2**. The second, involves rapid reaction with molecular oxygen (O_2_) to form a •O_2_CH_2_OSO_3_^–^ (**IM3**) via **R3**. As recently reported by Tchinda *et al.*,^60^ a third branch proceeds via ozone (O_3_), yielding the oxidized intermediate (**IM7**) via **R10**, which subsequently reacts to form •OCH_2_OSO_3_^–^ (**IM6**) and O_2_ via **R11**. Despite their distinct origins, both the O_2_-initiated path (**R3** → **R5** → **R7** → **R8**) and the O_3_-initiated path (**R10** → **R11**) eventually converge at **IM6**, a key branching node toward inorganic sulfate products.

We further hypothesize that **IM3** can either fragment to generate SO_3_•^–^ via **R4** or participate in a self-reaction with **IM3** via **R5** (more details later in Figure 3a and related discussion), producing a disulfate ion intermediate **IM4**. Tetroxide **IM4** may undergo further transformation via three possible pathways: (1) formation of a carbonyl-functionalized product via the Bennett and Summers mechanism (shown in Figure 4 later),^61^ (2) generation of carbonyl products through Russell-type reactions^62^ via **R6** (more details in Figure 5 later), or (3) consecutive single-bond cleavages leading to the formation of **IM6** via **R5** → **R7** → **R8** (see also Figure 6 later). In parallel, **IM6** can also be formed via the O_3_-initiated sequence described above. Once formed, **IM6** can follow two competing routes: (1) fragmentation to yield SO_4_•^–^ via **R9b**, or (2) decomposition of **IM6**, involving proton transfer to the sulfate ester group and resulting in the formation of HSO_4_^–^ via **R9a**. This proton transfer can occur either intramolecularly through a five-membered cyclic transition state or be mediated by water molecules, as discussed in detail later.

To evaluate the chemical kinetics and spontaneity for forming the inorganic sulfate species through the OH-initiated oxidation of methyl sulfate ion, we computed the free energy barriers ΔG^‡^ and Gibbs free energy changes ΔG for each proposed reaction pathway. These results are summarized in Table 1, with further computational details provided in the Supporting Information (SI). As shown in Scheme 2 and with thermodynamic data summarized in Table 1 and Table S1, five key pathways (with each final step being **R1a, R2, R4, R9a**, and **R9c**, respectively) contribute to the formation of three distinct inorganic sulfur species: HSO_4_^–^, SO_3_•^–^, and SO_4_•^–^. DFT results indicate the most probable route for inorganic sulfate formation proceeds via the sequence **R1b → R3 → R5 → R7 → R8 → R9a**, ultimately yielding HSO_4_^–^ and •CHO as the predominant products via non-sulfur radical pathways. This conclusion is further supported by our experimental observations from EPR spectroscopy and MS, particularly the detection of BMPO adducts, BMPO-CHO or BMPO-CH_3_O, consistent with •CHO formation. Its presence aligns with our calculations and confirms the viability of this pathway (more details later). Also, the alternative O_3_-initiated path **R1b → R10 → R11 → R9a** provides a converging route to **IM6**, ultimately forming HSO_4_^–^. These results underscore the chemical diversity of pathways leading to the same inorganic sulfate product, reflecting the intrinsic flexibility of the oxidation network.

**Table 1:** Highest Free Energy Barriers Δ*G* ^‡^ (kJ/mol) and Free Energy Changes Δ*G* (kJ/mol) for the Major Proposed Pathways of OH Reaction with Methyl Sulfate Anion at 298 K and 1 bar^a^

Label	Reaction	Δ*G* ^‡^	Δ*G*
R1a	${\mathrm{RH}+\mathrm{OH}\to {\;\mathrm{CH}}_{3}{\mathrm{O•}+\;\mathrm{HSO}}_{4}}^{‐}$	161.7	–50.8
R1b	$\mathrm{RH}+\mathrm{OH}\to \;\mathrm{R•}+\;H_{2}O$	56.7	–87.9
R2	$\mathrm{R•}\to \;\mathrm{HCHO}+\;{\mathrm{SO}}_{3}{•}^{‐}$	76.7	5.1
R3	$\mathrm{R•}+\;O_{2}\to \;\mathrm{ROO•}$	11.5	–137.6
R5	2ROO•→ROOOOR	48.9	–25.6
R7	ROOOOR→RO•+ROOO•	70.1	39.6
R8	$\mathrm{RO}•+\mathrm{ROOO}•\to 2\mathrm{RO}•+O_{2}$	4.2	–63.1
R9a	${\mathrm{RO}•\to •\mathrm{CHO}+{\mathrm{HSO}}_{4}}^{‐}$	27.2	–38.8
R9a–22w	$\mathrm{RO}•\to •\mathrm{CHO}+{\mathrm{SO}}_{4}^{2‐}+H^{+}$	8.1	–49.6

a R = −CH_2_OSO_3_^–^ in the chemical formulas. For a list of alternative and minor pathways depicted in Scheme 2, including their corresponding thermodynamic data, please see Table S1 in the SI.

### 4.1 Initial Oxidation and Peroxy Radical Formation

The oxidative processing of the methyl sulfate anion is initiated by the OH. As illustrated in Figure 1, this interaction presents a kinetic bifurcation between nucleophilic substitution at the sulfur center (**R1a**) and hydrogen abstraction from the methyl group (**R1b**). Our calculations identify hydrogen abstraction as the exclusive pathway.

R1a $${{\mathrm{CH}}_{3}{{\mathrm{OSO}}_{3}}^{-}+•\mathrm{OH}\to •{\mathrm{OCH}}_{3}+{\mathrm{HSO}}_{4}}^{‐}$$

R1b $${\mathrm{CH}}_{3}{{\mathrm{OSO}}_{3}}^{-}+•\mathrm{OH}\to •{\mathrm{CH}}_{2}{{\mathrm{OSO}}_{3}}^{-}+H_{2}O$$

**Figure 1:**
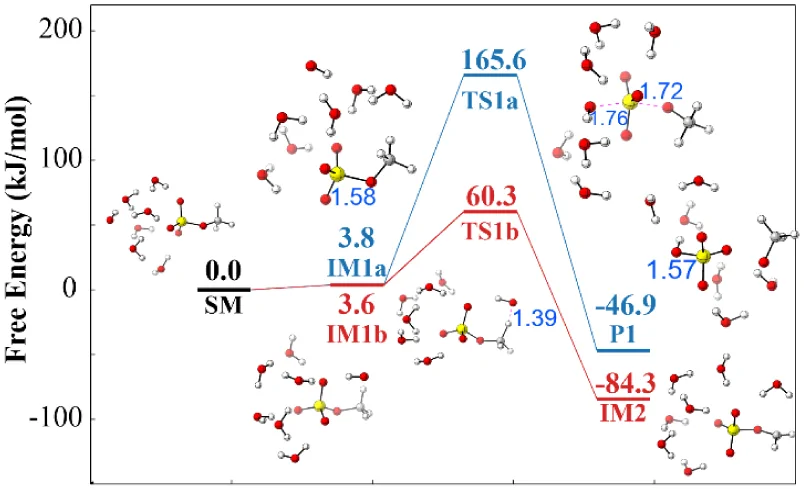
Free energy profile of the initial reactions of OH and methyl sulfate. IM1a and IM1b are the intermediate complexes for R1a and R1b preceding to their corresponding transition states. Sulfur, oxygen, carbon, and hydrogen atoms are shown as yellow, red, gray, and white, respectively.

The nucleophilic attack (**R1a**) is impeded by a prohibitive activation barrier (Δ*G* ^‡^ = +161.7 kJ/mol), which we attribute to the severe electrostatic repulsion between the incoming OH radical and the anionic sulfate group. Spin population analysis of **TS-R1a** confirms the high-energy nature of this process, showing a rupture of the S–OCH_3_ bond where spin density is forced to localize on the departing methoxy fragment (see Figure S3). Conversely, hydrogen abstraction (**R1b**) proceeds via a significantly lower barrier (Δ*G* ^‡^ = +56.7 kJ/mol) and is thermodynamically driven (Δ*G* = −87.9 kJ/mol). In **TS-R1b**, the spin density migrates smoothly from the OH radical to the α-carbon, yielding the methyl sulfate radical (**IM2**, •CH_2_OSO_3_^–^). This kinetic preference aligns with the established reactivity of saturated hydrocarbons and is experimentally corroborated by our MS result of the BMPO-trapped methyl sulfate radical (m/*z* 308.081), confirming **IM2** as the primary radical intermediate. As a result, we expect **R1b** to be the more probable step. This aligns with prior findings in the literature, as hydrogen abstraction is a dominant reaction pathway for OH attack on saturated hydrocarbons.^18,63–65^

R2 $${•{\mathrm{CH}}_{2}{{\mathrm{OSO}}_{3}}^{-}\to \mathrm{HCHO}+{\mathrm{SO}}_{3}•}^{-}$$

R3 $$•{\mathrm{CH}}_{2}{{\mathrm{OSO}}_{3}}^{-}+O_{2}\to •{\mathrm{OOCH}}_{2}{{\mathrm{OSO}}_{3}}^{-}$$

R10 $$•{\mathrm{CH}}_{2}{{\mathrm{OSO}}_{3}}^{-}+O_{3}\to •{\mathrm{OOOCH}}_{2}{{\mathrm{OSO}}_{3}}^{-}$$

R11 $$•{\mathrm{OOOCH}}_{2}{{\mathrm{OSO}}_{3}}^{-}\to •{\mathrm{OCH}}_{2}{{\mathrm{OSO}}_{3}}^{-}+O_{2}$$

Following its formation, the **IM2** faces a competition between unimolecular fragmentation (**R2**) and bimolecular addition of oxidants (O_2_/O_3_). The fragmentation pathway (**R2**), which involves S···O bond scission to yield HCHO and the SO_3_•^–^, exhibits a moderate barrier (Δ*G* ^‡^ = 76.7 kJ/mol). While accessible, this barrier is sufficiently high to render fragmentation a minor channel compared to the rapid interception by abundant molecular oxygen (**R3**) or ozone (**R10**) under typical atmospheric conditions. This interpretation is consistent with our earlier experimental findings,^18^ as well as prior theoretical calculations reported by Tchinda *et al*.^60^

The addition of O_2_ to **IM2** is the dominant fate of the radical, forming the peroxy intermediate (**IM3**, •OOCH_2_OSO_3_^–^). Notably, our calculations reveal a distinct feature of this organosulfate system: unlike simple alkyl radicals where O_2_ addition is typically barrierless,^66–68^ reaction R3 presents a calculated barrier of 11.5 kJ/mol (Figure 2). We trace the origin of this barrier to the electronic influence of the adjacent sulfate ester group. Spin population analysis (Figure 2b) reveals that in the transition state, the unpaired electron on the α-carbon is partially delocalized onto the sulfate oxygen atoms. This delocalization stabilizes the reactant radical and reduces the radical characteristic at the reactive carbon center. Despite this small barrier, the reaction remains highly exergonic (Δ*G* = −137.6 kJ/mol), ensuring the efficient conversion of **IM2** to **IM3** under atmospheric conditions. Alternatively, in ozone-rich environments, oxidation may proceed via an O_3_-mediated sequence (**R10** followed by **R11**).^21^ The O_3_-initiated route **R10** involves two-step mechanism: (1) interaction between •CH_2_OSO_3_^–^ and O_3_, and (2) unimolecular decomposition of the resulting **IM7** intermediate to form **IM6** with O_2_ (**R11**). While the initial interaction with O_3_ is barrierless (see Table S1 in the SI), the subsequent decomposition to the alkoxy radical (**IM6**) involves a small barrier (Δ*G* ^‡^= +3.8 kJ/mol). Although **R10** is kinetically facile, its atmospheric relevance is governed by the relative aqueous-phase abundances of O_2_ and O_3_. Using the Henry’s law constants^69^ at 298 K, $p_{O_{2}}=0.21\mathrm{atm}$ and $p_{O_{3}}=3.0\times 10^{-8}\mathrm{atm}$ (at 30 ppb). The pseudo-first-order rates are then $k_{3}^{’}=2.2\times 10^{6\;}s^{-1}$ , $k_{10}^{’}=2.4\;s^{-1}$ , and $k_{2}^{’}=0.24\;s^{-1}$ (Table S3 in the SI), so that **R3** outcompetes **R10** and **R2** by six and seven orders of magnitude, respectively. Even under heavily polluted conditions ([O_3_] up to 100 ppb), **R3** still dominates **R10** by more than 5 orders of magnitude. O_2_ addition **R3** therefore funnels essentially all the •CH_2_OSO_3_^–^ flux into peroxy-radical chemistry under tropospheric conditions. See the SI for more details.

**Figure 2:**
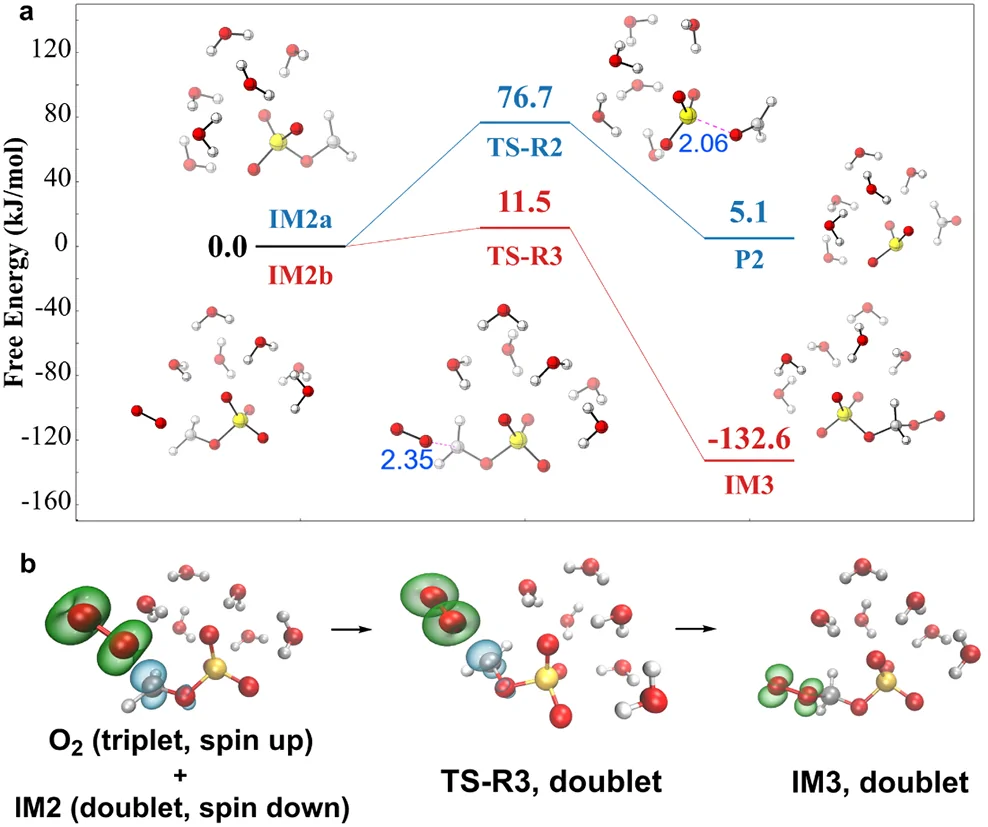
(a) Free energy profiles for the **R2** and **R3** steps. The zero of each reaction pathway was set to be the free energy of the corresponding materials. (b) Spin density isosurfaces at isovalue = 0.02 (green: α-spin, cyan: β-spin) in **R3**, showing the addition of molecular O_2_ to the IM2b. The product IM3 is a peroxy radical (•OOCH_2_OSO_3_^–^) in a doublet state.

### 4.2 Peroxy Radical (IM3, •OOCH_2_OSO_3_^–^): Fragmentation Vs Self-/Cross-Reactions

Once **IM3** is formed via O_2_ addition, its subsequent reactivity involves a kinetic competition between fragmentation (**R4**) and bimolecular reaction with coexisting radical partners. In the main text, we first consider the **IM3** + **IM3** self-reaction (**R5,** see Figure 3a) because **IM3** is generated *in situ* within the aqueous condensed phase from methyl sulfate.

R4 $$•{\mathrm{OOCH}}_{2}{{\mathrm{OSO}}_{3}}^{-}\to \mathrm{HCHO}+{\mathrm{SO}}_{3}{•}^{-}\cdot \cdot \cdot O_{2}\to \mathrm{HCHO}+{{\mathrm{SO}}_{5}•}^{-}$$

R5 2•OOCH2OSO3−→O3−SOCH2OOOOCH2OSO3−

S1 •OOCH2OSO3−+•OOCH3→O3−SOCH2OOOOCH3

S2 •OOCH2OSO3−+•OOH→O3−SOCH2OOOOH

**Figure 3:**
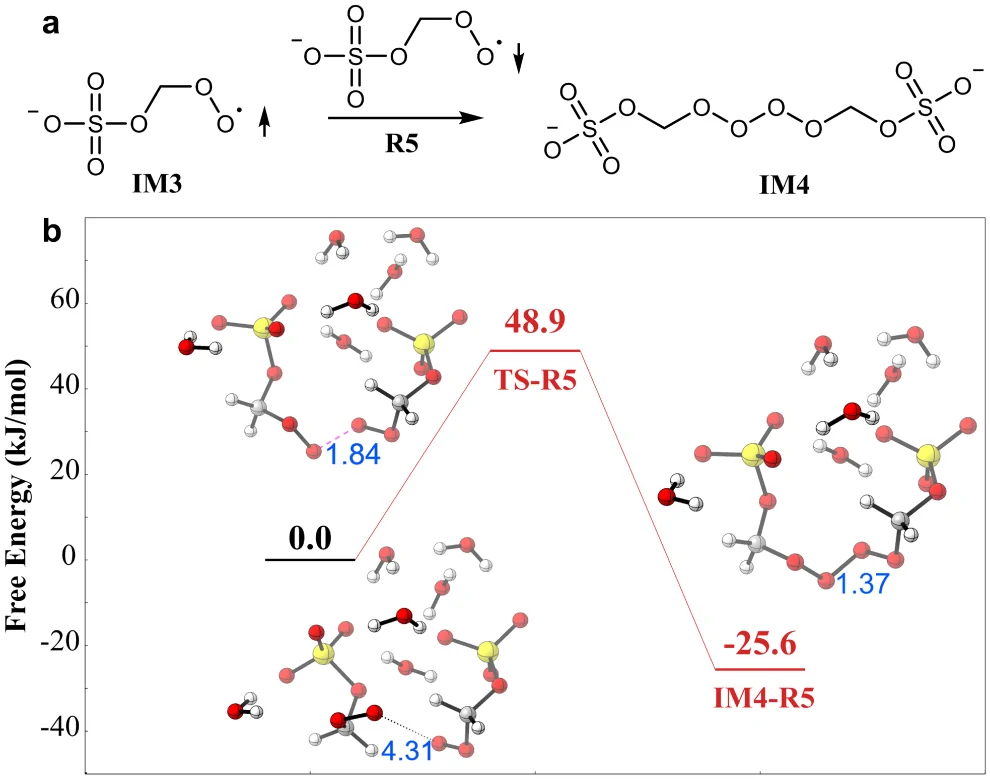
(a) Proposed self-reaction pathway for two IM3 peroxy radicals, leading to the formation of a peroxy disulfate ion intermediate (IM4). (b) Optimized structures of the reactant complex, transition state, and product are shown with key O–O bond distances in angstroms for **R5**.

Our calculations indicate that fragmentation to yield SO_3_•^–^ is energetically prohibitive. In the aqueous phase, strong hydrogen-bonding interactions between solvent water molecules and the sulfate ester group stabilize the **IM3** complex, raising the barrier for S···O bond scission to inaccessible levels (Δ*G* ^‡^ > 230 kJ/mol in gas phase; no transition state was located in solution). Consequently, the reaction flux is exclusively channeled toward the bimolecular self-/cross-reactions. The **IM3** + **IM3** self-reaction provides a mechanistically viable route to tetroxide intermediate (**IM4**, ^–^O_3_SOCH_2_OOOOCH_2_OSO_3_^–^) formation with a moderate barrier (Δ*G* ^‡^ = +48.9 kJ/mol) and favorable thermodynamics (Δ*G* = −25.6 kJ/mol) (Figure 3).

The decomposition of the tetroxide **IM4** represents a critical divergence point where the unique structural features of organosulfates dictate the reaction outcome. Previous computational studies have provided in-depth insights into peroxy radical reactions.^70–72^ In typical alkyl systems, the analog of intermediate **IM4** may undergo the following possible reaction mechanisms.

1. Bennett and Summers mechanism to form carbonyl-functionalized products.^61^

2. The classical Russell mechanism^62^ (six-membered, **R5** → **R6c**) to produce both alcohol- and carbonyl-functionalized products or Russell-type variants (**R5** → **R6a/b**).

3. Asymmetric two-step bond cleavage pathway, similar to that reported by Lee *et al.* in alkyl systems, yielding a caged intermediate of overall singlet spin multiplicity, comprising triplet oxygen and two alkoxy radicals (**R7** → **R8**).^73^

We investigated these three possible mechanisms for the decomposition of **IM4**. Neither the Bennett–Summers nor the classical Russell mechanisms appears viable for this organosulfate system. The Bennett–Summers pathway requires a bicyclic transition state comprising two fused five-membered rings Figure 4. In the case of **IM4**, due to the severe ring strain and electrostatic repulsion introduced by the bulky sulfate ester groups, this geometry is energetically inaccessible, consistent with findings by Tchinda *et al.*, in which the transition state was identified only in the gas phase, and a five-membered Russell-type variant in the solution phase was proposed as a more plausible alternative.^60^

**Figure 4:**
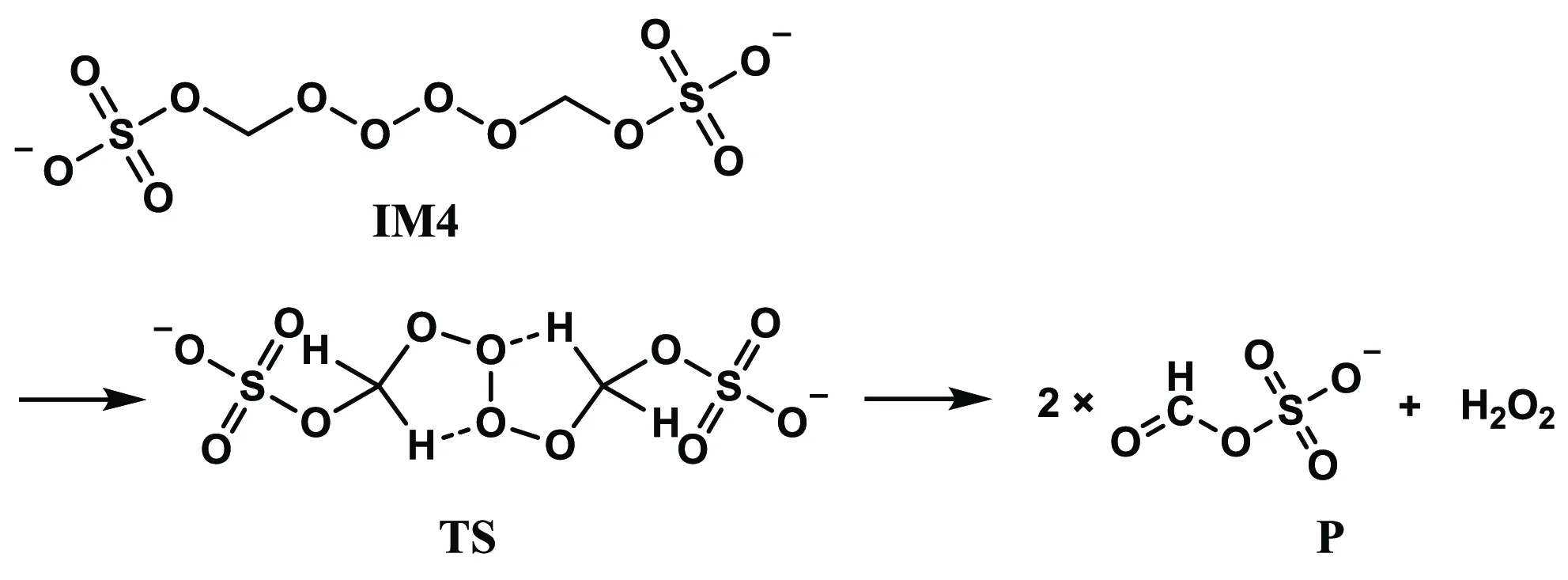
Proposed reaction pathways based on the Bennett and Summers mechanism.

Similarly, classical Russell rearrangements are effectively suppressed. As shown in Figure 5, we distinguish between the classical Russell mechanism (which generates carbonyl compounds, alcohols, and ^3^O_2_ via a six-membered cyclic TS) and the Russell-type variants (**R6a**/**R6b**) of **IM4**. The standard channel (**R6c**) could not be located for the solvated methyl sulfate tetroxide. We attribute this suppression to the geometric inability of the short alkyl backbone to accommodate the bulky sulfate groups within the requisite cyclic transition structures. For the Russell-type variants, **R6a** leads to a ketone and a hydrotrioxide intermediate (HOOOCH_2_OSO_3_^–^), while **R6b** forms an OO­(H)­O-functionalized organosulfate (OO­(H)­OCH_2_OSO_3_^–^) without direct alcohol formation. Additional calculations further show that the hydrotrioxide formed in the **R6b** channel undergoes O–O homolysis to **IM6** + HO_2_• (Δ*G* ^‡^ = 18.9 kJ/mol) rather than evolving toward alcohol formation (details in the SI **page** 10) Figure 6. Thus, even when this downstream step is considered, the overall Russell-type variant channels are still prohibited by the high **R6a**/**R6b** barrier (>128 kJ/mol, see Figure 7).

**Figure 5:**
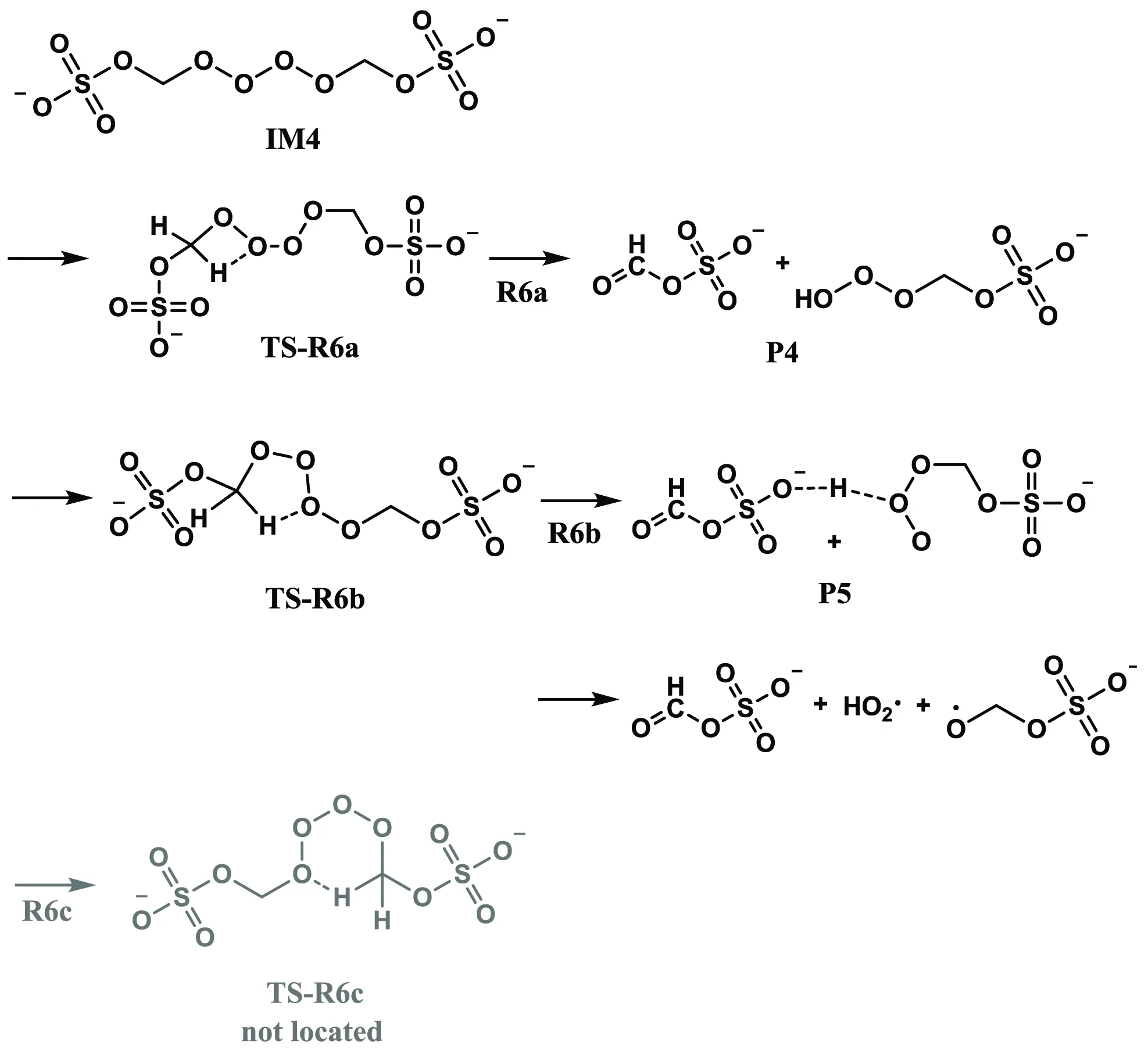
Proposed classical Russell (**R6c**) and Russell-type variants (**R6a**/**R6b**) pathways for decomposition of IM4.

**Figure 6:**
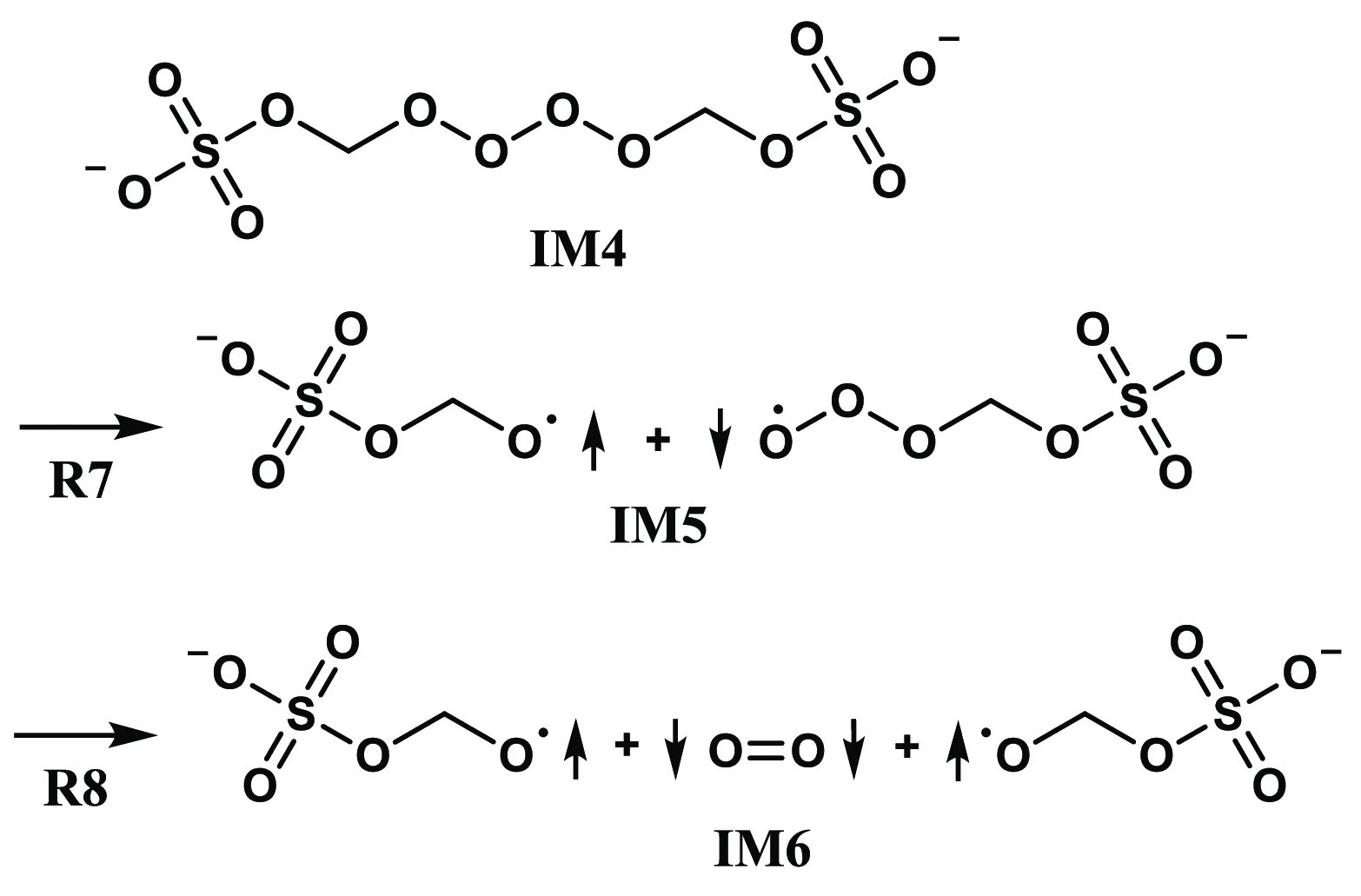
Proposed reaction pathways for **R7** → **R8**. IM4 undergoes consecutive single-bond cleavages, leading to the formation of an overall singlet spin-state.

**Figure 7:**
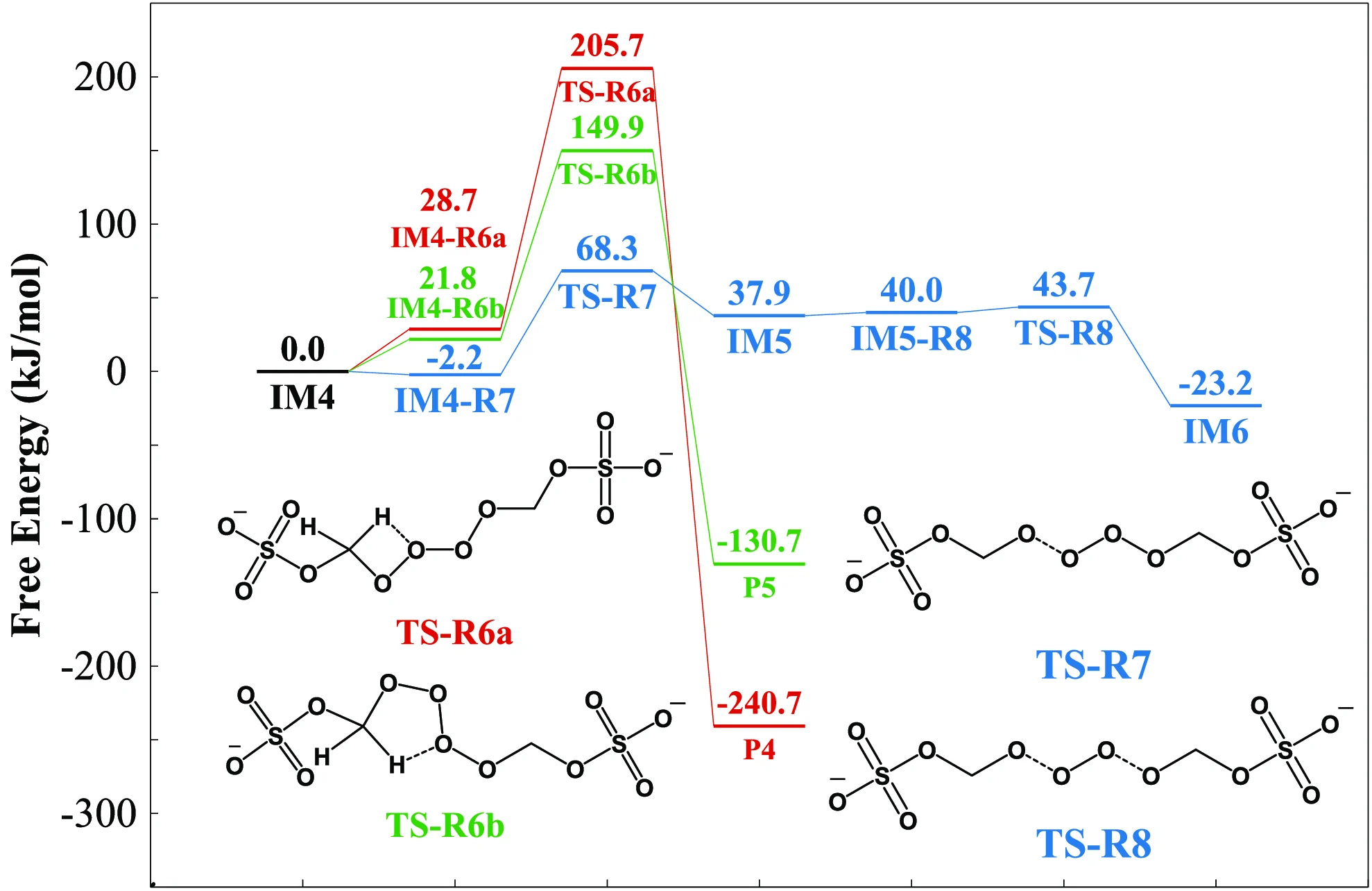
Free energy profiles of different reactions with IM4. Red and green paths correspond to Russell-type rearrangements, including four-membered (TS-**R6a**) and five-membered (TS-**R6b**) cyclic transition states, which exhibit prohibitively high barriers. The blue pathway represents the most probable route involving consecutive single-bond cleavages, leading to alkoxy sulfate radical (IM6) formation.

Instead, our results support an asymmetric two-step bond cleavage mechanism (**R5** → **R7** → **R8**), analogous to that reported for alkyl systems.^73^ This pathway bypasses the high-strain cyclic geometries, utilizing a singlet cage intermediate to yield alkoxy radicals (**IM6,** •OCH_2_OSO_3_^–^) and triplet molecular oxygen (^3^O_2_). The first step (**R7**) involves a homolytic cleavage of the O–O bond in **IM4**, generating a solvent-caged pair of **IM6** with overall singlet open shell cage containing ^–^_3_OSOCH_2_O•···•OOOCH_2_OSO_3_^–^. This step presents a moderate barrier (Δ*G* ^‡^ = +70.1 kJ/mol) and is endergonic (Δ*G* = +39.6 kJ/mol). Subsequently, the second step (**R8**) also occurs within the same overall singlet cage, involving cleavage of the remaining O–O bond in **IM5**, leading to the formation of ^3^O_2_ and two **IM6** radicals with the same spin state. The second step exhibits a significantly lower Δ*G* ^‡^ = +4.2 kJ/mol, indicating the potential for fast kinetics. Moreover, the formation of ^3^O_2_ is assisted by a substantial thermodynamic driving force (Δ*G* = −63.1 kJ/mol). The overall reaction rate constants further support this result: **R7** exhibits a rate constant of 8.8 s^–1^, and **R8** is so fast that it is diffusion-limited. Those results indicate that the **R5** → **R7** → **R8** pathway is both fast and favorable under ambient conditions.

Our calculations indicate that both the **Russell-type variants (R6a/R6b)** and **Bennett–Summers-type** pathways are unlikely to contribute significantly to the decomposition of peroxy radicals in this system. In contrast, our proposed stepwise O–O bond cleavage pathway (via **R7** and **R8**) proceeds through moderate barriers and yields thermodynamically stable alkoxy sulfate radicals and triplet O_2_. These results suggest that, under atmospheric or condensed-phase conditions, asymmetric bond cleavage pathways involving non-sulfur radicals are more likely to govern the oxidation chemistry of organosulfates. This finding aligns with the studies by Salo *et al.* and Zhang *et al.* ^72,74^ Moreover, no carbonyl-functionalized products were detected in our MS experiments, which is consistent with our theoretical predictions. Based on these findings, we propose that the two peroxy radicals undergo decomposition (via **R5** → **R7** → **R8**), leading to the formation of two alkoxy sulfate radicals (**IM6,** •OCH_2_OSO_3_^–^) and ^3^O_2_.

Although the **IM3** + **IM3** self-reaction was used above as the prototype RO_2_ + RO_2_ process, real aqueous aerosols contain mixed peroxy-radical populations. To examine the generality of the proposed RO_2_–RO_2_ mechanism in chemically complex aerosols and to assess whether the key tetroxide-mediated chemical reactions depend on the identity of the partners, we also investigated the cross-reactions (**S1 and S2**) of **IM3** with two representative peroxy radicals, CH_3_O_2_• and HO_2_• (most abundant peroxy radicals in the gas phase and aqueous aerosols, respectively). The calculations show that the mixed tetroxides formed from both **IM3** + CH_3_O_2_• and IM3 + HO_2_• mixed tetroxide remains capable of stepwise O–O bond cleavage to generate the same alkoxy sulfate radical **IM6**. Therefore, replacing one **IM3** by either CH_3_O_2_• or HO_2_• does not change the identity of the key sulfate-forming intermediate, although the co-reacting radical modulates the preferred cleavage direction and kinetic accessibility. These results support the generality of our conclusion that, whenever the tetroxide channel is accessible, the organosulfate-bearing terminus directs decomposition toward **IM6**. (Additional details are provided in the “Environmental relevance of **IM3** self-reaction and cross-reactions with atmospherically relevant peroxy radicals” section in the SI).

### 4.3 Alkoxy Sulfate Radical (IM6, •OCH_2_OSO_3_^–^) Fragmentation: The Water-Enabled Non-Sulfur Radical Pathways

After forming the **IM6**, two distinct channels are feasible, designated as a **non-sulfur radical** pathway (**R9a**/**R9b**) and a conventional sulfur-radical pathway (**R9c**). As illustrated in Figure 8, the mechanism distinction between the two channels lies in whether proton transfer occurs from the α-carbon to the sulfate oxygen, and where the radical character resides during C–OSO_3_^–^ bond cleavage. The non-sulfur radical pathway is associated with a heterolytic cleavage of the C–OSO_3_^–^ bond, in which the unpaired electron remains on the carbon fragment, yielding the formyl radical (**•**CHO) and HSO_4_^–^. Two proton-transfer modes are possible: a water-assisted route **R9a** and an intramolecular route **R9b**. By contrast, the sulfur-radical pathway **R9c** entails homolytic cleavage of the C–OSO_3_^–^ bond, with one electron remaining on each fragment. This results in a diradical intermediate (•CH_2_O•) and a SO_4_•^–^, followed by rapid intramolecular spin pairing to yield HCHO. Our DFT calculations have identified **R9a** as the thermodynamically and kinetically favored pathway, while **R9b** and **R9c** are less likely. Therefore, the discussion below focuses on **R9a**, while the **R9b** and **R9c** pathways are discussed in the SI.

**Figure 8:**
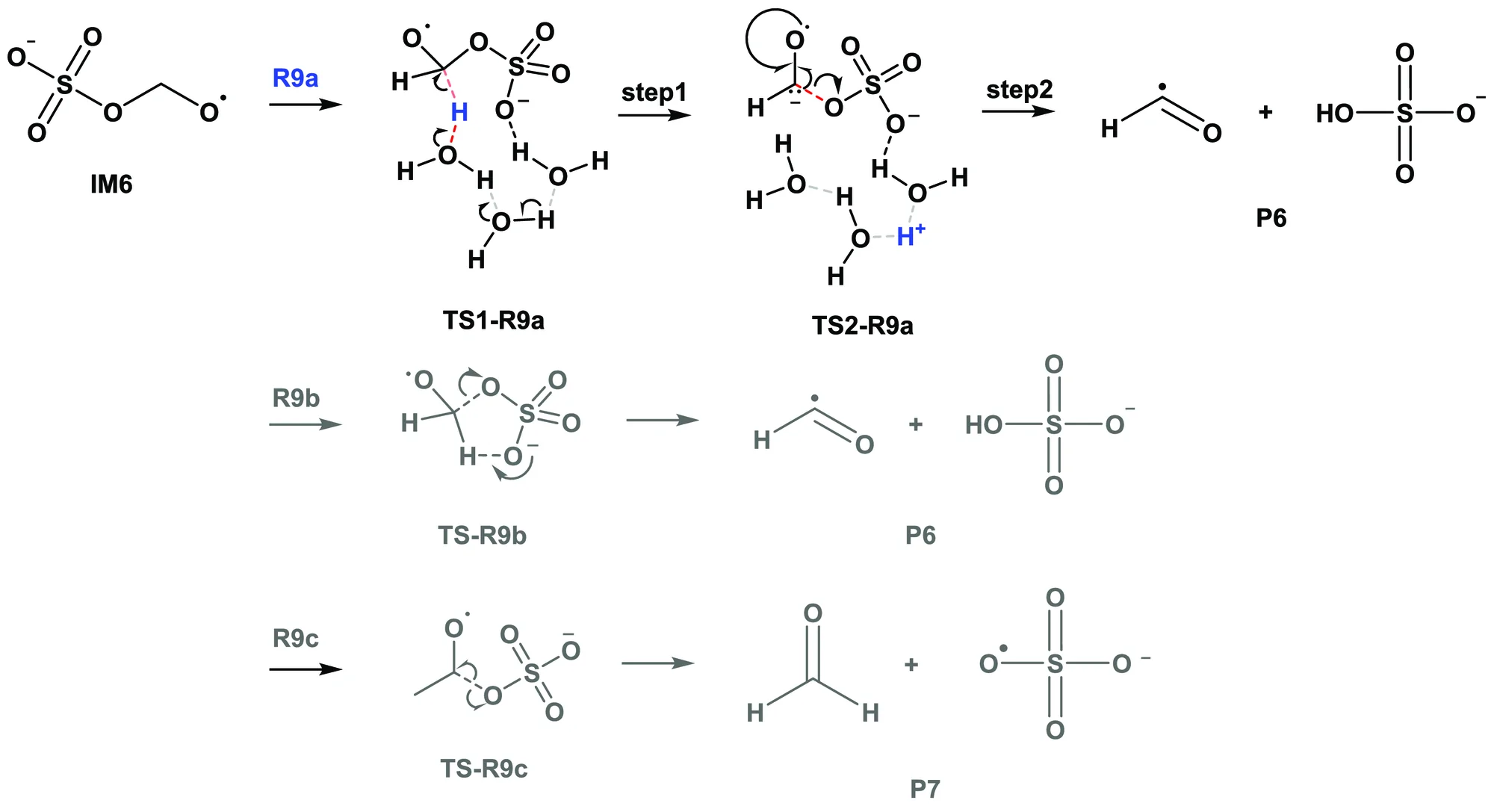
Proposed reaction mechanisms for the **R9a**, **R9b**, and **R9c** fragmentation pathways of the alkoxy sulfate radical IM6. **R9a** and **R9b** yield the carbon-centered formyl radical, whereas **R9c** yields sulfate radical anion.

When three or more water molecules are involved in the proton relay, **R9a** proceeds via **TS1-R9a** and **TS2-R9a**, a stepwise mechanism depicted in Figure 9 (the cases with fewer than 3 water molecules are shown in the SI). In this case, the proton is first relayed along a “water wire” to the sulfate ester group in step 1, forming a stabilized intermediate through **TS1-R9a**. This is followed by step 2, the C–OSO_3_^–^ bond cleavage and rapid electronic rearrangement occur, yielding **•**CHO and HSO_4_^–^. The decomposition of **IM6** via **R9a** is intrinsically driven by the aqueous environment. In the absence of explicit water molecules (or with insufficient hydration), the strain required to bridge the α-carbon proton to the sulfate oxygen is energetically costly. However, the inclusion of explicit water molecules opens a catalytic low-energy channel. Therefore, we systematically investigated **R9a** in the presence of the implicit SMD water model with 0–6 additional explicit water molecules. For this pathway, we also put 22 explicit water molecules in the calculations to form one solvation shell around the reactants to provide a closer representation of a bulk-like aqueous environment. We have labeled the transition states as **TS-R9a** ^**n**^, where n is the number of explicit water molecules. The case when *n* = 6 is shown in Figure 9.

**Figure 9:**
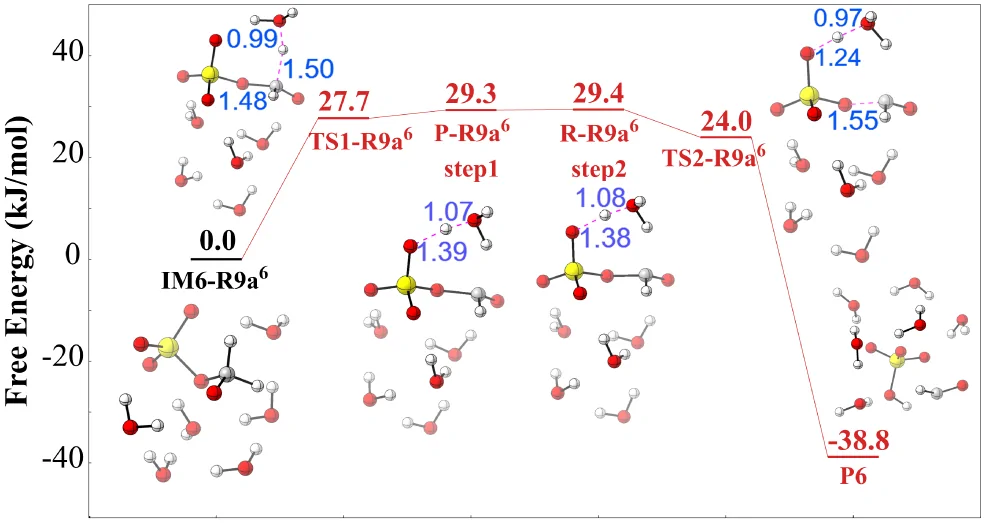
Free energy profiles for the water-assisted two-step R9a reaction pathway, illustrating •CHO and HSO_4_^–^ formation. Step 1 involves proton transfer through a hydrogen-bonded water wire to the sulfate ester group (Δ*G* ^‡^ = 27.7 kJ/mol), forming a stabilized intermediate. Step 2 proceeds with C–OSO_3_^–^ bond cleavage to yield •CHO and HSO_4_^–^. The overall barrier is about 29 kJ/mol. The overall reaction is thermodynamically favorable (Δ*G* = −38.8 kJ/mol).

For the water-assisted route, the transition state **TS1-R9a** ^**6**^ and **TS2-R9a** ^**6**^ displays a distinct geometry indicative of proton shuttling through a hydrogen-bonded “water wire”. Overall, the water-assisted mechanism features significantly lower (overall Δ*G* ^‡^ around +29.4 kJ/mol) compared to the intramolecular route. The overall reaction is highly exergonic, yielding **•**CHO and HSO_4_^–^ with Δ*G* = −38.8 kJ/mol. These findings highlight the critical role of water molecules in facilitating proton-coupled bond cleavage through a stepwise mechanism, lowering Δ*G* ^‡^ and stabilizing key intermediates compared to the concerted intramolecular route. Our simulations indicate that at least two bridging water molecules are required to form a viable **TS2-R9a** (Figure 8), though it is only observed when three or more explicit water molecules are included. Evidence of this shift is most clearly seen in the six-water system, where proton transfer and C–OSO_3_^–^ cleavage occur in two distinct steps. While the second step yields a well-defined transition state validated by IRC, it is effectively barrierless and highly exergonic. Specifically, the Δ*G* ^‡^ for the first step remains around 25 kJ/mol regardless of water number, but the second-step barrier decreases markedly from 13.1 kJ/mol (3 explicit waters) to barrierless (6 explicit waters), with Δ*G* reaching −68.2 kJ/mol. We listed all the Δ*G* and Δ*G* ^‡^ in Table 2 and Table S2.

**Table 2:** Computed Δ*G* ^‡^ and Δ*G* for the Proposed Proton Transfer and C–OSO_3_^–^ Bond Cleavage Steps along the **R9a** Reaction Pathways at 298 K and 1 Bar in Aqueous Phase^a^ ^b^

•OCH_2_–O–SO_3_^–^ + *n* H_2_O →**TS1/2-R9a** ^***n***^ → •CHO + HSO_4_^–^ + *n* H_2_O, where *n* = 3, 4, 5 and 6				
*n*	Step1 Δ*G* ^‡^ (kJ/mol)	Step1 Δ*G* (kJ/mol)	Step2 Δ*G* ^‡^ (kJ/mol)	Step2 Δ*G* (kJ/mol)
**3**	24.2	13.1	2.6	–50.3
**4**	21.1	20.3	6	–59.6
**5**	28.7	10.8	4.1	–51.1
**6**	27.7	28.6	barrierless	–68.2
•OCH_2_–O–SO_3_^–^ + *n* H_2_O → **TS1/2-R9a** ^**n**^ → •CHO + SO_4_^2–^ + H^+^ + *n* H_2_O, where *n* = 22				
22*	8.1	–4.0	6.3	–49.6

a The optimization level was set to be M06-2X-D3(0)/def2-SVP with SMD implicit water model to reduce computational cost.

b “*n*” stands for the number of explicit water molecules in the calculation.

We further increased the number of explicit water molecules to 22 at M06-2X-D3 (0)/ma-def2-QZVPP//M06-2X-D3(0)/def2-SVP level of theory with the SMD solvent model (shown in Figure 10). This model more accurately mimics the aqueous phase, where the **IM6** is fully solvated by water molecules. In this configuration, the extended water network facilitates multistep proton transfer from the α-carbon via **TS1-R9a** ^**22**^ and **TS2-R9a** ^**22**^. The first step (α-C deprotonation) has a low Δ*G* ^‡^ = 8.1 kJ/mol and a nearly negligible change in free energy (Δ*G* = −4.0 kJ/mol). The second step, corresponding to C–OSO_3_^–^ bond cleavage accompanied by proton migration within the water network, also exhibits a small barrier (Δ*G* ^‡^ = 6.3 kJ/mol) and is strongly exergonic (Δ*G* = −27.4 kJ/mol). Notably, the proton remains stabilized as a solvated proton and does not immediately associate with sulfate, leading to SO_4_^2–^ rather than HSO_4_^–^ as the instantaneous anionic product in this system. Subsequent partitioning between SO_4_^2–^ and HSO_4_^–^ is expected to be governed by local acid–base equilibration and buffering capacity in the aerosol aqueous phase. Relative to the 6-water system (Figure 9), the extended water network preferentially stabilizes the charge-separated precursor to C–O cleavage (**R-R9a** ^**22**^ **-step2**, −22.2 kJ/mol) over the proton-transfer intermediate (**P-R9a** ^**22**^ **-step1**, −4.0 kJ/mol), consistent with enhanced electrostatic stabilization by an extended hydrogen-bond network (Figure 10). The resulting profile is both kinetically accessible and thermodynamically favorable overall (Δ*G* = −49.6 kJ/mol), and the mechanistic distinction between **R9a** and **R9c** is evident from the corresponding spin density analysis (Figure 10a and Figure S3a). These low barriers further imply that, once **IM6** is formed, proton release can occur rapidly in aqueous aerosols, providing a direct route to aerosol acidification (lower pH).

**Figure 10:**
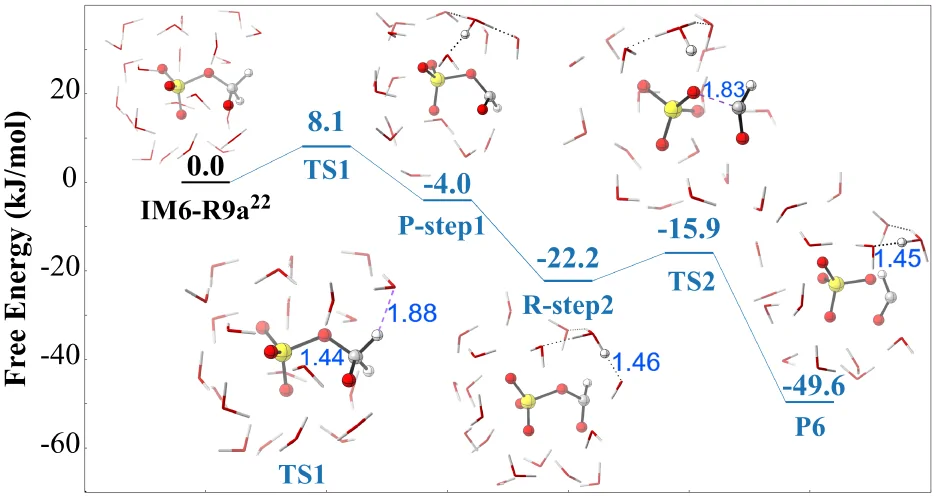
(a) Spin density isosurfaces at isovalue = 0.02 (green: α-spin). (b) Free energy profile of the R9a pathway with 22 explicit water molecules calculated at the M06-2X-D3(0)/ma-def2-QZVPP//M06-2X-D3(0)/def2-SVP level, along with SMD^38^ water model. Purple lines indicate forming or breaking bonds associated with key transition states, while black dashed lines represent hydrogen bonding interactions. For clarity, some water molecules have been omitted from the schematic illustration.

These results highlight that a progressively extended hydrogen-bonded water network plays a key catalytic role in the **IM6** fragmentation by enabling efficient proton transfer and stabilizing polar radical region along the reaction coordinate. This behavior in the **R9a-22w** system is consistent with the concepts of water-assisted reactions and closely parallels the catalytic function of water clusters in promoting the hydrolysis of SO_3_ to sulfuric acid.^75–77^ More broadly, we demonstrate that this effect is also evident in the aqueous phase. Our model confirms the viability of the pathway via **TS-R9a** ^**n**^ and underscores that explicit solvation is essential for accurately capturing organic-inorganic sulfate formation chemistry under aqueous aerosol conditions. Additionally, atmospheric aerosol particles can be substantially more acidic and less hydrated than the aqueous environment modeled above. We therefore investigated low water activity and low pH conditions for the **R9** pathways. These calculations confirm that **R9a** remains the kinetically and thermodynamically preferred channel and that the non-sulfur-radical route to inorganic sulfate is preserved across these conditions, although the barriers and the SO_4_^2–^/HSO_4_^–^ partitioning shift as expected. Details are provided in “Effects of Low Water Activity and Acidic Conditions on the **R9** Pathways” in the SI.

Collectively, our findings strongly support **R9a** as the dominant mechanism for inorganic sulfate formation from the methyl sulfate anion under aqueous aerosol conditions, providing new molecular-level insight into the *stepwise, water-assisted proton-coupled bond cleavage* mechanism. The resulting HSO_4_^–^/SO_4_^2–^ can subsequently transform into other inorganic sulfates, while the •CHO produced via **R9a** is expected to react rapidly with dissolved O_2_ to yield CO and HO_2_•.^78–80^ Unlike SO_4_•^–^, which would propagate aggressive radical chain chemistry within the aerosol, HO_2_• is a comparatively mild oxidant whose primary aqueous fate is disproportionation to H_2_O_2_. This distinction implies that the non-sulfur radical pathway leads to substantially less secondary organic oxidation within the aerosol phase. In our subsequent MS experiments, the •CHO or •CH_3_O (its protonated product after the addition of two protons) fragment suggested after the **R9a** step, was detected. In summary, our DFT calculations show the most probable route to form the inorganic sulfates in Scheme 2 is **R1b → R3 → R5 → R7 → R8 → R9a**, and **HSO**_**4**_ **^–^/SO**_**4**_^**2–**^ is the predominant product of the OH oxidation of methyl sulfate. This highlights an alternative **non-sulfur radical** pathway for sulfate generation in the aqueous phase, which may be more significant than previously recognized in atmospheric chemistry.

To assess whether this α-carbon pathway is specific to methyl sulfate or can also occur in larger organosulfates, we carried out additional calculations for a representative isoprene-derived organosulfate, with the OH attack initiated at the carbon adjacent to the sulfate ester group. The computed sequence follows the same key elementary steps identified for methyl sulfate, including α-C–H abstraction, O_2_ addition, formation of an alkoxy sulfate radical, and non-sulfur-radical formation of inorganic sulfate species. The corresponding barriers are comparable to those of methyl sulfate, supporting the relevance of the proposed mechanism for larger organosulfates when oxidation occurs at the sulfate-adjacent carbon. In addition, for the isoprene-derived organosulfate, we identified a barrierless β-scission channel of the alkoxy sulfate radical that is unavailable to methyl sulfate. This channel forms formyl sulfate, HCOOSO_3_^–^, which can subsequently hydrolyze to form HCOOH and HSO_4_^–^. Thus, although the detailed branching can differ in larger organosulfates, both channels converge toward inorganic sulfate formation through non-sulfur radical chemistry. Full computational details are provided in the SI.

### 4.4 *In Situ* EPR Detection of Sulfur Radicals and Reaction Intermediates in Aqueous-Phase OH Oxidation with Methyl Sulfate Ions

To identify the radical intermediates, we conducted *in situ* EPR spectroscopy using BMPO as the spin trap. Preliminary control experiments confirmed that BMPO is capable of trapping both **SO**_**3**_ **•** ^–^/**SO**_**4**_ **•** ^–^ and OH, although significant spectral overlap was observed between BMPO–SO_3_ and BMPO–OH adducts (Figure S11). In the methyl sulfate + H_2_O_2_ + UV system, the EPR spectra were dominated by an intense, time-dependent BMPO–OH signal, confirming the efficient generation of OH (Figure S12). While carbon-centered radicals became discernible after approximately 3 minutes of irradiation, distinct signals for sulfite or sulfate radicals (proposed in pathways **R2**/**R4** and **R9c**) were not observed. Based on our control experiments where both BMPO–OH and BMPO–SO_3_ were expected, the spectral congestion and the overwhelming intensity of the BMPO–OH signal mask the sulfur-centered radical peaks (Figure S11b). To overcome these resolution limitations and identify the specific adducts formed, we further characterized the reaction mixture using mass spectrometry.

### 4.5 MS Detection of Sulfur Radicals and Reaction Intermediates in Aqueous-Phase OH Oxidation with Methyl Sulfate Ions

High-resolution MS analysis of the reaction mixture (BMPO + methyl sulfate with/without H_2_O_2_ + UV) revealed several radical adducts formed after irradiation, as shown in Figure 11a, b. The primary product in negative ion mode, not observed in the control experiment, appears at m/z 308.081, which corresponds to an oxidized adduct ion ([M – H]^−^) of BMPO + CH_2_OSO_3_. This confirms the formation of the methyl sulfate radical, **IM2**, and quenches the trapped molecules, reducing the yield of radicals formed through later steps. Notably, the negative ion mode data did not contain any peaks corresponding to BMPO–Sulfite (m/z = 280.086) and very minor peaks corresponding to BMPO–Sulfate (m/z = 296.081), both of which were observed in the BMPO + Sulfite + UV control experiments (Figure S13). Additionally, direct injection MS was used to detect the ions below m/z 100 (Figure S14) and showed negligible HSO_3_^–^ and minimal change in HSO_4_^–^, consistent with disrupting the pathway by trapping **IM2**.

**Figure 11:**
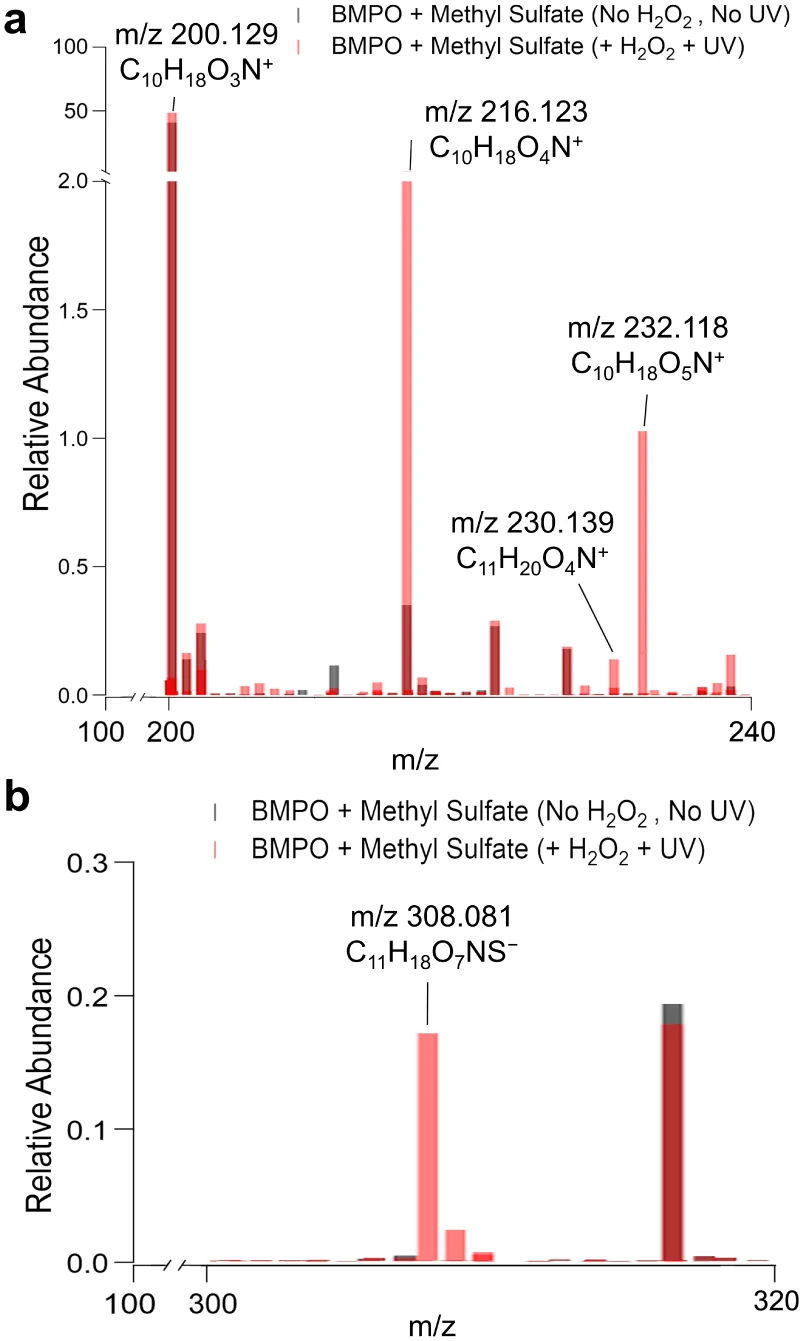
(a) Positive ion and (b) negative ion mode LCMS results for methyl sulfate + H_2_O_2_ + UV (red) and the dark control system (black) with major BMPO adducts labeled. Full MS spectra are available in the SI.

The positive ion mode analysis revealed many BMPO adducts including m/z = 216.123 (BMPO + •OH), 232.118 (BMPO + •OOH), expected products of H_2_O_2_ photolysis and m/z 230.139 (BMPO + •CH_3_O). As investigated in our previous work, BMPO adducts do not produce traditional [M + H]^+^ during electrospray ionization.^59^ Rather, they undergo disproportionation reactions in which a hydrogen atom is transferred from one radical to another producing an oxidized [M]^+^ adduct and a reduced [M + H] adduct, which ionizes into [M + 2H]^+^. This creates ambiguity in the identification of organic radical adducts, such as m/z 230.139 (BMPO + •CH_3_O) which can correspond to either the reduced form of BMPO + •CHO or oxidized BMPO + •CH_3_O. Following previously described methods,^59^ we examined the fragmentation mass spectrum (Figure S15) and found loss of both carboxylic acid and water functional groups, consistent with a reduced BMPO adduct. Additionally, the retention time of this adduct (7.97 minutes, Figure S16) is different from previously observed oxidized CH_3_O adducts detected using an identical LC system and method (10.4 minutes),^59^ further supporting that this adduct is the reduced form ([M + 2H]^+^) of BMPO + CHO. According to our proposed reaction mechanism, the formation of •CHO is only feasible via the **R9a** pathway, which involves the proton transfer decomposition of an alkoxy sulfate radical **(IM6)**. In contrast, the formation of •OCH_3_ would require the OH to directly attack the sulfate group via **R1a**, a process associated with a prohibitively high energy barrier (Δ*G* ^‡^ = 161.7 kJ/mol) as shown in our DFT calculations (Figure 1).

The above suggests that **R9a** is the predominant pathway for OH oxidation of methyl sulfate, which is in good agreement with our DFT calculations, confirming that pathways involving the generation of sulfur radicals are not significant (**R1a**, **R2**, **R4**, **and R9c**), whereas **R9a** is the key oxidation channel for the OH oxidation of methyl sulfate.

## 5 Conclusions

Understanding the atmospheric transformation of OSs is essential for elucidating their contributions to aerosol composition and sulfate cycling. In this study, we investigated the OH oxidation pathways of methyl sulfate, the smallest organosulfate detected in atmospheric aerosols, to understand the formation of inorganic sulfates by both QM calculations and aqueous-phase oxidation experiments. Our key finding is the identification and mechanistic characterization of a dominant non-sulfur radical pathway, **R1b** → **R3** → **R5** → **R7** → **R8** → **R9a**, leading to the formation of **•**CHO and HSO_4_^–^ as primary products. This pathway proceeds via a proton-coupled C–OSO_3_^–^ bond cleavage in the alkoxy radical intermediate (**IM6**), where we successfully modeled a more realistic aqueous aerosol environment using up to 22 explicit water molecules. We show that accurately capturing the reaction mechanism requires explicit inclusion of at least the first solvation shell of water molecules. The number of water molecules, and hence the extent of the hydrogen-bond network, has a direct influence on the stability of reaction intermediates and transition states. In particular, the inclusion of an extended hydrogen-bond network reveals a stepwise water-assisted mechanism, with a considerably lower free energy barrier (∼8 kJ/mol), indicating that this reaction can occur rapidly at ambient temperatures.

Beyond the dominant **R9a** channel, we explored and compared three key processes that govern the evolution of OS-derived radicals: self-decomposition, reaction with O_2_, and reaction with O_3_. Our DFT analysis confirms that both O_2_ and O_3_ can react with the **•**CH_2_OSO_3_^–^ radical (**IM2**) to form alkoxy radicals. Notably, the O_2_ addition exhibits a small but non-negligible activation barrier, which we attribute to spin density delocalization from the α-carbon onto the sulfate group, reducing the localized radical character and thus attenuating reactivity. This contrasts with the barrierless addition typically observed in unsubstituted alkyl radicals and reveals an electronic influence by the sulfate group. Furthermore, although the Russell mechanism and Bennett–Summers mechanism are well-established for peroxy radical reactions, our calculations suggest that these pathways are kinetically inaccessible in the case of methyl sulfate due to severe steric hindrance and electrostatic repulsion induced by the sulfate groups. Instead, the system favors an asymmetric two-step bond cleavage pathway, which is both kinetically and thermodynamically favorable under atmospheric conditions.

In closing, it is important to discuss the generality of the proposed mechanism for other atmospherically relevant OS molecules. To this end, we have carried out additional calculations for one representative isoprene-derived organosulfate, and the results are available in the SI. We found that the pathways and barriers are comparable to those of methyl sulfate when the attack occurs at the α-carbon relative to the sulfate group. However, as the OS becomes more complex, additional attack positions become available that can lead to other mechanisms. This warrants further work on larger OS molecules and possibly on those with different functional groups. Such efforts will be crucial for accurately predicting the fate of structurally diverse organosulfates in atmospheric aerosols and understanding their broader impacts on air quality and climate.

## Supplementary Material



## Notes

Supplementary tables and figures; EPR settings; EPR detection results; MS detection results; oxidation kinetic calculations; Cartesian coordinates of all species (PDF)

## References

[1] Tolocka M. P., Turpin B. (2012). Contribution of Organosulfur Compounds to Organic Aerosol
Mass. Environ. Sci. Technol..

[2] Surratt J. D., Gómez-González Y., Chan A. W. H., Vermeylen R., Shahgholi M., Kleindienst T. E., Edney E. O., Offenberg J. H., Lewandowski M., Jaoui M. (2008). Organosulfate Formation
in Biogenic Secondary Organic Aerosol. J. Phys.
Chem. A.

[3] Shakya K. M., Peltier R. E. (2013). Investigating Missing
Sources of Sulfur at Fairbanks,
Alaska. Environ. Sci. Technol..

[4] Shakya K. M., Peltier R. E. (2015). Non-Sulfate Sulfur
in Fine Aerosols across the United
States: Insight for Organosulfate Prevalence. Atmos. Environ..

[5] Hettiyadura A. P. S., Al-Naiema I. M., Hughes D. D., Fang T., Stone E. A. (2019). Organosulfates
in Atlanta, Georgia: Anthropogenic Influences on Biogenic Secondary
Organic Aerosol Formation. Atmos. Chem. Phys..

[6] Chen Y., Dombek T., Hand J., Zhang Z., Gold A., Ault A. P., Levine K. E., Surratt J. D. (2021). Seasonal Contribution
of Isoprene-Derived Organosulfates to Total Water-Soluble Fine Particulate
Organic Sulfur in the United States. ACS Earth
Space Chem..

[7] Brüggemann M., van Pinxteren D., Wang Y., Yu J. Z., Herrmann H. (2019). Quantification
of Known and Unknown Terpenoid Organosulfates in PM10 Using Untargeted
LC–HRMS/MS: Contrasting Summertime Rural Germany and the North
China Plain. Environ. Chem..

[8] Hughes D. D., Christiansen M. B., Milani A., Vermeuel M. P., Novak G. A., Alwe H. D., Dickens A. F., Pierce R. B., Millet D. B., Bertram T. H. (2021). PM2.5 Chemistry, Organosulfates, and Secondary
Organic Aerosol during the 2017 Lake Michigan Ozone Study. Atmos. Environ..

[9] Wang Y., Ren J., Huang X. H. H., Tong R., Yu J. Z. (2017). Synthesis of Four
Monoterpene-Derived Organosulfates and Their Quantification in Atmospheric
Aerosol Samples. Environ. Sci. Technol..

[10] Wang Y., Hu M., Guo S., Wang Y., Zheng J., Yang Y., Zhu W., Tang R., Li X., Liu Y. (2018). The Secondary
Formation of Organosulfates under Interactions between Biogenic Emissions
and Anthropogenic Pollutants in Summer in Beijing. Atmos. Chem. Phys..

[11] Wang Y., Ma Y., Kuang B., Lin P., Liang Y., Huang C., Yu J. Z. (2022). Abundance of Organosulfates
Derived from Biogenic Volatile Organic
Compounds: Seasonal and Spatial Contrasts at Four Sites in China. Sci. Total Environ..

[12] Yang T., Xu Y., Ma Y.-J., Wang Y.-C., Yu J. Z., Sun Q.-B., Xiao H.-W., Xiao H.-Y., Liu C.-Q. (2024). Field Evidence for
Constraints of Nearly Dry and Weakly Acidic Aerosol Conditions on
the Formation of Organosulfates. Environ. Sci.
Technol. Lett..

[13] Brüggemann M., Xu R., Tilgner A., Kwong K. C., Mutzel A., Poon H. Y., Otto T., Schaefer T., Poulain L., Chan M. N. (2020). Organosulfates in Ambient
Aerosol: State of Knowledge and Future
Research Directions on Formation, Abundance, Fate, and Importance. Environ. Sci. Technol..

[14] Fan W., Chen T., Zhu Z., Zhang H., Qiu Y., Yin D. (2022). A Review of Secondary Organic Aerosols Formation Focusing on Organosulfates
and Organic Nitrates. J. Hazard. Mater..

[15] Hu K. S., Darer A. I., Elrod M. J. (2011). Thermodynamics and
Kinetics of the
Hydrolysis of Atmospherically Relevant Organonitrates and Organosulfates. Atmos. Chem. Phys..

[16] Darer A. I., Cole-Filipiak N. C., O’Connor A. E., Elrod M. J. (2011). Formation and Stability
of Atmospherically Relevant Isoprene-Derived Organosulfates and Organonitrates. Environ. Sci. Technol..

[17] Craig R. L., Peterson P. K., Nandy L., Lei Z., Hossain M. A., Camarena S., Dodson R. A., Cook R. D., Dutcher C. S., Ault A. P. (2018). Direct Determination of Aerosol pH: Size-Resolved Measurements
of Submicrometer and Supermicrometer Aqueous Particles. Anal. Chem..

[18] Kwong K. C., Chim M. M., Davies J. F., Wilson K. R., Chan M. N. (2018). Importance
of Sulfate Radical Anion Formation and Chemistry in Heterogeneous
OH Oxidation of Sodium Methyl Sulfate, the Smallest Organosulfate. Atmos. Chem. Phys..

[19] Lam H. K., Kwong K. C., Poon H. Y., Davies J. F., Zhang Z., Gold A., Surratt J. D., Chan M. N. (2019). Heterogeneous
OH
Oxidation of Isoprene-Epoxydiol-Derived Organosulfates: Kinetics,
Chemistry and Formation of Inorganic Sulfate. Atmos. Chem. Phys..

[20] Chen Y., Zhang Y., Lambe A. T., Xu R., Lei Z., Olson N. E., Zhang Z., Szalkowski T., Cui T., Vizuete W. (2020). Heterogeneous Hydroxyl Radical Oxidation
of Isoprene-Epoxydiol-Derived Methyltetrol Sulfates: Plausible Formation
Mechanisms of Previously Unexplained Organosulfates in Ambient Fine
Aerosols. Environ. Sci. Technol. Lett..

[21] Xu R., Ge Y., Kwong K. C., Poon H. Y., Wilson K. R., Yu J. Z., Chan M. N. (2020). Inorganic
Sulfur Species Formed upon Heterogeneous
OH Oxidation of Organosulfates: A Case Study of Methyl Sulfate. ACS Earth Space Chem..

[22] Xu R., Ng S. I. M., Chow W. S., Wong Y. K., Wang Y., Lai D., Yao Z., So P.-K., Yu J. Z., Chan M. N. (2022). Chemical
Transformation of α-Pinene-Derived Organosulfate via Heterogeneous
OH Oxidation: Implications for Sources and Environmental Fates of
Atmospheric Organosulfates. Atmos. Chem. Phys..

[23] Xu R., Chen Y., Ng S. I. M., Zhang Z., Gold A., Turpin B. J., Ault A. P., Surratt J. D., Chan M. N. (2024). Formation
of Inorganic Sulfate and Volatile Nonsulfated Products from Heterogeneous
Hydroxyl Radical Oxidation of 2-Methyltetrol Sulfate Aerosols: Mechanisms
and Atmospheric Implications. Environ. Sci.
Technol. Lett..

[24] Madeleine
Ng S. I., Ng K. H., Felix Yeung P. W., Xu R., So P.-K., Huang Y., Yu J. Z., Choi C. K. K., Steve Tse Y.-L., Chan M. N. (2022). Chemical Transformation of a Long-Chain
Alkyl Organosulfate via Heterogeneous OH Oxidation: A Case Study of
Sodium Dodecyl Sulfate. Environ. Sci.: Atmos..

[25] Ng S. I. M., Chan M. N. (2023). Beyond the Formation:
Unveiling the Atmospheric Transformation
of Organosulfates via Heterogeneous OH Oxidation. Chem. Commun..

[26] Hansen A. M. K., Hong J., Raatikainen T., Kristensen K., Ylisirniö A., Virtanen A., Petäjä T., Glasius M., Prisle N. L. (2015). Hygroscopic Properties and Cloud
Condensation Nuclei Activation of Limonene-Derived Organosulfates
and Their Mixtures with Ammonium Sulfate. Atmos.
Chem. Phys..

[27] Riva M., Chen Y., Zhang Y., Lei Z., Olson N. E., Boyer H. C., Narayan S., Yee L. D., Green H. S., Cui T. (2019). Increasing Isoprene Epoxydiol-to-Inorganic Sulfate
Aerosol Ratio Results in Extensive Conversion of Inorganic Sulfate
to Organosulfur Forms: Implications for Aerosol Physicochemical Properties. Environ. Sci. Technol..

[28] Bain A., Chan M. N., Bzdek B. R. (2023). Physical Properties of Short Chain
Aqueous Organosulfate Aerosol. Environ. Sci.:
Atmos..

[29] Hettiyadura A. P. S., Stone E. A., Kundu S., Baker Z., Geddes E., Richards K., Humphry T. (2015). Determination of Atmospheric Organosulfates
Using HILIC Chromatography with MS Detection. Atmos. Meas. Tech..

[30] Hayon E. M., Treinin A., Wilf J. (1972). Electronic Spectra, Photochemistry,
and Autoxidation Mechanism of the Sulfite-Bisulfite-Pyrosulfite Systems.
SO2-, SO3-, SO4-, and SO5- Radicals. J. Am.
Chem. Soc..

[31] Tang Y., Thorn R. P., Mauldin R. L., Wine P. H. (1988). Kinetics and Spectroscopy
of the SO4– Radical in Aqueous Solution. J. Photochem. Photobiol., A.

[32] Huie R. E., Clifton C. L. (1989). Rate Constants for Hydrogen Abstraction
Reactions of
the Sulfate Radical, SO4–. Alkanes and Ethers. Int. J. Chem. Kinet..

[33] Huie R. E., Clifton C. L., Neta P. (1991). Electron Transfer Reaction Rates
and Equilibria of the Carbonate and Sulfate Radical Anions. Int. J. Radiat. Appl. Instrum. Part C.

[34] Das T. N. (2001). Reactivity
and Role of SO 5 •- Radical in Aqueous Medium Chain Oxidation
of Sulfite to Sulfate and Atmospheric Sulfuric Acid Generation. J. Phys. Chem. A.

[35] Herrmann H., Hoffmann D., Schaefer T., Bräuer P., Tilgner A. (2010). Tropospheric Aqueous-Phase Free-Radical Chemistry:
Radical Sources, Spectra, Reaction Kinetics and Prediction Tools. ChemPhyschem.

[36] Lee J., von Gunten U., Kim J.-H. (2020). Persulfate-Based Advanced Oxidation:
Critical Assessment of Opportunities and Roadblocks. Environ. Sci. Technol..

[37] Xu R., Lam H. K., Wilson K. R., Davies J. F., Song M., Li W., Tse Y.-L. S., Chan M. N. (2020). Effect of Inorganic-to-Organic Mass
Ratio on the Heterogeneous OH Reaction Rates of Erythritol: Implications
for Atmospheric Chemical Stability of 2-Methyltetrols. Atmos. Chem. Phys..

[38] Marenich A. V., Cramer C. J., Truhlar D. G. (2009). Universal Solvation
Model Based on
Solute Electron Density and on a Continuum Model of the Solvent Defined
by the Bulk Dielectric Constant and Atomic Surface Tensions. J. Phys. Chem. B.

[39] Gao B., Liu Z. (2004). A First Principles Study on the Solvation and Structure of SO42–(H2O)­n,
N=6–12. J. Chem. Phys..

[40] Vchirawongkwin V., Rode B. M., Persson I. (2007). Structure
and Dynamics of Sulfate
Ion in Aqueous SolutionAn Ab Initio QMCF MD Simulation and Large Angle
X-Ray Scattering Study. J. Phys. Chem. B.

[41] Wong R. L., Williams E. R. (2003). Dissociation of
SO42-(H2O)­n Clusters, n = 3–17. J. Phys.
Chem. A.

[42] Bach R. D., Dmitrenko O., Estévez C. M. (2003). Theoretical Analysis of Peroxynitrous
Acid: Characterization of Its Elusive Biradicaloid (HO···ONO)
Singlet States. J. Am. Chem. Soc..

[43] Bach R. D., Dmitrenko O., Estévez C. M. (2005). Chemical Behavior of the Biradicaloid
(HO···ONO) Singlet States of Peroxynitrous Acid. The
Oxidation of Hydrocarbons, Sulfides, and Selenides. J. Am. Chem. Soc..

[44] Lu, T. Molclus Program, Version 1.12. http://www.keinsci.com/research/molclus.html. (Accessed 23 Aug 2023)

[45] Frisch, M. J. ; Trucks, G. W. ; Schlegel, H. B. ; Scuseria, G. E. ; Robb, M. A. ; Cheeseman, J. R. ; Scalmani, G. ; Barone, V. ; Petersson, G. A. ; Nakatsuji, H. Gaussian16 Revision C.02; Gaussian Inc. 2016

[46] Zhao Y., Truhlar D. G. (2008). The M06 Suite of
Density Functionals for Main Group
Thermochemistry, Thermochemical Kinetics, Noncovalent Interactions,
Excited States, and Transition Elements: Two New Functionals and Systematic
Testing of Four M06-Class Functionals and 12 Other Functionals. Theor. Chem. Acc..

[47] Grimme S., Antony J., Ehrlich S., Krieg H. (2010). A Consistent
and Accurate
Ab Initio Parametrization of Density Functional Dispersion Correction
(DFT-D) for the 94 Elements H-Pu. J. Chem. Phys..

[48] Weigend F., Ahlrichs R. (2005). Balanced Basis Sets of Split Valence, Triple Zeta Valence
and Quadruple Zeta Valence Quality for H to Rn: Design and Assessment
of Accuracy. Phys. Chem. Chem. Phys..

[49] Fukui K. (1981). The Path of
Chemical Reactions - the IRC Approach. Acc.
Chem. Res..

[50] Zheng J., Xu X., Truhlar D. G. (2011). Minimally
Augmented Karlsruhe Basis Sets. Theor. Chem.
Acc..

[51] Zhao Y., Schultz N. E., Truhlar D. G. (2006). Design
of Density Functionals by
Combining the Method of Constraint Satisfaction with Parametrization
for Thermochemistry, Thermochemical Kinetics, and Noncovalent Interactions. J. Chem. Theory Comput..

[52] Rassolov V. A., Pople J. A., Ratner M. A., Windus T. L. (1998). 6-31G* Basis Set
for Atoms K through Zn. J. Chem. Phys..

[53] Ho J., Klamt A., Coote M. L. (2010). Comment
on the Correct Use of Continuum
Solvent Models. J. Phys. Chem. A.

[54] Lu T., Chen Q. (2021). Shermo: A General Code
for Calculating Molecular Thermochemistry
Properties. Comput. Theor. Chem..

[55] Grimme S. (2012). Supramolecular
Binding Thermodynamics by Dispersion-Corrected Density Functional
Theory. Chem. Eur. J..

[56] Legault, C. Y. CYLview, 2.0b; Université de Sherbrooke, 2020. https://www.cylview.org.

[57] Humphrey W., Dalke A., Schulten K. (1996). VMD: visual molecular
dynamics. J. Mol. Graph..

[58] Li, Y. Energy Diagram Plotter (CDXML); Zenodo 2023.

[59] Gerritz L., Perraud V., Weber K. M., Shiraiwa M., Nizkorodov S. A. (2024). Application
of UHPLC-ESI-MS/MS to Identify Free Radicals via Spin Trapping with
BMPO. J. Phys. Chem. A.

[60] Tsona
Tchinda N., Lv X., Tasheh S. N., Ghogomu J. N., Du L. (2025). Atmospheric Fate of Organosulfates through Gas-Phase and Aqueous-Phase
Reactions with Hydroxyl Radicals: Implications for Inorganic Sulfate
Formation. Atmos. Chem. Phys..

[61] Bennett J. E., Summers R. (1974). Product Studies of
the Mutual Termination Reactions
of Sec-Alkylperoxy Radicals: Evidence for Non-Cyclic Termination. Can. J. Chem..

[62] Russell G. A. (1957). Deuterium-Isotope
Effects in the Autoxidation of Aralkyl Hydrocarbons. Mechanism of
the Interaction of Peroxy Radicals^1^. J. Am. Chem. Soc..

[63] Kopinke F.-D., Georgi A. (2017). What Controls Selectivity of Hydroxyl Radicals in Aqueous
Solution? Indications for a Cage Effect. J.
Phys. Chem. A.

[64] Guo C., Xu L., Zhang C. (2022). Study on Heterogeneous OH Oxidation
of 3-Methyltetraol
Sulfate in the Atmosphere under High NO Conditions. RSC Adv..

[65] Tully F. P., Droege A. T., Koszykowski M. L., Melius C. F. (1986). Hydrogen-Atom Abstraction
from Alkanes by Hydroxyl. 2. Ethane. J. Phys.
Chem..

[66] Zádor J., Taatjes C. A., Fernandes R. X. (2011). Kinetics
of Elementary Reactions
in Low-Temperature Autoignition Chemistry. Prog.
Energy Combust. Sci..

[67] Lahm M. E., Bartlett M. A., Liang T., Pu L., Allen W. D., Schaefer H. F. (2023). The Multichannel I-Propyl + O2 Reaction
System: A Model
of Secondary Alkyl Radical Oxidation. J. Chem.
Phys..

[68] Duan J., Ji J., Ye L., Meng Q., Zhai Y., Zhang L. (2020). Theoretical
Calculation of Low-Temperature Oxidation of Heptyl Radicals and O2. Combust. Flame..

[69] Sander R. (2015). Compilation
of Henry’s law constants (version 4.0) for water as solvent. Atmos. Chem. Phys..

[70] Bohr F., Henon E., García I., Castro M. (1999). Theoretical Study of
the Peroxy Radicals RO2 Self-Reaction: Structures and Stabilization
Energies of the Intermediate RO4R for Various R. Int. J. Quantum Chem..

[71] Shallcross D. E., Raventos-Duran M. T., Bardwell M. W., Bacak A., Solman Z., Percival C. J. (2005). A Semi-Empirical
Correlation for the Rate Coefficients
for Cross- and Self-Reactions of Peroxy Radicals in the Gas-Phase. Atmos. Environ..

[72] Salo V.-T., Valiev R., Lehtola S., Kurtén T. (2022). Gas-Phase
Peroxyl Radical Recombination Reactions: A Computational Study of
Formation and Decomposition of Tetroxides. J.
Phys. Chem. A.

[73] Lee R., Gryn’ova G., Ingold K. U., Coote M. L. (2016). Why Are
Sec-Alkylperoxyl
Bimolecular Self-Reactions Orders of Magnitude Faster than the Analogous
Reactions of Tert-Alkylperoxyls? The Unanticipated Role of CH Hydrogen
Bond Donation. Phys. Chem. Chem. Phys..

[74] Zhang P., Wang W., Zhang T., Chen L., Du Y., Li C., Lü J. (2012). Theoretical
Study on the Mechanism and Kinetics for
the Self-Reaction of C2H5O2 Radicals. J. Phys.
Chem. A.

[75] Larson L.
J., Kuno M., Tao F.-M. (2000). Hydrolysis of Sulfur Trioxide to
Form Sulfuric Acid in Small Water Clusters. J. Chem. Phys.

[76] Morokuma K., Muguruma C. (1994). Ab Initio Molecular Orbital Study of the Mechanism
of the Gas Phase Reaction SO3 + H2O: Importance of the Second Water
Molecule. J. Am. Chem. Soc..

[77] Loerting T., Liedl K. R. (2000). Toward Elimination of Discrepancies
between Theory
and Experiment: The Rate Constant of the Atmospheric Conversion of
SO3 to H2SO4. Proc. Natl. Acad. Sci. U. S. A..

[78] Timonen R. S., Ratajczak E., Gutman D. (1988). Kinetics of the Reactions of the
Formyl Radical with Oxygen, Nitrogen Dioxide, Chlorine, and Bromine. J. Phys. Chem..

[79] Nesbitt F. L., Gleason J. F., Stief L. J. (1999). Temperature Dependence of the Rate
Constant for the Reaction HCO + O2 → HO2 + CO at T = 200–398
K. J. Phys. Chem. A.

[80] Faßheber N., Friedrichs G., Marshall P., Glarborg P. (2015). Glyoxal Oxidation Mechanism:
Implications for the Reactions HCO + O2 and OCHCHO + HO2. J. Phys. Chem. A.

