# Supplementary material for: Elucidating Formation Mechanisms of Inorganic Sulfates upon OH Radical Oxidation of Organosulfates via Non-Sulfur Radical Pathways: Insights from Methyl Sulfate
Source: J Am Chem Soc. 2026 Jun 24;148(35):37663–76. doi: 10.1021/jacs.6c01898 (PMC13564445; doi:10.1021/jacs.6c01898)
Supplement: Supplementary file 1 [file ja6c01898_si_001.pdf]

Supporting Information of

Elucidating formation mechanisms of inorganic sulfates upon OH radical oxidation of organosulfates via non-sulfur radical pathways: insights from methyl sulfate

Tianren Zhu[1], Sheung Ki Suki Chan[2], Lena Gerritz[3], Sergey A. Nizkorodov[3*], Manabu Shiraiwa[3*], Man Nin Chan[2*], Ying-Lung Steve Tse[1*]

[1] Department of Chemistry, The Chinese University of Hong Kong, Sha Tin, Hong Kong, China
[2] Department of Earth and Environmental Sciences, The Chinese University of Hong Kong, Sha Tin, Hong Kong, China
[3] Department of Chemistry, University of California, Irvine, Irvine, California 92697, United States

Table of Contents

**2**	**Computed free energy barriers $\Delta G^{\ddagger}$ and Gibbs free energy changes ΔG for all proposed reaction pathways**
**3-7**	**Generality test: OH oxidation of an isoprene-derived organosulfate**
**8**	**Spin density analysis for R1a and R1b**
**9**	**Pseudo-first-order rate constants for the competition between R2, R3, and R10**
**10**	**Post-R6b decomposition of the Hydrotrioxide**
**11-16**	**Environmental relevance of IM3 self-reaction and cross-reactions with atmospherically relevant peroxy radicals**
**17-20**	**Alkoxy sulfate radical (•$OCH_2OSO_3^-$): Fragmentation (minor R9b and R9c pathways)**
**21-24**	**Effects of Low Water Activity and Acidic Conditions on the R9 Pathways**
**25**	**Electron paramagnetic resonance spectroscopy settings**
**25**	**Radical generation**
**26**	**EPR detection results: Validation of BMPO for radical species (SO_3•⁻/ SO_4•⁻ and OH)**
**27**	**EPR detection results for the methyl sulfate system**
**27**	**Assessment of sulfite/sulfate radical formation**
**28-29**	**MS detection results: Validation of BMPO for radical species (SO_3•⁻/ SO_4•⁻)**
**30-32**	**MS detection results: BMPO + methyl sulfate + H_2O_2 system with/without UV**
**33-34**	**High-resolution Figure 11a and Figure 11b**
**35-37**	**Oxidation Kinetics: Rate Constants for the Kinetic Model at 298 K**
**38-73**	**Cartesian coordinates of all species**

Computed free energy barriers Δ$G^‡$ and Gibbs free energy changes ΔG for all proposed reaction pathways

Table S1. Computed free energy barriers Δ$G^‡$ and Gibbs free energy changes ΔG for all proposed reaction pathways at 298 K and 1 bar. R stands for $-CH_2OSO_3^-$ group.

Label	Reaction	Δ$G^‡$ (kJ/mol)		ΔG (kJ/mol)	
**R1a**	RH + •OH → CH_3O• + HSO_4^-	161.7		-50.8	
**R1b**	RH + •OH → R• + H_2O	56.73		-87.9	
**R2**	R•→ HCHO + SO_3•⁻	76.7		5.1	
**R3**	R• + O_2→ ROO•	11.5		-137.6	
**R4 (without 6 water molecules)**	ROO• → HCHO + SO_5•⁻	232.6		58.9	
**R5**	2ROO• → ROOOOR	48.9		-25.6	
**R7**	ROOOOR → RO• + ROOO•	70.1		39.6	
**R8**	RO• + ROOO• → 2RO• + O_2	4.2		-63.1	
**R6a**	2ROO• → ROH + ROOOH	177.0		-269.4	
**R6b**	2ROO• →ROH+ ROO(H)O	128.1		-152.6	
**R9a**	RO• →•CHO + HSO_4^-	27.2	28.6	barrierless	-68.2
**R9b**	RO• →•CHO + HSO_4^-	58.5		-53.1	
**R10**	R• + O_3 → ROOO•	Not found		-301.5	
**R11**	ROOO• →RO• + O_2	3.8		-47.2	

OH oxidation of an isoprene-derived organosulfate

To test whether the non-sulfur-radical mechanism elucidated for methyl sulfate ($CH_3OSO_3^-$) is transferable to atmospherically more relevant, larger organosulfates, we performed a parallel set of calculations on a representative isoprene-derived organosulfate (isoprene-OS, 2-methyl-2,3,4-trihydroxybutyl sulfate, $^-O_3SO–CH_2–CH(OH)–C(CH_3)(OH)–CH_2OH$). Isoprene is by far the largest non-methane biogenic volatile organic compound emitted globally, and its IEPOX-derived organosulfates are among the most abundant OS species detected in ambient fine aerosol. The Lewis structure is shown in **Figure S1**.

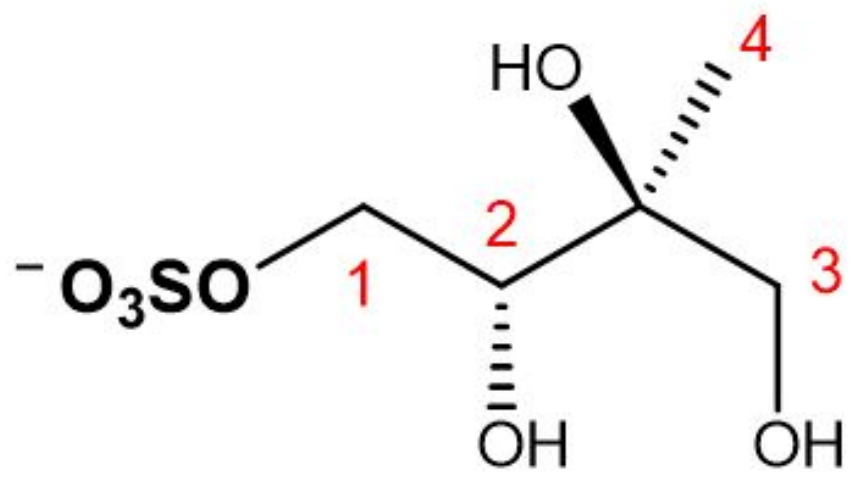

Figure S1. Lewis structure of the isoprene-derived organosulfate (isoprene-OS) used in the generality test, with carbon atoms numbered 1–4. Position 1 (α-carbon to the sulfate ester) is the site investigated in detail in this section.

In this larger molecule, OH abstraction can in principle occur at any of the four C–H-bearing carbons (positions 1–4). To allow for the most direct, like-for-like comparison with methyl sulfate, we focused on OH attack at C1, i.e. the α-carbon directly bonded to the sulfate ester group, which is the position mechanistically analogous to the methyl carbon of $CH_3OSO_3^-$. Attacks at C2–C4 are left for future dedicated studies.

Computational details

All geometry optimizations, frequency analyses, and IRC verifications were performed at the M06-2X-D3(0)/def2-SVP level; single-point energies were refined at the M06-2X-D3(0)/ma-def2-QZVPP level. The SMD implicit solvent model was used together with 6 explicit water molecules to describe the aqueous environment. Solvation free-energy corrections (ΔG_{solv}) were obtained at the M05-2X/6-31G* level, and thermochemistry was evaluated with Shermo at 298 K and 1 bar (RRHO approximation). Conformer pre-screening was again performed with Molclus.

Results: Reaction pathways for OH oxidation of ISO-OS

Figure S2 and **Table S2** summarize the reaction pathways together with the corresponding free-energy barriers $\Delta G^‡$ and reaction free-energy changes ΔG along the same set of elementary steps (**R1b**, **R2**, **R3**, **R5**, **R6a/b/c**, **R7**, **R8**, **R9a**, **R9b**) defined in **Scheme 2** of the main text, plus two additional processes specific to the larger system: (1) A unimolecular H-shift of the C-centered radical R• that competes with **R3**. In contrast to the methyl-sulfate case, where the α-carbon radical $•CH_2OSO_3^-$ has no internal H-donor available for an intramolecular H-shift. The isoprene-OS-derived C1 radical can in principle undergo a 1,n-hydrogen migration from the hydroxyl-bearing C2/C3/C4 backbone, generating α-hydroxy C-radical without invoking O_2. This unimolecular channel is therefore an additional competitor, and (2) **(2)** an alkoxy-radical β-scission step ("newly proposed") that competes with **R9a** from the common reactant **IM6** (RO•**).** Whereas **R9a** cleaves the $C1–OSO_3^-$ bond and releases HSO_4^- via

water-assisted two-step (as in the main text), "newly proposed" cleaves the C1–C2 bond: the unpaired electron migrates from the α-O, through the breaking C1–C2 bond, onto C2, simultaneously converting C1 into a carbonyl while retaining the sulfate ester. The products are a sulfate-bearing formyl sulfate $HC(=O)OSO_3^-$ and a tertiary α-hydroxy C-radical $•C(OH)(CH_3)–CH(OH)–CH_2OH$. Newly proposed is therefore a kinetic competitor of **R9a**, not a sequential step along the same path; it has no analog in methyl sulfate, whose RO• ($•OCH_2OSO_3^-$) lacks any C–C bond suitable for β-scission.

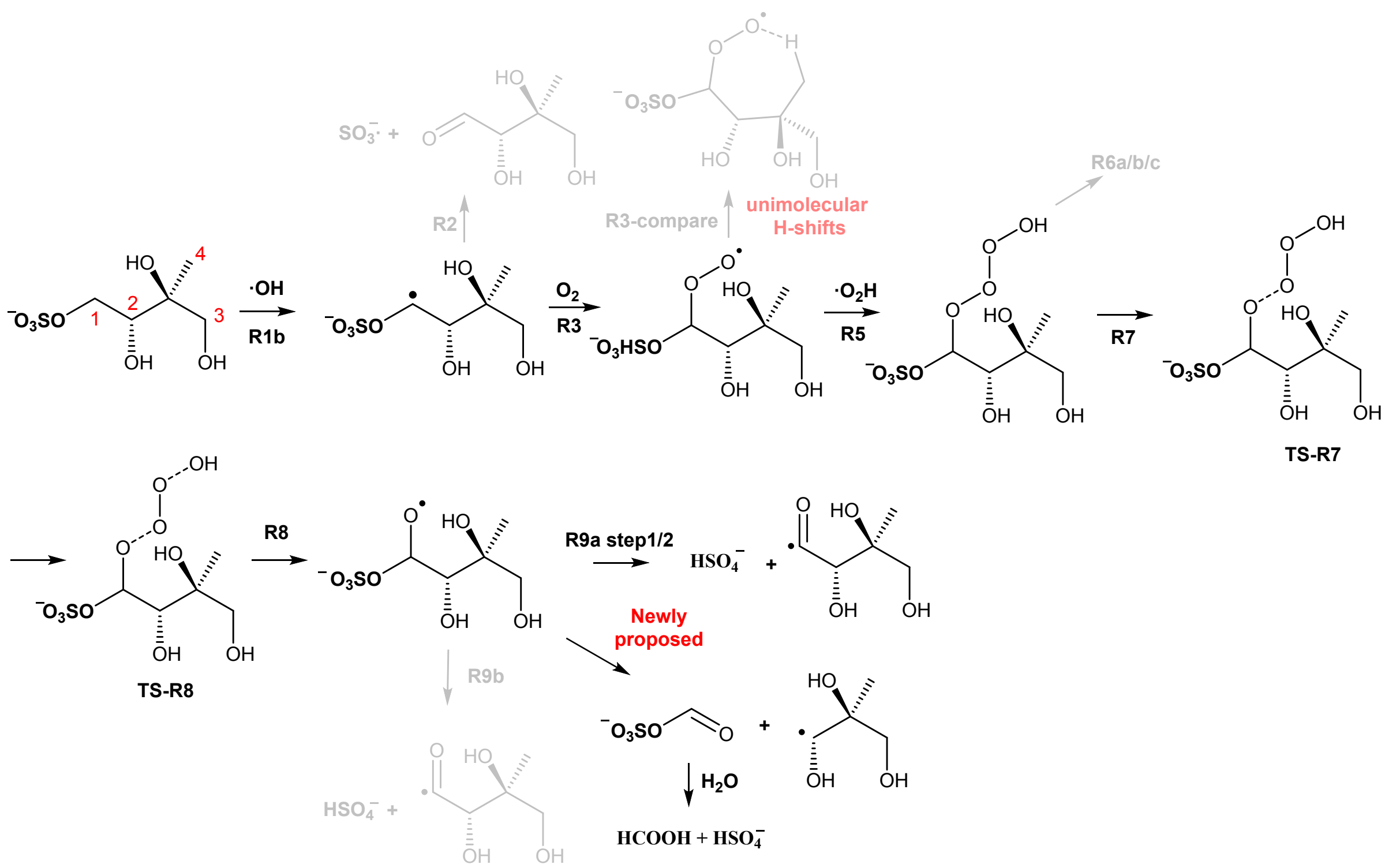

Figure S2. Proposed reaction network for OH-initiated oxidation of the isoprene-derived organosulfate (isoprene-OS) at the C1 α-carbon. Carbon atoms are numbered 1–4 in red, with C1 corresponding to the sulfate-adjacent α-carbon investigated in this work.

Table S2. Computed free energy barriers $\Delta G^{\ddagger}$ and Gibbs free energy changes ΔG for all proposed reaction pathways at 298 K and 1 bar.

<table>
<tr><th>Label</th><th colspan="2">ΔG‡ (kJ/mol)</th><th colspan="2">ΔG (kJ/mol)</th></tr>
<tr><td>R1b</td><td colspan="2">27.35</td><td colspan="2">-92.57</td></tr>
<tr><td>R2</td><td colspan="2">69.07</td><td colspan="2">-14.18</td></tr>
<tr><td>R3</td><td colspan="2">10.16</td><td colspan="2">-117.88</td></tr>
<tr><td>Unimolecular H-shifts</td><td colspan="2">89.31</td><td colspan="2">31.94</td></tr>
<tr><td>R5</td><td colspan="2">40.96</td><td colspan="2">-12.62</td></tr>
<tr><td>R7(OOOH leave first)</td><td colspan="2">56.92</td><td colspan="2">21.77</td></tr>
<tr><td>R8 (OH leave second)</td><td colspan="2">26.67</td><td colspan="2">-16.70</td></tr>
<tr><td>R7(OH leave first)</td><td colspan="2">98.35</td><td colspan="2">-0.25</td></tr>
<tr><td>R8 (OO leave second)</td><td colspan="2">285.64</td><td colspan="2">-256.12</td></tr>
<tr><td>R9b</td><td colspan="2">59.32</td><td colspan="2">-60.58</td></tr>
<tr><td>Newly proposed</td><td colspan="2">-5.1*</td><td colspan="2">-37.92</td></tr>
<tr><td>R9a</td><td>-1.40*</td><td>-7.01</td><td>0.07</td><td>-57.98</td></tr>
<tr><td>R6a</td><td colspan="2">154.02</td><td colspan="2">-299.39</td></tr>
<tr><td>R6b</td><td colspan="2">103.71</td><td colspan="2">-281.30</td></tr>
</table>

*Small negative $\Delta G^{\ddagger}$ values (due to approximations) should be interpreted as effectively barrierless and diffusion-limited.

Choice of cross-reaction partner: HO_2• as a representative aqueous RO_2•

For the bimolecular peroxy-radical step, we used HO_2• as the cross-reaction partner (ROO• + HO_2• → ROOOOH) instead of the symmetric self-reaction (2 ROO• → ROOOOR) considered for methyl sulfate, because HO_2• is by far the most abundant peroxy radical in the aqueous phase and also a consider for the time consuming. The *in situ* self-reaction is still very important and need to be further studied in the future.

A direct mechanistic consequence of using an asymmetric tetroxide ROOOOH (rather than a symmetric ROOOOR) is that the two terminal O–O bonds are no longer equivalent: one points toward the bulky sulfate-bearing OS backbone, the other toward the simple –OOH terminus. The two-step O–O cleavage mechanism (**R7** → **R8**) therefore admits two distinct cleavage orderings depending on which terminal O–O bond breaks first — "OOH-end first" and "OS-end first". Both orderings were located and are reported separately in **Table S2**; they are not kinetically equivalent due to the difference of $\Delta G^{\ddagger}$ and ΔG.

Discussion

Four qualitative conclusions emerge that are robust to the change in optimization basis set, since they concern the ranking and accessibility of pathways rather than absolute barrier heights.

(1) The α-carbon entry channel and O_2-addition gateway are preserved. OH attack at C1 (**R1b**, $\Delta G^{\ddagger} \approx 27$ kJ/mol, $\Delta G \approx -93$ kJ/mol) gives a C-centered radical R• whose subsequent fate is again dominated by O_2 addition: **R3** ($\Delta G^{\ddagger} \approx 10$ kJ/mol) is more than 50 kJ/mol lower in barrier than the unimolecular fragmentation **R2** to SO_3•⁻ ($\Delta G^{\ddagger} \approx 69$ kJ/mol) and ≈ 80 kJ/mol lower than the new unimolecular H-shift channel that competes with **R3** ($\Delta G^{\ddagger} \approx 89$ kJ/mol, $\Delta G \approx +32$ kJ/mol). The intramolecular 1,n-H shift, despite being geometrically possible in the OH-rich isoprene-OS scaffold (and absent in methyl sulfate), is therefore kinetically negligible at the α-position under aqueous conditions,

and the system is funneled into peroxy-radical chemistry through the same gate as for methyl sulfate.

(2) Russell-type channels remain kinetically inaccessible. In the asymmetric ROOOOH tetroxide, the four- and five-membered Russell-type variants **R6a** and **R6b** retain prohibitive barriers (154.0 and 103.7 kJ/mol, respectively) despite very large thermodynamic driving forces ($\Delta G \approx -280$ to -300 kJ/mol), and the standard six-membered Russell TS (**R6c**) could not be located. Therefore, the steric/electrostatic argument for methyl sulfates, that the large sulfate ester group is not conducive to the formation of a tight ring transition state, also applies to substituted alkyl OSs.

(3) The asymmetric ROOOOH cleaves preferentially with the OOH end leaving first. Of the two possible decomposition orderings of the asymmetric tetroxide, the "•OOH-first" ordering (**R7**: 56.92 kJ/mol → **R8**: 26.67 kJ/mol) is dramatically more accessible than the "•OS-first" ordering (**R7**: 98.35 kJ/mol → **R8**: 285.64 kJ/mol). This selectivity is intuitive: removing the smaller, less sterically encumbered HOO terminus first avoids forming a high-energy caged pair in which the bulky sulfate-bearing fragment is stranded as the reactive intermediate. Crucially, both orderings, when they do proceed, converge on the same alkoxy-sulfate radical (**IM6** analog) that is the gateway to inorganic sulfate. Therefore, the conclusion drawn for methyl sulfate, namely that the RO_2–RO_2 chemical reaction of OS-containing peroxy radicals is a conversion to alkoxy sulfate radicals rather than to Russell products, is strengthened rather than weakened when considering the ROO• + HO_2• cross-reaction, which is more consistent with atmospheric realities.

(4) A new C–C β-scission channel competes with **R9a** from the alkoxy-sulfate radical, but does not open any sulfate-retaining escape route. From the common alkoxy-sulfate radical **IM6** (RO•), two submerged-barrier elementary steps are available in isoprene-OS that have no analog in methyl sulfate's **R9a-**only branching: the **R9a** (C1–OSO_3^- cleavage, $\Delta G^‡ = -1.40$ / -7.01 kJ/mol, $\Delta G = +0.07$ / -57.98 kJ/mol; releases HSO_4^-) and a new β-scission step ("newly proposed"; C1–C2 cleavage, $\Delta G‡ = -5.11$ kJ/mol, $\Delta G = -37.92$ kJ/mol; the radical relocates from α-O onto C2, leaving a formyl sulfate $HC(=O)OSO_3^-$ and an α-hydroxy tertiary C-radical). The two channels are kinetically competitive (both submerged by a few kJ/mol below the separated reactant asymptote in the explicit-water cluster), but **R9a** is thermodynamically favored by ≈ 20 kJ/mol over the β-scission channel, so on long time scales the partitioning still leans toward direct HSO_4^- release. More importantly, even the "newly proposed" branch does not constitute an escape route that preserves a stable organic sulfate product: its product, formyl sulfate $HC(=O)OSO_3^-$, is itself an acyl-sulfate ester that is an acyl sulfate ester to be hydrolytically and homolytically in aqueous media, and is expected to release HSO_4^-. Thus, both competing channels from **IM6**, directly (**R9a**) or via one additional step (newly proposed → formyl sulfate → HSO_4^-) terminate at inorganic sulfate. This branching is unavailable to methyl sulfate (whose RO• has no C–C bond), and its presence in isoprene-OS therefore introduces additional carbon-skeleton fragmentation but no new sulfate-retaining product channel. The central conclusion of the text is that alkoxy sulfate radicals cannot survive in hydrogen-bonded aqueous solutions and will collapse into HSO_4^-. Therefore, this conclusion is strengthened rather than weakened under larger OS conditions.

Caveats and outlook.

Because the optimization basis set used here for ISO-OS, def2-SVP, differs from that used for the methyl sulfate production calculations in the main text, def2-TZVP, we refrain from making numerical, kJ mol−1-level comparisons of individual barriers

between the two molecules. The present calculations are intended as an internally consistent qualitative test of the C1, α-sulfate oxidation channel.

The key result is that the sulfate-adjacent oxidation motif remains viable for the isoprene-derived organosulfate: OH abstraction at C1, O_2 addition, peroxy-radical conversion to an alkoxy organosulfate radical, and R9a-type sulfate release can all be located on the Iso-OS potential energy surface. At the same time, the larger carbon framework introduces an additional low-energy competing C1−C2 bond-cleavage/radical-relocation pathway, newly proposed. This pathway is absent in methyl sulfate and illustrates that larger organosulfates can undergo skeletal fragmentation or radical relocation processes that are not captured by the methyl sulfate model.

Therefore, the ISO-OS calculations support the qualitative relevance of the α-sulfate oxidation and sulfate-release motif, but they do not imply that methyl sulfate is a complete mechanistic surrogate for all organosulfates. The present study addresses only OH attack at C1. H-abstraction at C2–C4 may open additional channels, including intramolecular H-shifts, autoxidation-like pathways, β-scission, functionalization, or fragmentation with retention of the sulfate ester. These site-specific pathways require dedicated future calculations, ideally at the same optimization level as the methyl sulfate production calculations.

Spin density analyzation for R1a and R1b

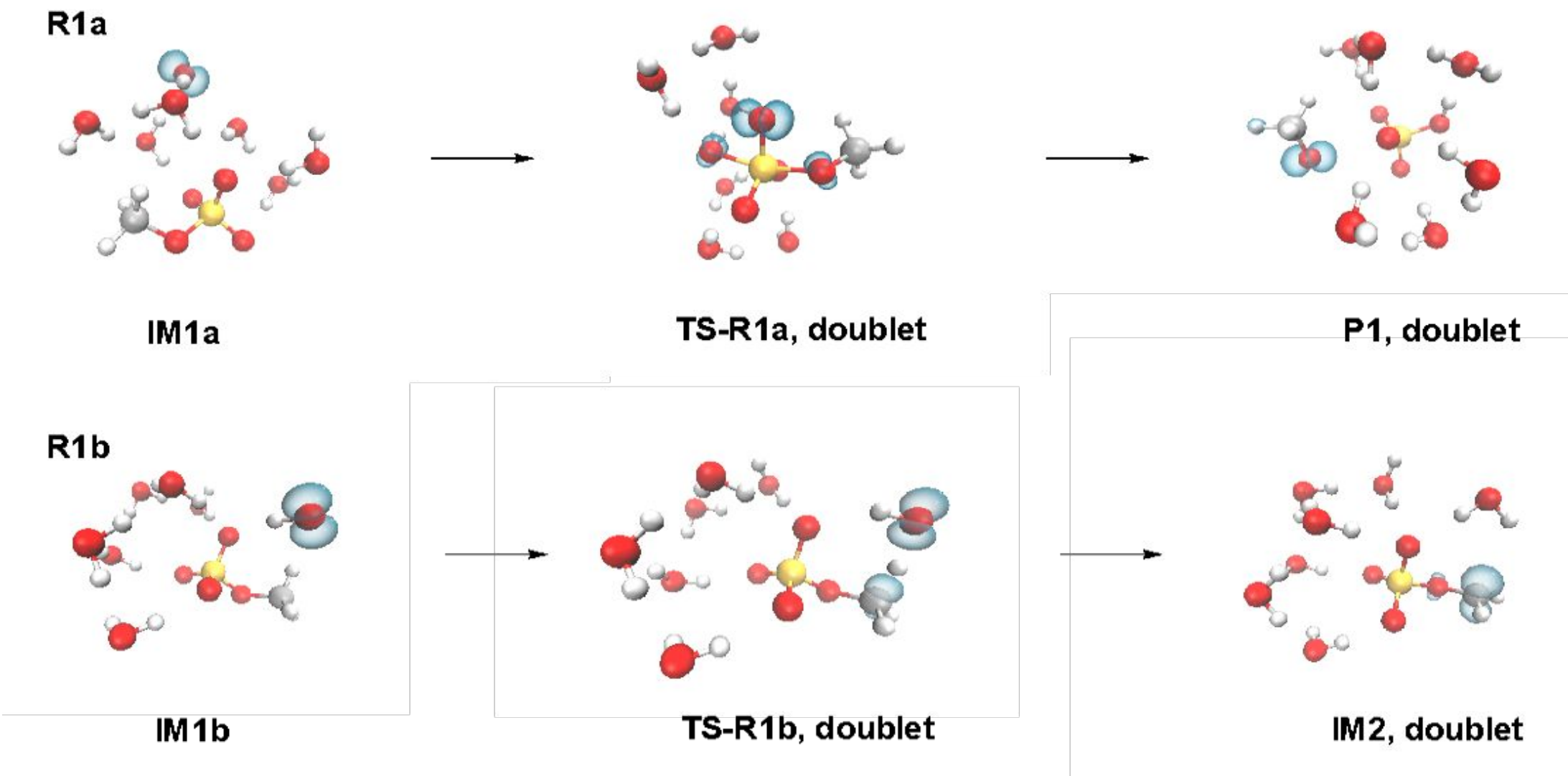

Figure S3. Spin density isosurfaces (green: α-spin, cyan: β-spin) for the **R1a** and **R1b** reactions. The sulfur, oxygen, carbon, and hydrogen atoms are shown in yellow, red, grey, and white, respectively. In **TS-R1a**, spin density localizes on the departing methoxy fragment as the S–OCH_3 bond cleaves, consistent with the high barrier for direct attack at the sulfur center. In **TS-R1b**, hydrogen abstraction transfers spin density to the α-carbon, consistent with formation of the carbon-centered radical **IM2** as the favored initial oxidation pathway.

Pseudo-first-order rate constants for the competition between R2, R3, and R10
Because **R3** and **R10** are bimolecular (second-order, s^{-1} M^{-1}), whereas **R2** is unimolecular (first-order, s^{-1}), the rate constants are not directly comparable. However, since dissolved O_2 and O_3 are in vast excess relative to the radical $\bullet CH_2OSO_3^-$ (IM2) in the atmosphere, the bimolecular rate constants can therefore be recast as pseudo-first-order, $k_i' = k[X(\mathrm{aq})]$, where $[X(\mathrm{aq})]$ is the dissolved concentration of O_2 or O_3 that can be estimated by the Henry's law.

Using the Henry's law constants at 298 K compiled by Sander[1], $H_{O_2} = 1.3 \times 10^{-3}$ M atm^{-1} and $H_{O_3} = 1.03 \times 10^{-2}$ M atm^{-1}, together with the partial pressures $p_{O_2} = 0.21$ atm and $p_{O_3} = 3.0 \times 10^{-8}$ atm (at 30 ppb), the aqueous concentrations are:

$$[O_2(aq)] = H_{O_2}\, p_{O_2} = 2.73 \times 10^{-4}\ \mathrm{M}$$
$$[O_3(aq)] = H_{O_3}\, p_{O_3} = 3.09 \times 10^{-10}\ \mathrm{M}$$

Combining these concentrations with our calculated rate constants (**R3**: $k_{\mathrm{total}} = 8.01 \times 10^9$ s^{-1} M^{-1}, **R10**: $k_{\mathrm{total}} = 7.65 \times 10^9$ s^{-1} M^{-1}, **R2**: $k_{\mathrm{total}} = 2.43 \times 10^{-1}$ s^{-1} M^{-1}) yields:

Table S3. Pseudo-first-order rate constants for the competing **R2**, **R3**, and **R10** pathways under atmospherically relevant dissolved O_2 and O_3 concentrations.

Pathway	Reaction	k_i' (s^{-1})	Relative k
**R3**	$\bullet CH_2OSO_3^- + O_2 \rightarrow \bullet OOCH_2OSO_3^-$	2.19×10^{6}	1
**R10**	$\bullet CH_2OSO_3^- + O_3 \rightarrow \bullet OOOCH_2OSO_3^-$	2.36	1.1×10^{-6}
**R2**	$\bullet CH_2OSO_3^- \rightarrow HCHO + SO_3\bullet^-$	2.43×10^{-1}	1.1×10^{-7}

Therefore, **R3** dominates at these concentrations of O_2 and O_3.

Post-R6b decomposition of the Hydrotrioxide

To assess whether the hydrotrioxide-functionalized organosulfate $^{-}OOOCH_2OSO_3^{-}$ formed in the five-membered Russell-type pathway (**R6b**) could serve as a kinetic precursor to alcohol formation, we computed its subsequent decomposition within the same calculation level and hybrid (6 H_2O + SMD) framework as in the main text. The hydrotrioxide undergoes homolytic O–O cleavage:

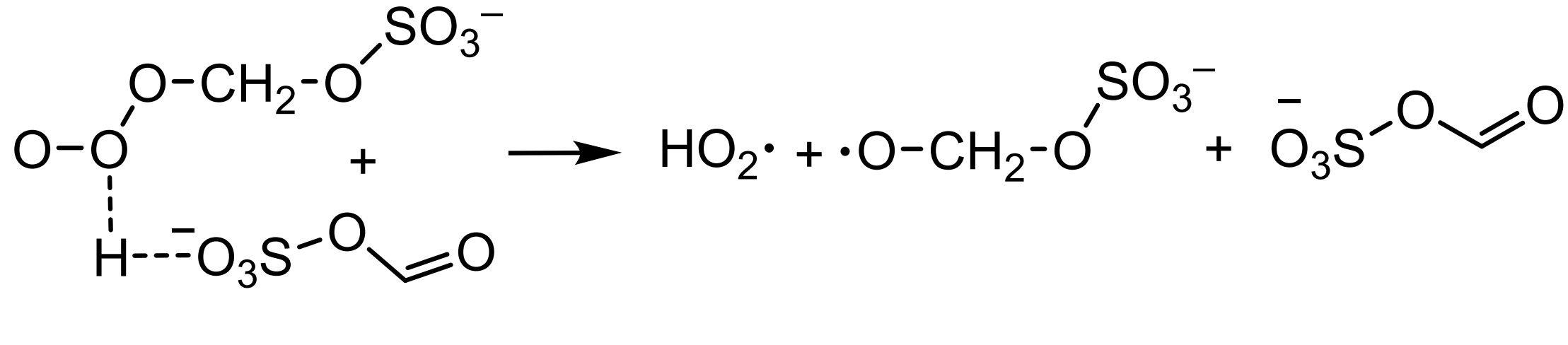

P5

Figure S4. Homolytic O–O cleavage of the hydrotrioxide-functionalized organosulfate (**P5**) yielding the alkoxy sulfate radical (IM6, $\bullet OCH_2OSO_3^{-}$), the hydroperoxyl radical ($HO_2\bullet$), and the formate–sulfate adduct ($^{-}O_3SOCHO$).

with $\Delta G^{\ddagger}$ = 18.90 kJ/mol and ΔG = –57.04 kJ/mol at 298 K. The TS was confirmed by a single imaginary frequency along the breaking O–O coordinate and verified by IRC. This step generates **IM6**, which is the same as the alkoxy sulfate radical directly generated in the main reaction pathway **R7→R8**, and **no alcohol product is generated**. Because the overall flux through the Russell-type channel is still gated by the **R6a**/**R6b** TS itself ($\Delta G^{\ddagger}$>128 kJ/mol), it remains ~60 kJ/mol higher than the rate-limiting R7 ($\Delta G^{\ddagger}$ = 70.1 kJ/mol) and is therefore kinetically uncompetitive. This is consistent with the absence of alcohol- or carbonyl-functionalized signals in our ESI-MS spectra.

Environmental relevance of IM3 self-reaction and cross-reactions with atmospherically relevant peroxy radicals

Environmental relevance of IM3 + IM3 in aqueous aerosols

Ambient aqueous aerosols are chemically complex and rarely contain a high concentration of any single gas-phase-derived RO_2. However, the situation for **IM3** is fundamentally different from that of typical gas-phase RO_2: **IM3** is generated in situ within the aerosol particle. Unlike volatile RO_2 species that must partition from the gas phase, **IM3** is produced directly within the condensed phase by aqueous-phase OH-initiated H-abstraction from the parent organosulfate, followed by O_2 addition (**R1**–**R3** in **Scheme 2**). Its local concentration is therefore controlled by the abundance of the parent organosulfate and aqueous OH flux, not by gas-phase partitioning. As we mentioned in our introduction, atmospheric organosulfates can reach mass loadings of up to several μg m^{-3} in SOA-rich environments and constitute up to ~30% of organic aerosol mass, so the steady-state **IM3** concentration in the particle phase can be appreciably higher than that of gas-phase-partitioned RO_2. It's reasonable to use **IM3** + **IM3** reaction as prototype reaction.

Additional calculations: IM3 + CH_3O_2• and IM3 + HO_2•

Among gas-phase RO_2 partitioned into the aqueous phase, the dominant species are HO_2• and CH_3O_2•, both of which we have now examined. Methyl peroxy radicals (CH_3O_2•) are the most abundant organic peroxy radical species and are critical intermediates in rapid chemical cycling at the heart of tropospheric oxidation,[2] accounting for 83% of total tropospheric RO_2 in global model studies, followed by isoprene peroxy radicals.[3] HO_2• is the most abundant peroxy radical overall and, owing to its high effective Henry's law constant (3900 M atm^{-1} at 298K for HO_2•),[4] partitions strongly into aqueous aerosol. We have therefore selected CH_3O_2• and HO_2• as the two important cross-partners for **IM3** in aqueous aerosols.

Using the same level of theory as in the main text, we computed the three analogous steps of the asymmetric decomposition pathway (analogous to **R5→R7→R8**) and Russell-type (analogous to **R6a/b/c**) pathways for **IM3** with the two mixed tetroxides. All energies are $\Delta G^{‡}/\Delta G$ in kJ/mol at 298 K and reported relative to the corresponding pre-reaction complex.

Unlike the symmetric **IM3** + **IM3** tetroxide, both $ROOOOCH_3$ and ROOOOH are structurally asymmetric. We therefore explicitly explored both directional channels for each elementary step:

Asymmetric two-step cleavage **(R7/R8-type).** The first O–O cleavage can occur at the R-side or the CH_3/H-side.

Russell-type H-transfer **(R6-type).** H abstraction can proceed from the organosulfate α-CH_2 (**H1**) or from the partner CH_3 / HO_2 moiety (**H2**).

Table S4. Computed free energy barriers $\Delta G^{‡}$ and reaction free energies ΔG for **IM3** cross-reactions with CH_3O_2• and HO_2• at 298 K and 1 bar. R stands for $-CH_2OSO_3^{-}$ group.

Reaction	$\Delta G^{\ddagger}$ (kJ/mol)	ΔG (kJ/mol)
**IM3 + CH_3O_2• → ROOOOCH$_3$**	39.73	-27.26
**IM3 + HO_2• → ROOOOH**	70.06	20.07
**ROOOOCH$_3$→ ROOO• + •OCH$_3$**	84.37	59.79
**ROOO• + •OCH$_3$→ RO•+ O_2 + •OCH$_3$**	6.05	-60.86
**ROOOOCH$_3$→ RO• + •OOOCH$_3$**	105.64	37.28
**RO• + •OOOCH$_3$→ RO•+ O_2 + •OCH$_3$**	11.63	-49.92
**ROOOOH→ ROOO• + •OH**	127.72	77.35
**ROOO• + •OH→ RO•+ O_2 + •OH**	5.19	-67.28
**ROOOOH→ RO• + •OOOH**	56.92	21.77
**RO• + •OOOH→ RO•+ O_2 + •OH**	32.98	-12.10
**S1-R6a1**	176.57	-260.35
**S1-R6b1**	115.07	-136.57
**S2-R6a1**	190.88	-286.17
**S2-R6b1**	122.27	-143.83
**S2-R6c1**	205.42	-280.10

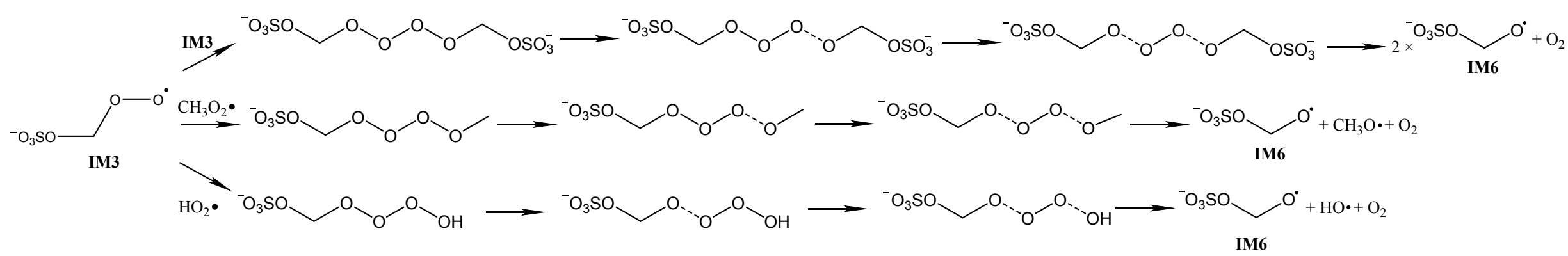

Figure S5. Schematic reaction pathways for the stepwise tetroxide-mediated decomposition of three RO_2–RO_2 systems: **IM3** + **IM3** (top, self-reaction), **IM3** + CH_3O_2• (middle, cross-reaction **S1**), and **IM3** + HO_2• (bottom, cross-reaction **S2**). For each system, the reaction proceeds through (i) formation of the (mixed) tetroxide intermediate ROOOOR′, (ii) the first O–O bond cleavage along the kinetically preferred direction, and (iii) the second O–O cleavage that releases $^3O_2$. All three pathways converge on the same alkoxy sulfate radical **IM6** (•$OCH_2OSO_3^-$), accompanied by the partner alkoxy radical (a second **IM6** for the self-reaction; •OCH_3 for CH_3O_2•; •OH for HO_2•), demonstrating that the $-OSO_3^-$ terminus universally directs the decomposition outcome regardless of partner identity.

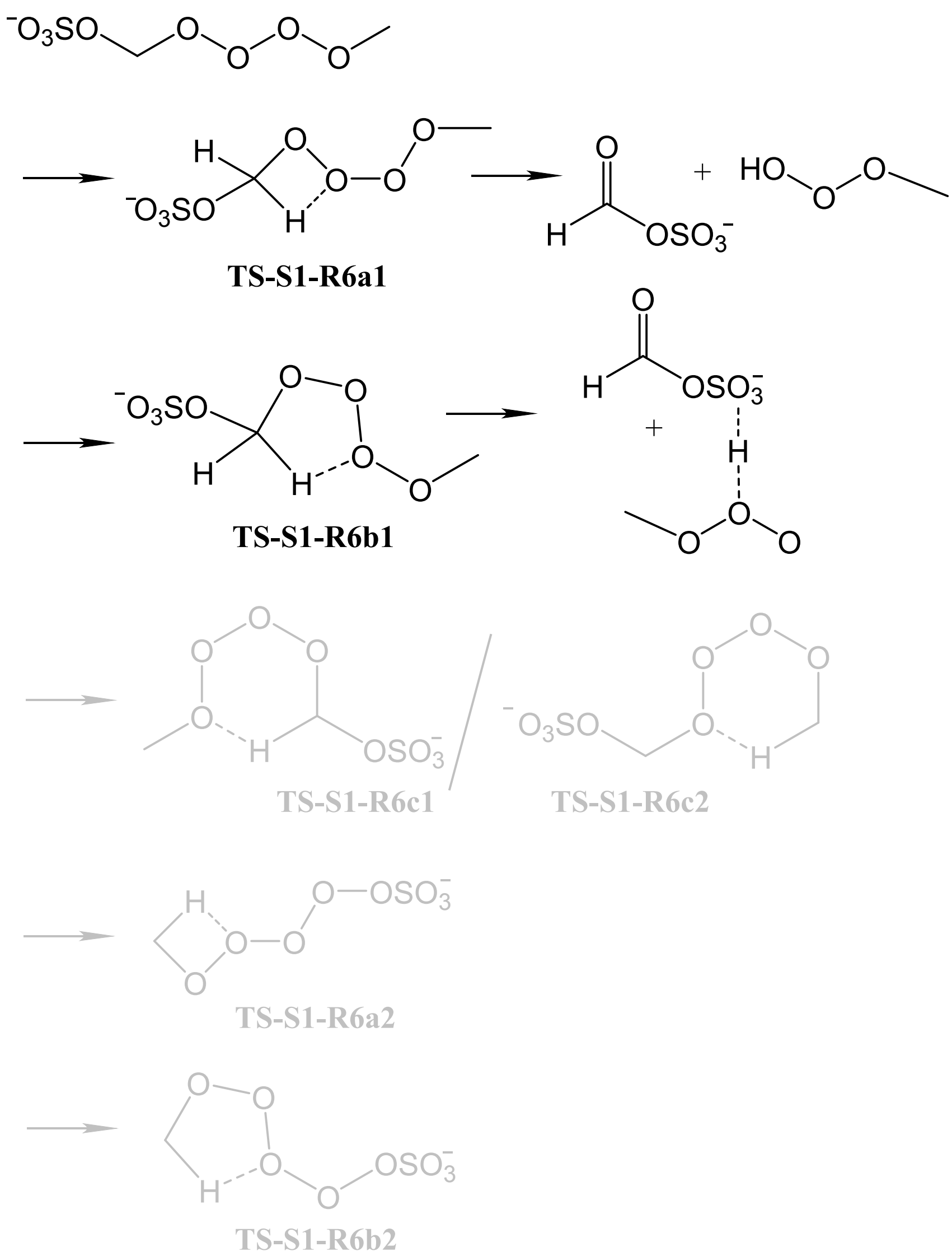

Figure S6. TS structures for the Russell-type pathways of the **IM3** + CH_3O_2• mixed tetroxide ($ROOOOCH_3$). Two distinct hydrogen sources were considered: H1 = α-CH of the organosulfate moiety, and H2 = methyl hydrogen of the CH_3O_2 partner. **TS-S1-R6a1** (four-membered ring, H1 source) and **TS-S1-R6b1** (five-membered ring with a stabilizing intramolecular H-bond, H1 source) lead to the formyl sulfate ($HC(O)OSO_3^-$) + CH_3OOOH product channel. The grayed structures (**TS-S1-R6a2**, **TS-S1-R6b2**, **TS-S1-R6c1**, **TS-S1-R6c2**) denote alternative ring topologies and the H2-source channels, which could not be located. Solid arrows indicate productive (located) TSs; gray arrows indicate disfavored/unlocated channels.

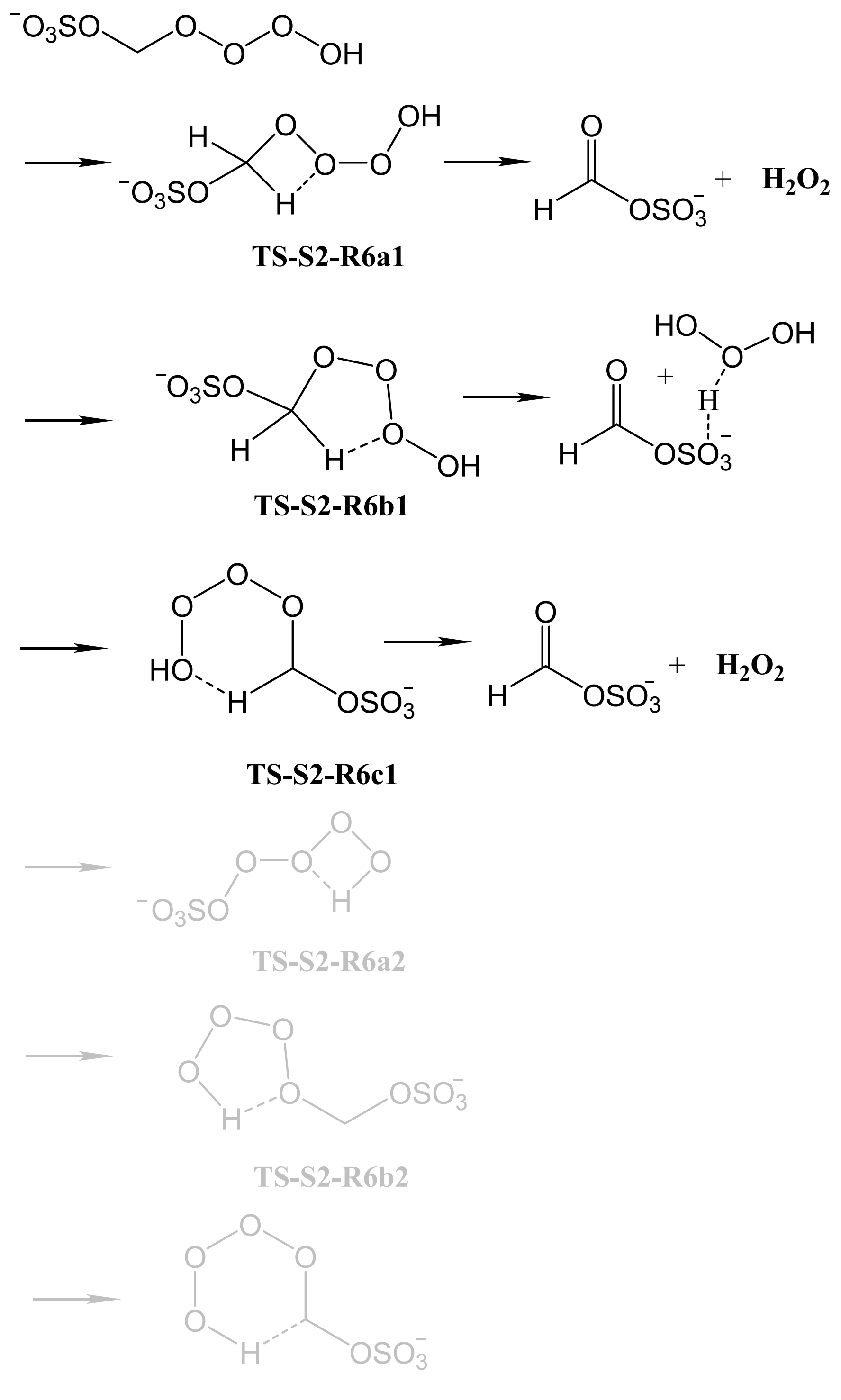

Figure S7. TS structures for the Russell-type pathways of the **IM3** + HO_2• mixed tetroxide (ROOOOH). Two distinct hydrogen sources were considered: H1 = α-CH of the organosulfate moiety, and H2 = hydroperoxyl hydrogen of HO_2•. **TS-S2-R6a1** (four-membered ring, H1 source) and **TS-S2-R6b1** (five-membered ring with a stabilizing intramolecular H-bond, H1 source) lead to the formyl sulfate ($HC(O)OSO_3^{-}$) + H_2O_2 product channel via abstraction of the organosulfate α-H. **TS-S2-R6c1** (six-membered ring, H2 source) corresponds to the alternative channel in which the HO_2• hydrogen is transferred. The grayed structures (**TS-S2-R6a2**, **TS-S2-R6b2**, and the alternative **TS-S2-R6c1** topology) denote alternative ring conformations that could not

be located. Solid arrows indicate productive (located) TSs; gray arrows indicate disfavored/unlocated channels.

Three key observations:

(a) Universality of the mechanism/formation of **IM6**. In all three cases (self- and cross-), the asymmetric two-step pathway is energetically connected, and crucially, decomposition of the mixed tetroxide always yields the target alkoxy sulfate radical **IM6** (•$OCH_2OSO_3^-$) together with $^3O_2$ and the partner alkoxy radical (CH_3O• or •OH for the CH_3O_2• and HO_2• partners, respectively). This directly addresses the reviewer's question about IM6 formation and supports our statement that the unique structural features of the organosulfate-bearing terminus dictate the *identity* of the surviving radical (**IM6**), independent of the partner.

(b) **IM3** + **CH_3O_2•** is kinetically comparable to but slightly slower than **IM3** + **IM3**. The mixed tetroxide forms more readily ($\Delta G^‡$ lower by ~9 kJ/mol) due to the lower steric demand of CH_3O_2•, but its rate-limiting first O–O cleavage is ~14 kJ/mol higher (84.37 kJ/mol vs. 70.13 kJ/mol), because only one $-OSO_3^-$ group is present in the singlet caged biradical to electrostatically stabilize the incipient alkoxy/peroxyl pair. Net effect: the cross-reaction proceeds at a comparable but somewhat slower rate, and gives the same IM6 product. Nonetheless, the partitioning of CH_3O_2• into condensed-phase OS-aerosols is limited by its low effective Henry's-law constant ($H_{eff} \approx 6$ M atm^{-1}),[5] so its quantitative contribution to **IM3** chemistry in the aqueous phase is minor. Therefore, in environments where CH_3O_2• partitions into the aerosol, this channel constitutes an additional, parallel route to IM6, reinforcing rather than competing with the proposed mechanism.

(c) **IM3** + HO_2• is mechanistically analogous to **IM3** + **IM3** and provides a viable parallel channel. Mixed tetroxide formation is feasible ($\Delta G^‡$ = 70.06 kJ/mol), and its stepwise decomposition via the inner O–O cleavage ($\Delta G^‡$ = 56.92 kJ/mol) continues to produce the same alkoxy sulfate radical **IM6**. Although this rate-limiting barrier is comparable to **IM3** + **IM3** ($\Delta G^‡$ = 70.13 kJ/mol) and even lower than **IM3** + CH_3O_2• (84.37 kJ/mol), thermodynamically the **IM3** + **IM3** pathway retains a decisive advantage: its tetroxide formation is exergonic (ΔG = −25.64 kJ/mol) and the first O–O cleavage is only mildly endergonic (ΔG = +39.55 kJ/mol), whereas the corresponding steps for **IM3** + HO_2• are endergonic by +20.07 and +21.77 kJ/mol, respectively. The channel of **IM3** + HO_2• (ROOO• + •OH, $\Delta G^‡$ = 127.72 kJ/mol) is kinetically inaccessible. Russell-type barriers (≥115 kJ/mol) are likewise non-competitive. Thus, replacing one **IM3** by HO_2• does not change the identity of the key sulfate-forming intermediate **IM6**, Given the abundance of HO_2• in water, a feasible parallel pathway is provided, while **IM3** + **IM3** remains the thermodynamically dominant pathway.

Taken together, these results support the hypothesis in our manuscript that the unique structural features of organosulfates dictate the decomposition outcome. Specifically, the symmetric **IM3** + **IM3** tetroxide is uniquely stabilized in its singlet caged-biradical decomposition state by the *bilateral* anionic $-OSO_3^-$ groups, which provide electrostatic stabilization of the alkoxy radical pair through space and lower the asymmetric two-step cleavage barrier. Replacement of one $-OSO_3^-$ by either −H (HO_2•)

or $-CH_3$ (CH_3O_2•) converges to the same alkoxy radical product, supporting the generality of the proposed mechanism.

Alkoxy sulfate radical (•$OCH_2OSO_3^-$): Fragmentation via minor R9b and R9c pathways

In the main text, we introduced the water-assisted **R9a** pathway, identified as the dominant mechanism for the decomposition of the alkoxy sulfate radical **(IM6)**. In addition, we examined the minor intramolecular pathways **R9b** and **R9c**. Notably, under conditions with fewer explicit water molecules, the **R9a** pathway proceeds through a more compact transition state, in which only a single water molecule mediates the proton transfer. We name it as **R9a2** and as illustrated in the **Figure S8**.

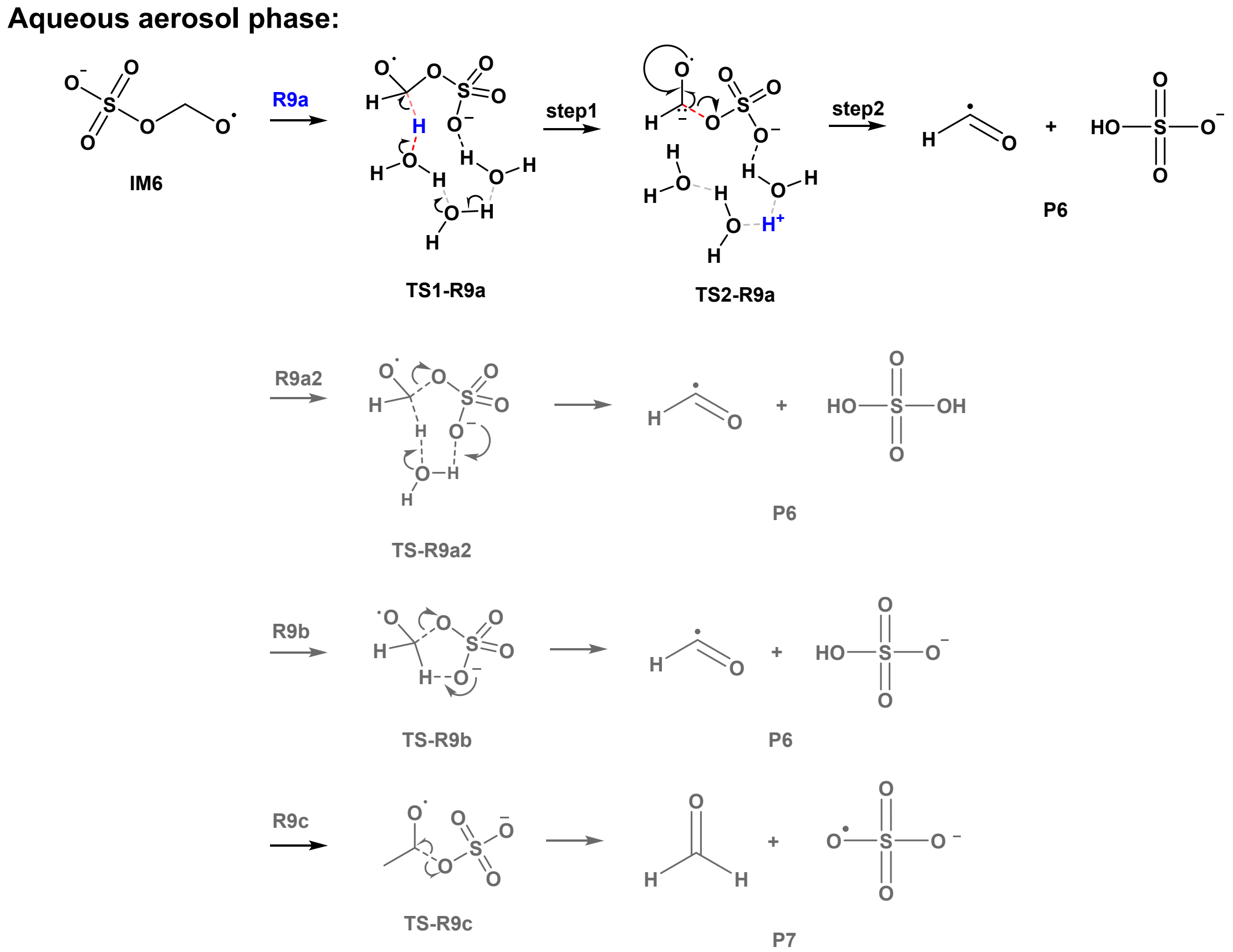

Figure S8. Proposed reaction mechanisms for the **R9a, R9a2, R9b,** and **R9c** pathways leading to carbon-centered radical formation. The **R9a**, **R9a2**, and **R9b** pathways share a similar mechanistic framework and constitute the major reaction channels. The primary difference among them lies in the extent of involvement of explicit water molecules in the transition state region. **R9a2** becomes more favorable when fewer water molecules are present. **R9b** is accessible across a wider hydration range (0–6 explicit H_2O molecules) but with a higher barrier. In contrast, **R9c** is a minor pathway, only observed under limited solvation configurations. Further thermodynamic and kinetic details are provided in the following sections.

In the intramolecular route (**R9b**), a proton from the α-carbon is transferred to sulfate oxygen through a five-membered cyclic transition state (**TS-R9b**). Simultaneously, the C-OSO_3^- bond undergoes heterolytic cleavage, with both bonding electrons transferred to the sulfate group, yielding an HSO_4^- and a short-lived intermediate with a sp^2 carbon, which rapidly yields the •CHO radical. The optimized structure of **TS-R9b**[6] exhibits several key geometric features consistent with the proton-transfer bond cleavage. The C-OSO_3^- bond elongates significantly from **1.42 Å** in the reactant complex to **1.53 Å** in the TS, with the α-proton transferred via a five-membered ring (C···H = 1.44 Å, O···H

= 1.22 Å). The reaction proceeds with $\Delta G^{\ddagger}$ = 58.53 kJ/mol and a favorable ΔG = -53.09 kJ/mol.

To further assess the impact of explicit solvent structure, we systematically varied the number of explicit water molecules from **0** to **6** explicit water molecules with the SMD implicit water model. The intramolecular transition state **TS-R9b**, featuring a compact five-membered ring, remains consistently accessible across 0 to 6 explicit water conditions, with negligible variation in $\Delta G^{\ddagger}$ (about 60 kJ/mol) and ΔG (about -30 kJ/mol). This stability is attributed to self-contained geometry and solvent-independent proton transfer. This indicates that **TS-R9b** is largely insensitive to the number of explicit water molecules. However, as water molecules begin to form hydrogen bonds with the alkoxy radical or sulfate group, they may disrupt the C-H···OSO_3^- alignment necessary for **TS-R9b** and instead participate in the proton relay. Under such conditions, **TS-R9a2** becomes accessible, with $\Delta G^{\ddagger}$ values varying between 40-50 kJ/mol depending on the local solvent configuration. The presence of water molecules as proton relays not only lowers the barrier but also reduces the reaction free energy by stabilizing both reactant and product states via hydrogen-bond networks.

As the number of explicit waters increases, **IM6** forms additional hydrogen bonds that facilitate stepwise proton transfer. A clear mechanistic shift is observed from **TS-R9a2** to **TS1-R9a** and **TS2-R9a**. Our observations collectively indicate that the availability and arrangement of water molecules play a critical role in determining the reaction mechanism. In sparsely hydrated environments, the system tends to favor the geometrically compact **TS-R9a2**, in which proton transfer occurs via a single water molecule. As the hydration level increases, the energetic advantage and geometric flexibility of the proton relay mechanism in **TS1-R9a** and **TS2-R9a** become increasingly pronounced. The reduction in the second-step barrier with increasing hydration highlights the fundamental role of solvent structure in enabling low-energy pathways for proton transfer reactions in aqueous media.

Table S5. Computed $\Delta G^{\ddagger}$ and ΔG for the proposed proton transfer and C-OSO_3^- bond cleavage steps along the **R9b** and **R9a2** reaction pathways at 298 K and 1 bar in aqueous phase. "*n*" stands for the number of explicit water molecules in the calculations.

•OCH_2-O-SO_3^- + *n* H_2O → **TS-R9b^n** → •CHO + HSO_4^- + *n* H_2O, where *n* = 1-6		
n	$\Delta G^{\ddagger}$ (kJ/mol)	ΔG (kJ/mol)
0	58.6	-29.4
1	59.8	-27.6
2	60.3	-41.0
3	63.0	-26.8
4	61.8	-30.3
5	58.2	-35.0
6	60.5	-25.7
•OCH_2-O-SO_3^- + *n* H_2O → **TS-R9a2^n** → •CHO + HSO_4^- + *n* H_2O, where *n* = 1-5		
n	$\Delta G^{\ddagger}$ (kJ/mol)	ΔG (kJ/mol)
1	41.6	-40.0
2	36.3	-45.3

3	43.0	-40.3
4	59.4	-37.61
5	40.8	-36.20
6	NA	NA

As shown in **Figure S8**, the **R9c** pathway entails homolytic cleavage of the C-OSO_3^- bond, with one electron remaining on each fragment. This results in a diradical intermediate (•CH_2O•) and a SO_4•⁻. The •CH_2O• species undergoes rapid internal spin pairing to yield HCHO as one of the final products. The viability of the **R9c** pathway was found to be highly sensitive to the solvation environment. Among all configurations sampled, transition states for **R9c** (**TS-R9c**) could only be located when using the SMD implicit solvent model with 1–4 explicit water molecules. Detailed free energy profiles for the **R9c** pathways under these solvation conditions are shown in **Table S6**. In these cases, the $\Delta G^{\ddagger}$ is consistently high, around 80 kJ/mol, and the ΔG is relatively unfavorable, ranging from +10-20 kJ/mol. Two factors likely account for this: (1) The requirement of a specific hydrogen-bonding motif involving both the alkoxy and sulfate oxygen to stabilize **TS-R9c** and suppress other TS. (2) The product of the **R9c** pathway, SO_4•⁻, is highly unstable in aqueous environments. Due to its high energy and strong reactivity, it is likely to undergo rapid secondary reactions with surrounding water molecules, converting to more stable species such as HSO_4^-. This instability further disfavors the **R9c** channel under aqueous conditions.

Table S6. Computed $\Delta G^{\ddagger}$ and ΔG for the proposed proton transfer and C-OSO_3^- bond cleavage steps along the **R9c** reaction pathways at 298 K and 1 bar in aqueous phase. "*n*" stands for the number of explicit water molecules in the calculation.

•OCH_2-O-SO_3^- + n H_2O → **TS-R9c^n** → HCHO + SO_4•⁻ + n H_2O, where n = 1-6		
n	$\Delta G^{\ddagger}$ (kJ/mol)	ΔG (kJ/mol)
0	57.9	-0.4
1	67.1	8.8
2	82.6	26.2
3	73.0	73.0
4	81.4	21.3
5	NA	NA
6	NA	NA

Interestingly, a unique hydrogen-bonding pattern was observed in the **TS-R9c**. In this configuration, the explicit water molecule simultaneously forms hydrogen bonds with both the alkoxy oxygen and sulfate oxygen (each at 1.99 Å), stabilizing a distinct geometry that suppresses ring closure and instead promotes homolytic cleavage. This unique local hydrogen-bonding arrangement may help to stabilize both pre-reaction complex and the TS in **R9c** pathway, and enables **TS-R9c** formation. The computed $\Delta G^{\ddagger}$ and ΔG are shown in **Figure S9**.

However, when additional explicit water molecules were included (5-6 explicit water molecules), this specific hydrogen bonding configuration was no longer observed. This is likely due to the formation of extended hydrogen-bonding networks among water molecules, which disrupt the delicate dual-hydrogen-bonding motif necessary for **TS-R9c**. Moreover, the increased explicit water molecules facilitate **R9a**, where proton transfers are mediated through water wires.

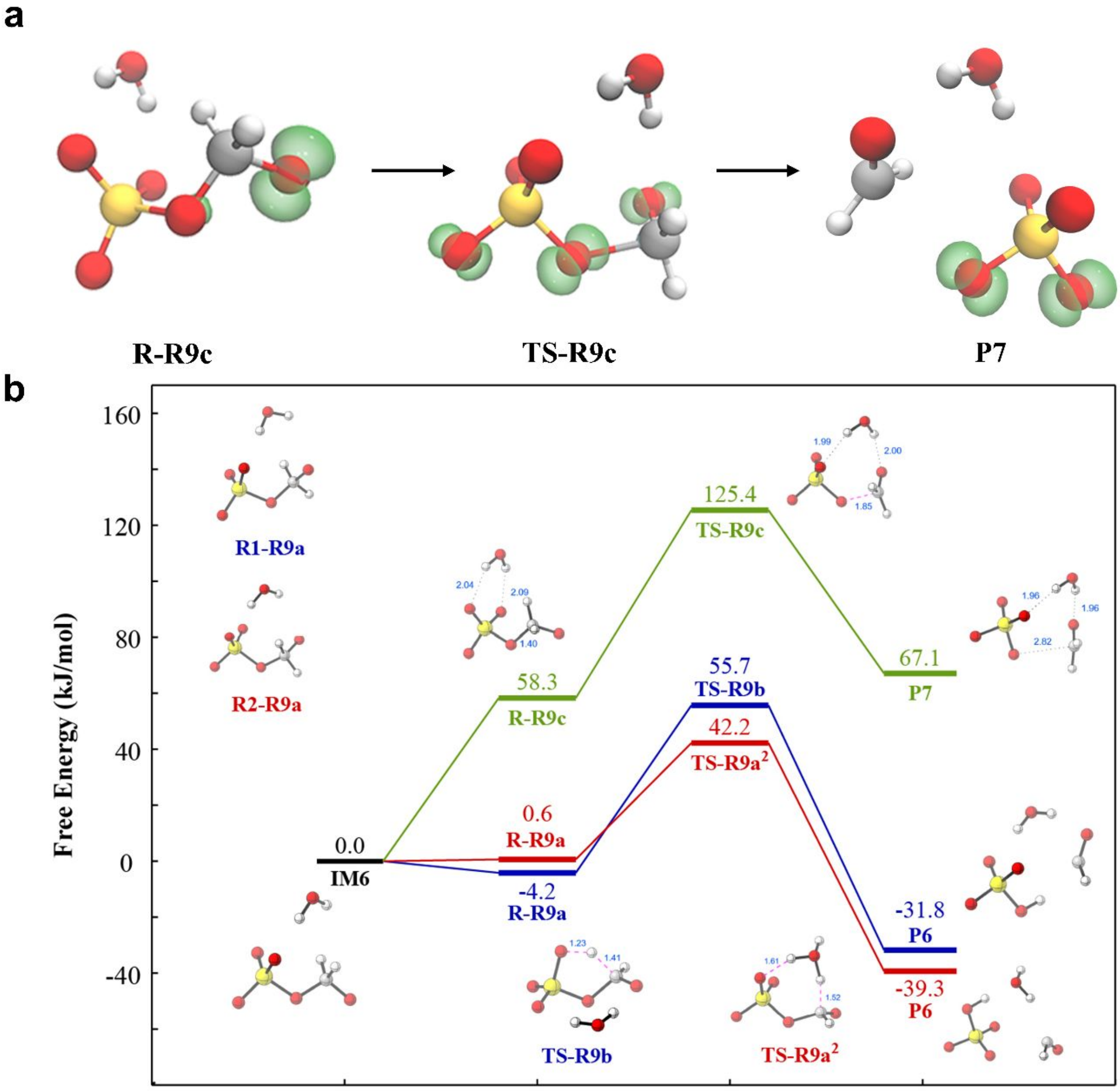

Figure S9. a) Spin density isosurfaces at isovalue = 0.02 (green: α-spin). b) Free energy profiles of reaction **R-R9a2**, **R-R9b,** and **R-R9c** stands for the reaction pre-complex of alkoxy radical and 1 explicit water molecule.

In contrast, the **R9c** was found to be highly sensitive to solvation geometry. Among all configurations sampled, viable **TS-R9c** were located **only** in the SMD with 1-4 explicit H_2O molecules. In these cases, the $\Delta G^{\ddagger}$ is consistently high, around 80 kJ/mol, and the ΔG is relatively unfavorable, ranging from +10-20 kJ/mol. Two factors likely account for this: (1) The requirement of a specific hydrogen-bonding motif involving both the alkoxy and sulfate oxygen to stabilize **TS-R9c** and suppress **TS1-R9a**. (2) The product of the **R9c** pathway, $SO_3^{\bullet -}$, is highly unstable in aqueous environments. Due to its high energy and strong reactivity, it is likely to undergo rapid secondary reactions with surrounding water molecules, converting to more stable species such as HSO_4^-. This instability further disfavors the **R9c** channel under aqueous conditions.

Effects of Low Water Activity and Acidic Conditions on the R9 Pathways
In the main text, the **R9** family of pathways (**R9a**, **R9a2**, **R9b**, **R9c**) is modeled in bulk water using an SMD implicit solvent model on top of an explicit hydrogen-bonded water cluster of n=1-6 molecules. While this treatment is appropriate for cloud droplets and dilute deliquesced particles, atmospheric aerosols frequently exist in highly non-ideal regimes characterized by (1) reduced water activity ($a_w \ll 1$, as in efflorescing or viscous secondary organic aerosol) and (ii) strongly acidic compositions (pH $\lesssim 0$, as in sulfate-rich tropospheric particles). To assess the robustness of the mechanistic picture proposed in the main text under such conditions, we performed two additional sets of calculations on the **R9** mechanisms:

1. Low-water-activity limit ("gas-phase" model). All **R9** transition states and products were re-optimized using the same explicit water clusters (n=1-6) but without the SMD implicit solvent. This effectively removes long-range dielectric stabilization and mimics the limiting case in which the reacting moiety samples only its first solvation shell, as expected at low a_w.
2. Low-pH limit ("acidic" model). The methyl-sulfate-derived intermediate **IM6** (•$OCH_2OSO_3^-$) was protonated to its neutral form •OCH_2OSO_3H, and the same gas-phase explicit-cluster calculations were repeated. Protonation of the sulfate headgroup is the thermodynamically expected speciation at a very low pH typical of sulfate aerosols on each **R9** channel.

All settings (functional, basis set, dispersion correction, frequency analysis, and standard-state corrections) are identical to those used in the main text; only the solvent description and protonation state were varied.

For **R9a**, we found that the gas-phase reaction without the implicit solvent can reach the HSO_4^- product in one step rather than two. The pathway for **IM6** has higher barriers (The computed barriers are summarized in **Tables S7–S14**). If we start with protonated **IM6** in the gas phase (implying an even more acidic situation), •OCH_2-O-SO_3H, the excess proton more likely remains in the water cluster, and the inorganic sulfate remains as HSO_4^- instead of H_2SO_4, shown by the positive ΔG values (see **R9a-acid** pathway in the **Figure S10**).

For **R9a2**, with protonated **IM6**, the barriers are relatively smaller for small water cluster sizes (e.g., 14.73 kJ/mol at n = 3, **Table S10**), reflecting the well-known role of pre-protonation in turning –OSO_3H into a much better leaving group: the α-H is transferred intramolecularly through a single water bridge to a sulfate oxygen while C–O cleaves concertedly, yielding neutral •CHO + H_2SO_4 with no net charge separation along the reaction coordinate. This is precisely the type of pathway favored under gas-phase conditions, and it explains the apparent reversal relative to the aqueous phase, where the long-range, water-relayed **R9a** is cheaper than the intramolecular **R9a2** because bulk-like solvation makes proton relay essentially free.

For **R9b**, we found that the kinetics are slightly faster in the gas phase without the implicit solvent, but the final inorganic sulfate product remains as HSO_4^-. However, H_2SO_4 can form when IM6 is protonated, though the kinetics are generally slower than those of **R9a** or **R9a2**.

For **R9c**, the kinetics are much slower in the gas phase without the implicit solvent but are slightly faster with a protonated **IM6**. However, as before, **R9c** proceeds more slowly than the **R9a** and **R9a2** pathways.

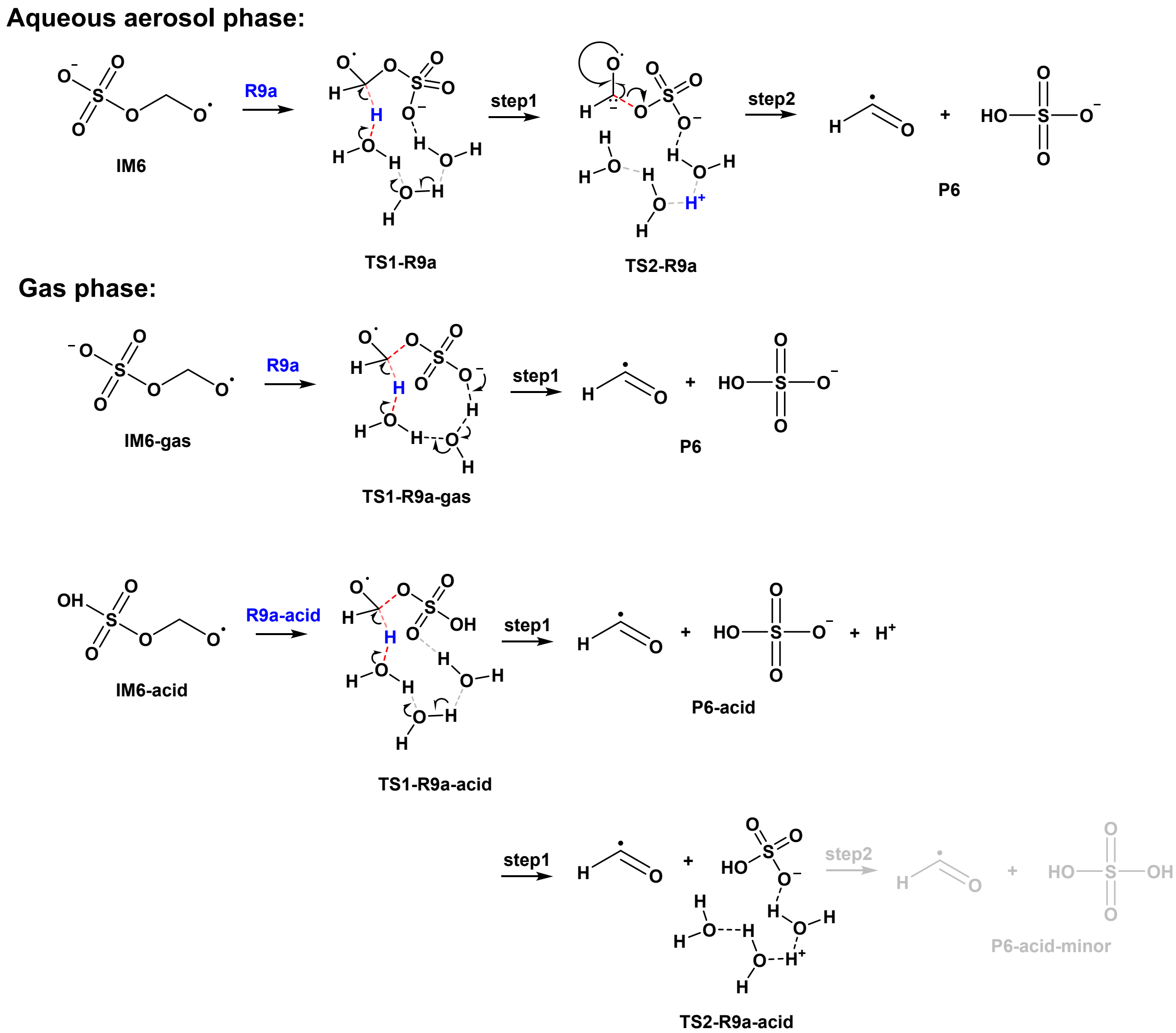

Figure S10. Schematic mechanisms of the **R9a** pathway for the OH-initiated oxidation intermediate **IM6** (•$OCH_2OSO_3^-$/•OCH_2OSO_3H) under three representative atmospheric regimes considered in this work. **Top (**aqueous aerosol phase, **IM6):** water modeled with explicit H_2O molecules and SMD implicit solvent model. **Middle** (low-water-activity/gas-phase, **IM6):** explicit H_2O molecules only. **Bottom** (low-pH/gas phase, **IM6-acid** •OCH_2OSO_3H): explicit H_2O molecules only. The migrating proton is highlighted in blue, breaking/forming bonds are drawn as dashed lines, and bridging water molecules in each TS illustrate the minimum hydrogen-bonded network required to support the proton relay.

Table S7. Computed $\Delta G^{\ddagger}$ and ΔG for the **R9a** pathway in gas phase (no implicit solvent) at 298 K and 1 bar. "n" stands for the number of explicit water molecules in the calculation.

•OCH_2-O-SO_3^- + n H_2O → **TS1-R9a^n-gas** → •CHO + HSO_4^- + n H_2O, where n = 3-6		
n	$\Delta G^{\ddagger}$ (kJ/mol)	ΔG (kJ/mol)
3	56.72	-54.93
4	35.97	-61.33

5	52.48	-68.63
6	40.13	-64.25

Table S8. Computed $\Delta G^‡$ and ΔG for the **R9a-acid** pathway in gas phase (no implicit solvent) at 298 K and 1 bar. "n" stands for the number of explicit water molecules in the calculation.

•OCH_2-O-SO_3H + *n* H_2O → **TS1-R9a^n-acid** → •CHO + HSO_4^-+ H^+ + *n* H_2O → **TS2-R9a^n-acid** → •CHO + H_2SO_4 + *n* H_2O, where *n* = 3, 4 ,5, 6				
n	Step1 $\Delta G^‡$ (kJ/mol)	Step1 ΔG (kJ/mol)	Step2 $\Delta G^‡$ (kJ/mol)	Step2 ΔG (kJ/mol)
3	22.63	-78.58	7.39	9.62
4	26.18	-96.78	23.48	23.39
5	23.39	-80.64	11.91	4.46
6	37.90	-91.05	NA	NA

Table S9. Computed $\Delta G^‡$ and ΔG for the **R9a2** pathway in gas phase (no implicit solvent) at 298 K and 1 bar. "*n*" stands for the number of explicit water molecules in the calculation.

•OCH_2-O-SO_3^- + *n* H_2O → **TS-R9a2^n-gas** → •CHO + HSO_4^- + *n* H_2O, where *n* = 1-6		
n	$\Delta G^‡$ (kJ/mol)	ΔG (kJ/mol)
1	54.87	-6.08
2	61.88	-40.06
3	61.49	-39.83
4	63.19	-37.41
5	51.14	-41.94
6	46.55	-50.60

Table S10. Computed $\Delta G^‡$ and ΔG for the **R9a2-acid** pathway in gas phase (no implicit solvent) at 298 K and 1 bar. "*n*" stands for the number of explicit water molecules in the calculation.

•OCH_2-O-SO_3H + *n* H_2O → **TS-R9a2^n-gas-acid** → •CHO + H_2SO_4 + *n* H_2O, *n* = 1-6		
n	$\Delta G^‡$ (kJ/mol)	ΔG (kJ/mol)
1	47.05	-61.83
2	38.38	-53.48
3	14.73	-74.27
4	NA	NA
5	NA	NA
6	NA	NA

Table S11. Computed $\Delta G^‡$ and ΔG for the **R9b** pathway in gas phase (no implicit solvent) at 298 K and 1 bar. "*n*" stands for the number of explicit water molecules in the calculation.

•OCH_2-O-SO_3^- + *n* H_2O → **TS-R9b^n-gas** → •CHO + HSO_4^- + *n* H_2O, where *n* = 1-6		
n	$\Delta G^‡$ (kJ/mol)	ΔG (kJ/mol)
1	31.4	-36.9
2	43.6	-22.8
3	46.9	-39.8

4	54.9	-33.3
5	49.9	-39.7
6	60.5	-21.9

Table S12. Computed $\Delta G^{\ddagger}$ and ΔG for the **R9b-acid** pathway in gas phase (no implicit solvent) at 298 K and 1 bar. "*n*" stands for the number of explicit water molecules in the calculation.

•OCH_2-O-SO_3H + *n* H_2O → **TS-R9b^n-acid** → •CHO + H_2SO_4 + *n* H_2O, *n* = 1-6		
n	$\Delta G^{\ddagger}$ (kJ/mol)	ΔG (kJ/mol)
1	47.1	-61.8
2	55.4	-57.6
3	59.6	-70.9
4	63.9	-52.4
5	54.9	-33.3
6	NA	NA

Table S13. Computed $\Delta G^{\ddagger}$ and ΔG for the **R9c** pathway in gas phase (no implicit solvent) at 298 K and 1 bar. "*n*" stands for the number of explicit water molecules in the calculation.

•OCH_2-O-SO_3^- + *n* H_2O → **TS-R9c^n-gas** → HCHO + $SO_4^{\bullet-}$ + *n* H_2O, where *n* = 1-6		
n	$\Delta G^{\ddagger}$ (kJ/mol)	ΔG (kJ/mol)
1	123.32	47.61
2	125.91	52.98
3	67.79	-1.00
4	126.85	51.00
5	NA	NA
6	NA	NA

Table S14. Computed $\Delta G^{\ddagger}$ and ΔG for the **R9c-acid** pathway in gas phase (no implicit solvent) at 298 K and 1 bar. "*n*" stands for the number of explicit water molecules in the calculation.

•OCH_2-O-SO_3H + *n* H_2O → **TS-R9c^n-gas-acid** → •CHO + $HSO_4^{\bullet-}$ + *n* H_2O, *n* = 1-6		
n	$\Delta G^{\ddagger}$ (kJ/mol)	ΔG (kJ/mol)
1	40.85	-57.53
2	NA	NA
3	NA	NA
4	NA	NA
5	NA	NA
6	NA	NA

Electron Paramagnetic Resonance Spectroscopy

EPR measurements were conducted using a Bruker EMXMicro spectrometer equipped with an ER4119HS microwave cavity and operating at 9.8 GHz and a modulation frequency of 100 kHz. The instrumental settings were as follows: field sweep, 100 G; microwave frequency, 9.8 GHz; microwave power, 12.62 mW; modulation amplitude, 1.0 G; conversion time, 5.13 ms; time constant, 5.12 ms; receiver gain, 30 dB; and number of scans, 5. EPR spectra were analyzed using the SpinFit module of the Xenon software (Bruker).

Radical Generation

Aqueous solutions containing ~10 mM BMPO, ~25 mM sodium methyl sulfate, and ~20 mM H_2O_2 were prepared in 1 mL of ultrapure water. The mixture was vortexed for 1 minute to ensure homogeneity. A 50 μL aliquot of the mixture was put into a capillary tube and placed in the EPR cavity. In situ irradiation was performed using a 100 W mercury lamp with a cutoff filter at 300 nm resulting in a wavelength range of 280–700 nm, as previously described.[6] Under these conditions, OH are generated primarily via the photolysis of H_2O_2, and any ensuing radical reactions with methyl sulfate are immediately captured by BMPO.

The evolution of radical species was measured every 70 s via EPR spectroscopy. All experiments were performed at room temperature (approximately 23 ± 1 °C) unless otherwise stated.

EPR detection results: Validation of BMPO for radical species (SO_3•⁻/ SO_4•⁻ and OH)

Before analyzing the complex methyl sulfate system, we first established the spectral fingerprints of the relevant radicals, including sulfite/sulfate radicals ($SO_3^{\bullet -}$/ $SO_4^{\bullet -}$) and OH to validate the detection capabilities of the BMPO spin trap.

We first measured the EPR spectra of Na_2SO_3 solutions under UV irradiation to observe any radical signals. The EPR results were shown in **Figure S11a**. Our EPR spectrum roughly aligns with the literature spectra from Ranguelova *et al.*,[7] although we do not have OH radicals in our system. At lower concentration of spin traps (<50 mM based on Ranguelova *et al.*,) there is aqueous sulfite chemistry that leads to the more complex spectra. Further spin-fitting analysis shows that our overall spin fits seem reasonable but revealed that our BMPO–SO_3 parameters (A_N = 13.66 G, A_H = 15.2 G) deviate from published data obtained with DMPO as the trapping agent,[8] presumably due to differences in spin-trap chemistry. Nonetheless, these findings confirm that BMPO–sulfite/sulfate radical adducts are detectable using EPR.

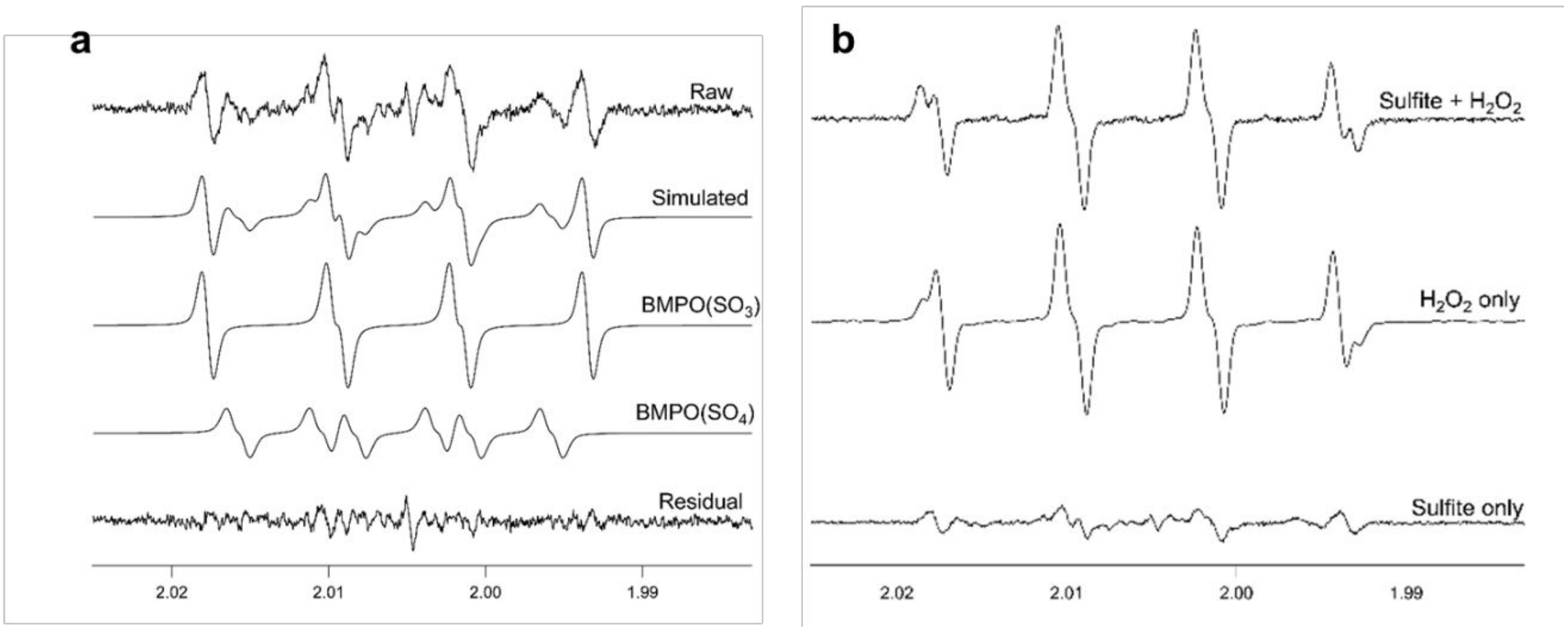

Figure S11: a) EPR results from irradiation of Na_2SO_3, deconvoluted to show the contributions from SO_3 and SO_4 radicals, b) EPR results from sulfite + H_2O_2 + UV compared to irradiation of only H_2O_2 or only sulfite

Next, we tested pure BMPO–OH radical signals as a control. Because BMPO–OH is most commonly reported in the literature its peak shape and parameters are relatively well-established.[9] We similarly obtained comparable values in this study (**Figure S11b**, BMPO-OH 1: A_N = 14.21 G, A_H = 15.56 G, A_{H2} = 0.58 G; BMPO-OH 2: A_N = 14.13, A_H = 12.72, A_{H2} = 0.70), thereby confirming the reliability of our experimental conditions and instrumentation. However, this signal significantly overlaps with the sulfite radical signal. To further investigate whether sulfite radicals can be detected under OH-containing conditions, we irradiated a mixed solution of 5 mM sulfite and 2 mM H_2O_2 with UV light. We found significant overlap between the outer peaks of BMPO–SO_3 radical and BMPO–OH, and conventional spectral fitting methods were insufficient to clearly differentiate the two. Although slight splitting was observed at the outermost peaks of the EPR spectrum, the fitting software still attributed the signals to BMPO–OH rather than BMPO–SO_3. This suggests that the differences in chemical shift or hyperfine splitting between BMPO–SO_3 and BMPO–OH are not large enough to allow unambiguous discrimination in complex systems containing OH.

EPR detection results for the methyl sulfate system

With these benchmarks established, we examined the methyl sulfate + H_2O_2 + UV system. In the *in situ* EPR experiments, we focused on how the radical concentration in solution varies with irradiation time (**Figure S12a**). Upon photo-irradiation, the spectra revealed a predominant BMPO–OH adduct signal. The concentration of BMPO–OH increased markedly over the irradiation period and reached a maximum value of approximately 3.0×10^{16} molecules/cm^3 at around 600 s, indicating that H_2O_2 effectively produces OH under these conditions.

At extended irradiation time (becoming discernible above the limit of detection after ~3 minutes) additional peaks emerged in the EPR spectra, corresponding to BMPO–R adducts formed with carbon-centered organic radicals. While clearly present, these signals are significantly lower in intensity compared to the BMPO–OH adduct and are partially overshadowed by the magnitude of the hydroxyl signal (**Figure S12b**). The potential formulas of the adducts were identified using mass spectrometry, as described below (e.g., R stands for $\bullet CH_3O$ or $\bullet CHO$).

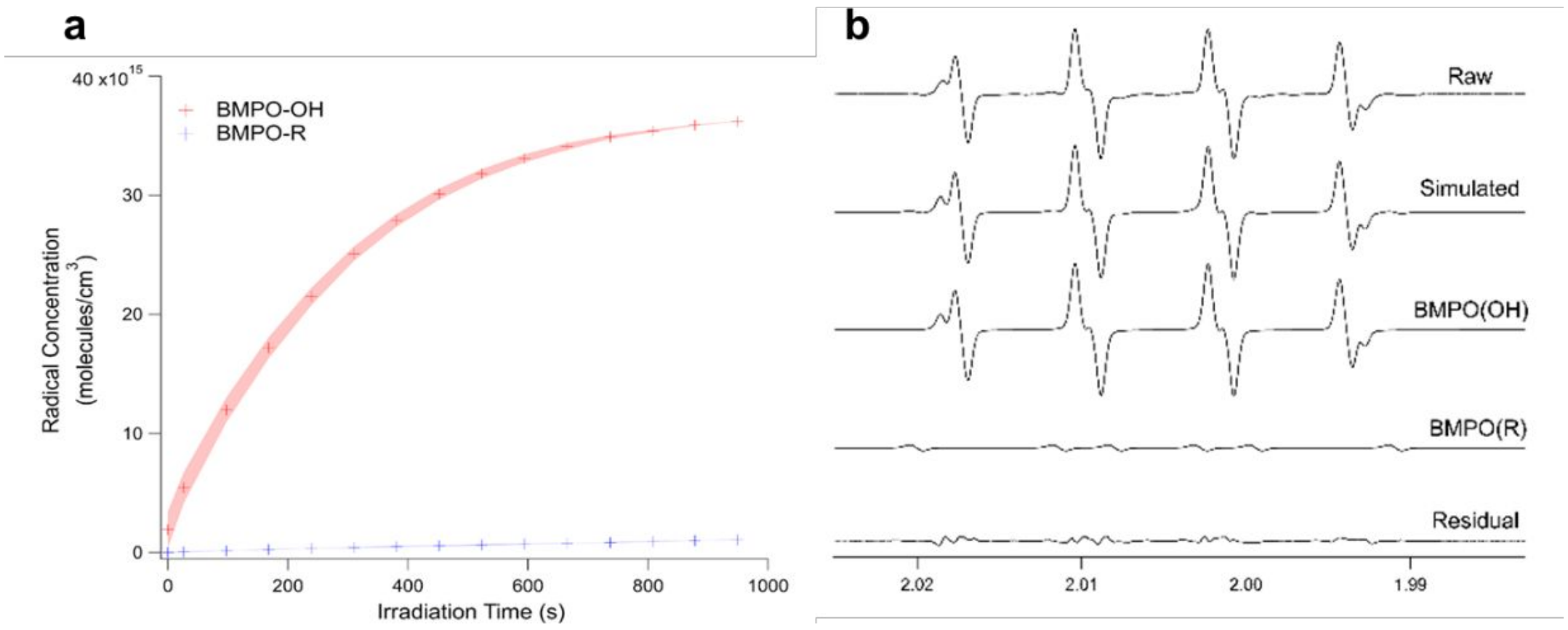

Figure S12. a) Time-dependent formation of BMPO–OH and BMPO–R adducts during photo-irradiation in EPR experiments, b) EPR results from Methyl sulfate + H_2O_2 + UV and the simulated signal showing contributions from BMPO(OH) and BMPO(R) radicals.

Assessment of sulfite/sulfate radical formation

A primary objective was to determine if EPR could directly detect the sulfite radicals proposed in pathways **R2**/**R4** or sulfate radicals from pathway **R9c**. As shown in **Figure S12b**, there is no direct observable evidence of distinct sulfite radical peaks in the reaction mixture. However, drawing on our validation results (**Figure S11b**), this absence does not preclude their formation. Instead, it is highly probable that any sulfite radical signal is masked by the spectral congestion and the overwhelming intensity of the BMPO–OH signal. Consequently, to definitively clarify the potential products in this reaction system and overcome the limitations of EPR resolution, we conducted additional characterization using Mass Spectrometry.

MS detection results: Validation of BMPO for radical species (SO_3•⁻/ SO_4•⁻)

Similar to the EPR approach, we began our mass spectrometry (MS) experiments with a relatively simple system for validation. Specifically, we irradiated a solution containing sulfite and the spin-trapping agent BMPO and analyzed the resulting solution using MS to see whether a BMPO–sulfite adduct could be detected in the mass spectrum. The rationale behind this step is to verify, in the absence of other potential interferents, whether BMPO can reliably capture and identify sulfur-centered radical adducts. This foundational test also provides an essential reference for subsequent analyses of methyl sulfate + H_2O_2 + UV system.

Table S15. MS detection of bisulfite, bisulfate, and BMPO adducts

m/z	Formula	Compound
**80.965**	HSO_3^-	Bisulfite
**96.960**	HSO_4^-	Bisulfate
**142.051**	$C_6H_8O_3N^-$	BMPO fragment
**280.086**	$C_{10}H_{18}O_6NS^-$	BMPO + HSO_3^-
**296.081**	$C_{10}H_{18}O_7NS^-$	BMPO + HSO_4^-

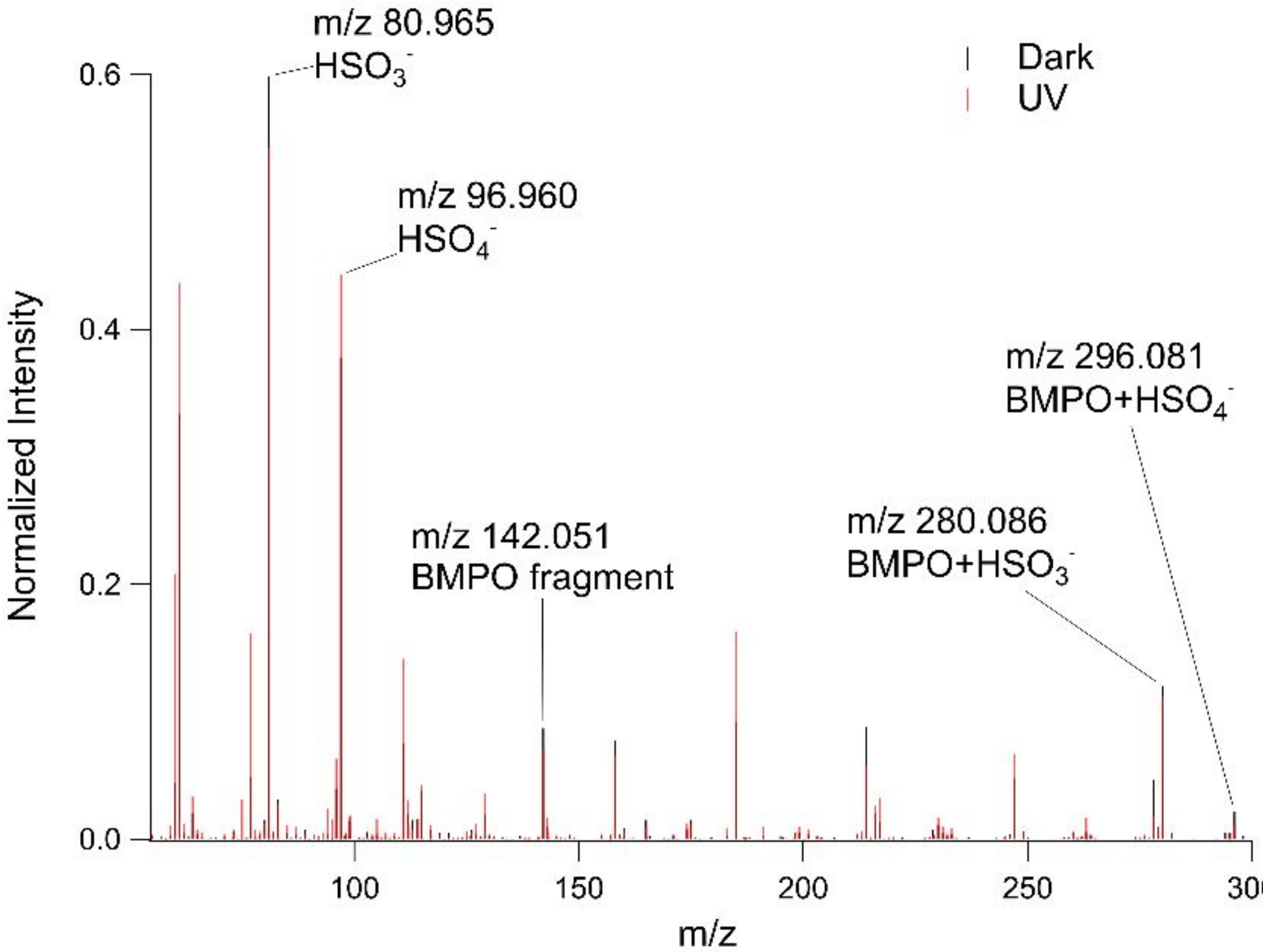

Figure S13. Direct infusion mass spectra of dark and irradiated solutions of sulfite + BMPO, normalized to the sum of the intensity of m/z 80.965 + m/z 96.960.

As shown in **Table S15** and **Figure S13**, both bisulfite (m/z 80.965) and bisulfate (m/z 96.960) are readily detectable in negative ion mode. Upon UV irradiation, the bisulfate peak becomes more prominent, consistent with the aqueous coupling reactions of sulfite radicals described in the literature.[6] Both the dark and irradiated solutions contain a noticeable peak at m/z 280.086, assigned to BMPO + HSO_3^-. Similarly, sulfate can complex with BMPO to form m/z 296.081, although this signal exhibits a much lower

relative abundance in our measurements. The appearance of these peaks in the dark sample suggests non-radical sulfite and sulfate may form clusters with BMPO. These cannot be differentiated from the BMPO-radical adducts using direct infusion, but would be separated by LCMS. Based on these observations, any system containing sulfite/sulfate and BMPO should produce signals at m/z 280.086 and 296.081, unless the concentrations of the clusters or adducts fall below the mass spectrometric detection limit.

MS detection results: BMPO+ methyl sulfate +H_2O_2 system with/without UV

To investigate the radical intermediates formed during the oxidation of methyl sulfate, we analyzed the reaction mixtures using both direct infusion and LC-MS. Consequently, the absence of signals at m/z 280.086 and 296.081 in the methyl sulfate + H_2O_2 + UV system, indicates that the concentrations of sulfur-centered radical adducts or ion clusters under those conditions are too low for accurate detection.

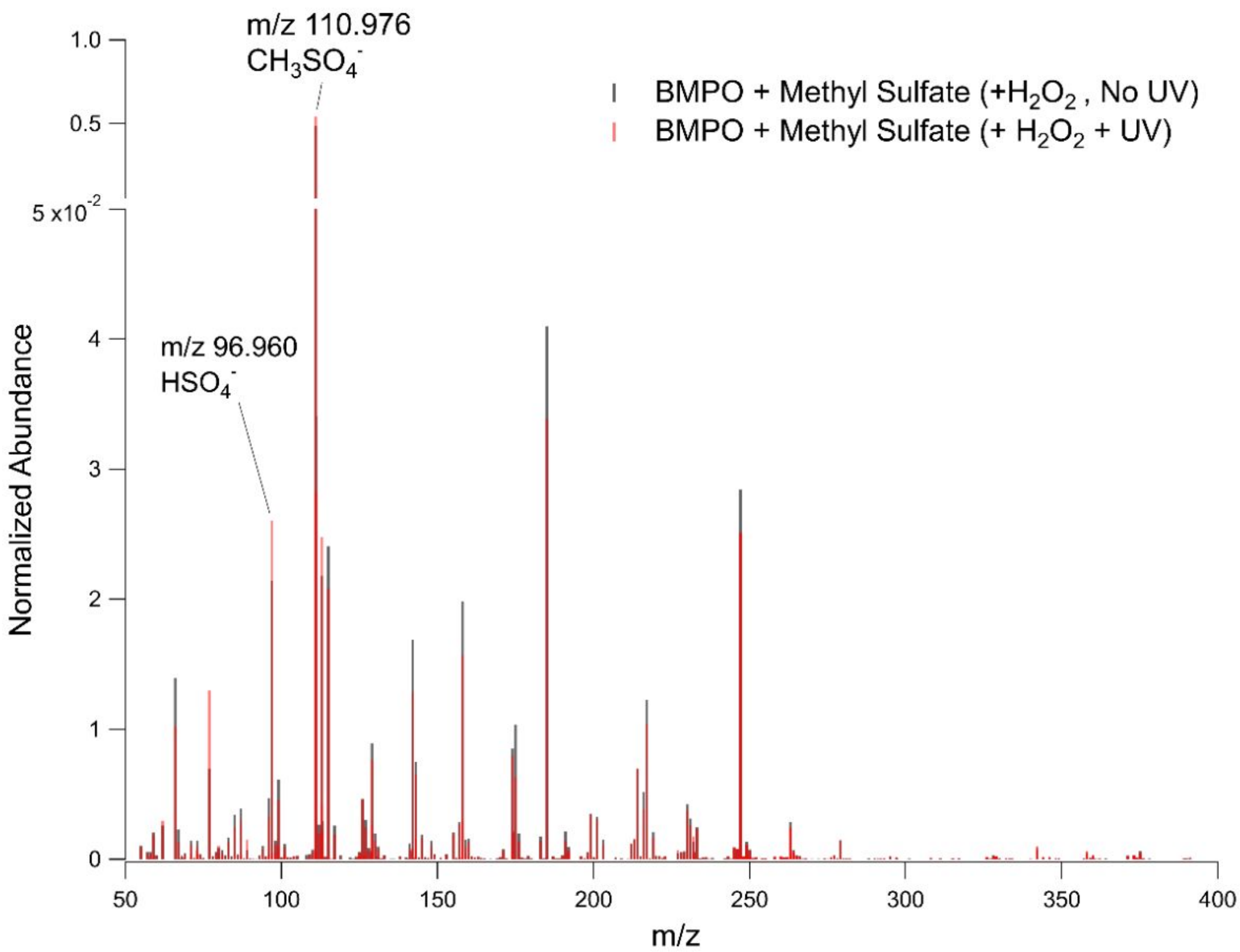

Figure S14. Negative ion mode direct infusion mass spectra for dark and irradiated mixtures of BMPO+ methyl sulfate +H_2O_2 normalized to the intensity of methyl sulfate.

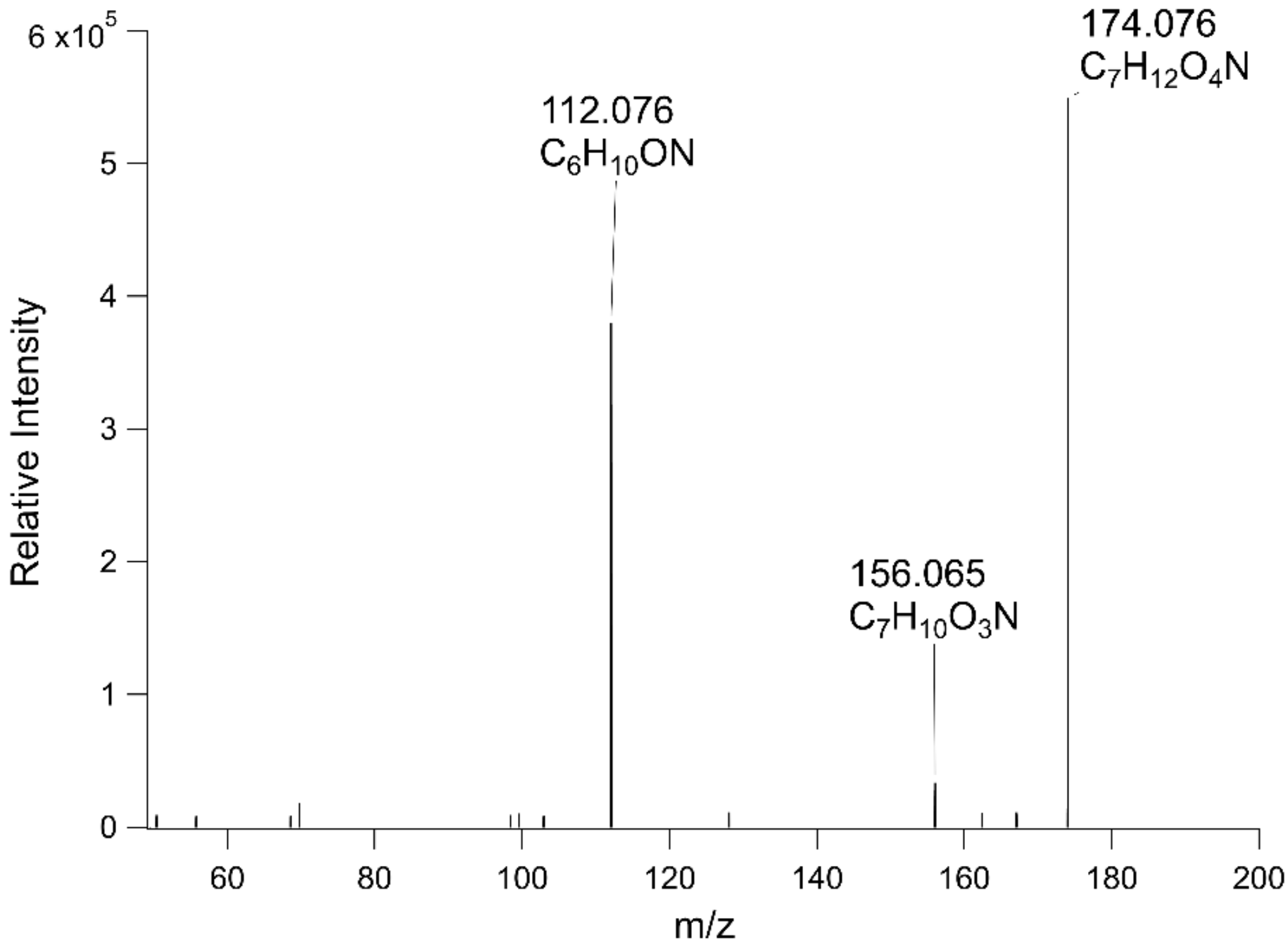

Figure S15. Sum of the fragmentation mass spectra recorded at collision energies 10, 30, and 50 for m/z 230.139, BMPO–CH_3O detected in positive ion mode.

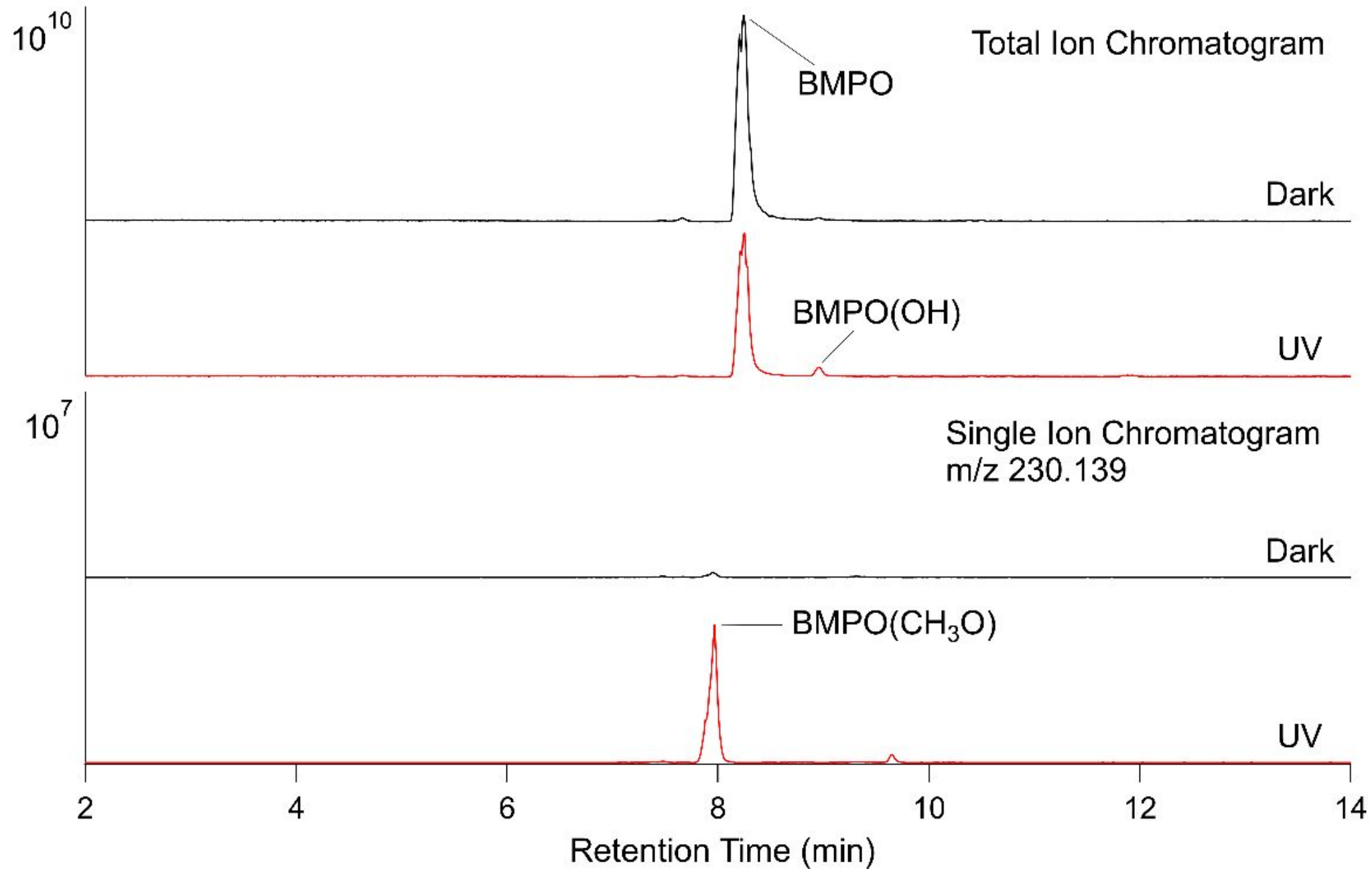

Figure S16. Positive ion mode chromatograms for dark and irradiated mixtures of BMPO+ methyl sulfate +H_2O_2 with major peaks labeled.

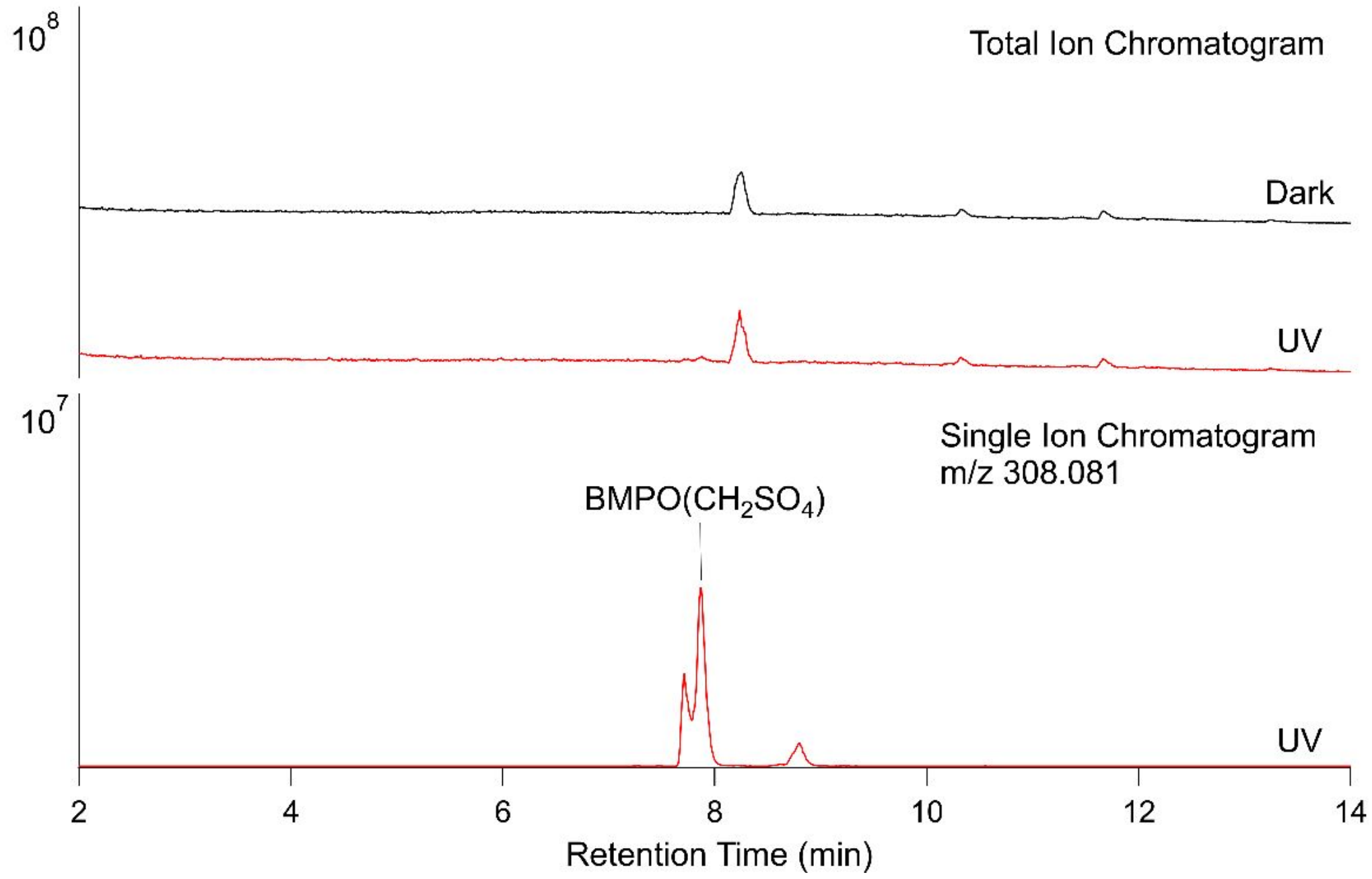

Figure S17. Negative ion mode chromatograms for dark and irradiated mixtures of BMPO+ methyl sulfate +H_2O_2. There is no dark SIC for m/z 308.081 because it was not detected. Unlabeled peaks in the TIC could not be assigned to a mass formula and are considered background signal as they appear in both dark and irradiated samples.

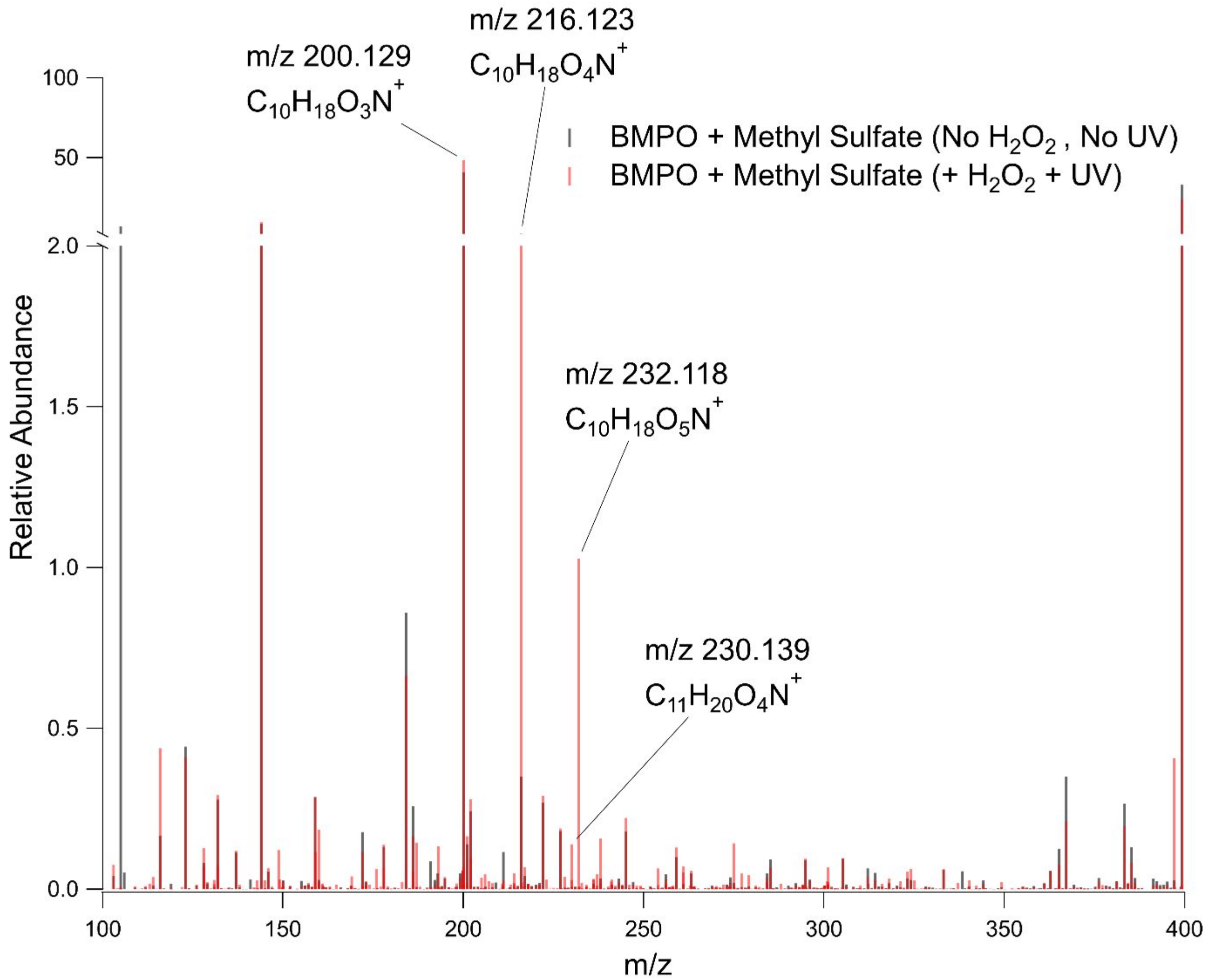
m/z 200.129
$C_{10}H_{18}O_3N^+$
m/z 216.123
$C_{10}H_{18}O_4N^+$
BMPO + Methyl Sulfate (No H_2O_2 , No UV)
BMPO + Methyl Sulfate (+ H_2O_2 + UV)
m/z 232.118
$C_{10}H_{18}O_5N^+$
m/z 230.139
$C_{11}H_{20}O_4N^+$
Relative Abundance
100
50
2.0
1.5
1.0
0.5
0.0
100
150
200
250
300
350
400
m/z

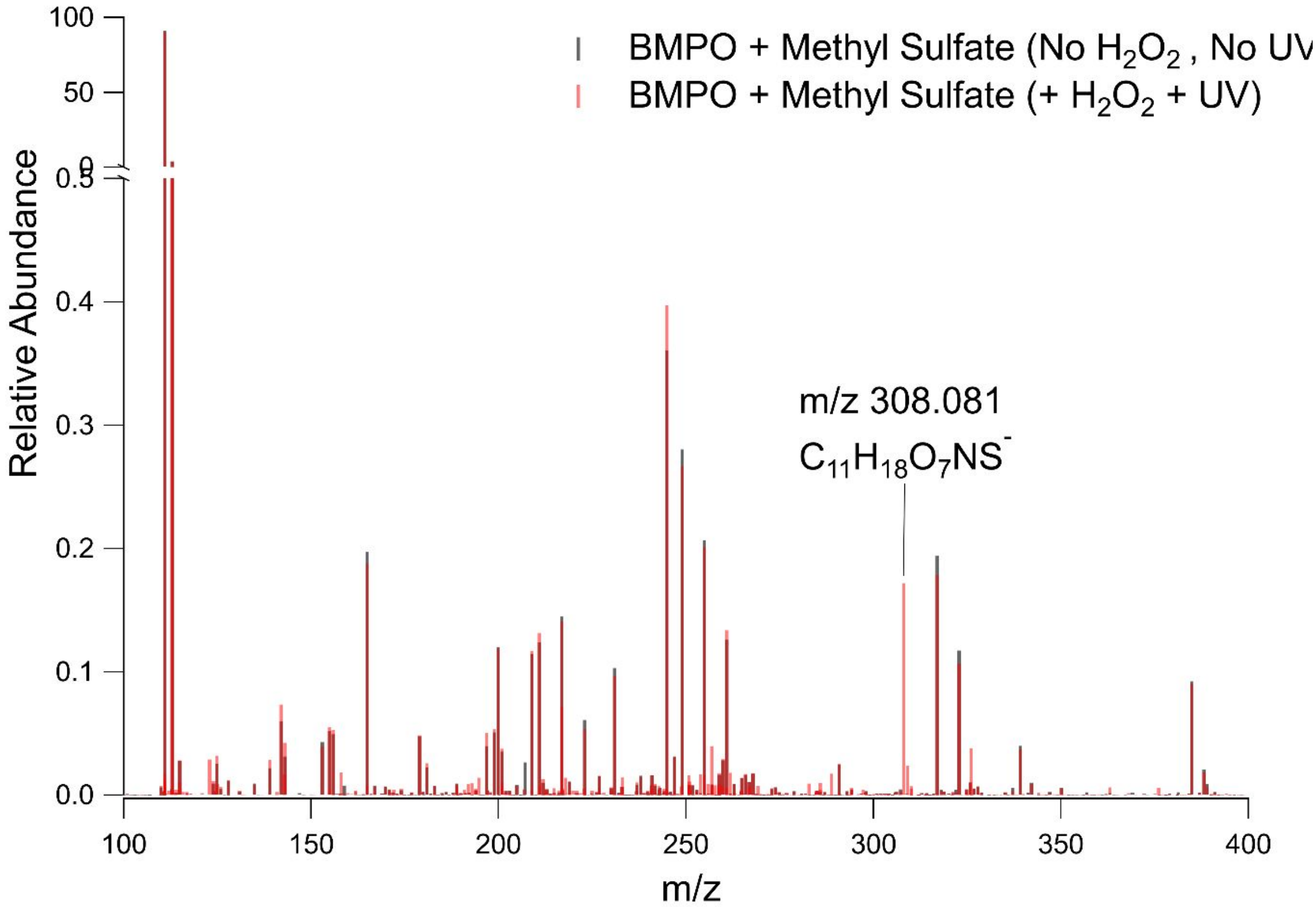

Figure S18. Refer to **Figure 11** in the main text. Positive ion and negative ion mode LCMS results for methyl sulfate + H_2O_2 + UV (red) and the dark control system (black) with major BMPO adducts labeled.

Oxidation Kinetics: Rate Constants for the Kinetic Model at 298 K for the main reaction pathways

Based on transition state theory and the steady-state approximation, the intrinsic rate constants for the key OH-initiated reactions were evaluated under the assumption of a two-step mechanism. This mechanism involves: (i) rapid formation of a pre-reaction complex between the reactants and (ii) unimolecular transformation of the complex to products via a transition state. This can be described as:

Step 1, K_{eq}: R ↔ pre-reactive complex
Step 2, k_{uni}: pre-reaction complex → TS → product

Where K_{eq} and k_{uni} are the equilibrium constant of step 1 and the rate constant of step 2, respectively. K_{eq} and k_{uni} are defined as follows:

$$K_{eq} = \frac{1}{c^{\circ}}\exp\left(-\frac{\Delta G_{eq}}{RT}\right)$$

$$k_{uni} = \kappa\frac{k_{B}T}{h}\exp\left(-\frac{\Delta G^{\ddagger}}{RT}\right)$$

where c° is the standard concentration, ΔG_{eq} is the Gibbs free energy change associated with formation of the pre-reactive complex from the initial configuration, κ is the tunneling correction factor, calculated using the Skodje–Truhlar method.[10] k_B and h corresponds to Boltzmann's constant and Planck's constant, respectively. Accordingly, the intrinsic rate constant is described by:

$$k = K_{eq}k_{uni}$$

Because these reactions occur in liquid aerosol particles, the observable bimolecular rate constant is additionally constrained by the diffusion-controlled encounter limit in the condensed phase. The diffusion-limited rate constant, k_{diff}, was estimated using the Smoluchowski expression[11],

$$k_{diff} = 4\pi R_{AB}(D_{A} + D_{B})N_{A}$$

where R_{AB} is the encounter distance between the two reactants, here we employed the the sum of vdW radii for two species. D_{A} and D_{B} are diffusion coefficients for two reactants, and N_{A} is Avogadro's constant. The diffusion coefficients are given by the Stokes-Einstein equation:

$$D = \frac{k_{B}T}{6\pi\eta R}$$

where η is the vicosity of the solution. The total bimolecular rate constant k_{total} was then obtained from the Collins–Kimball theory:[12]

$$k_{total} = \frac{k \times k_{diff}}{k + k_{diff}}$$

Table S16. Numerical values of vdW radius and diffusion coefficients of reactants in the aqueous-phase

Reactants	vdW radius (m)	Diffusion coefficient (m^2 s^{-1})
**Methyl sulfate**	3.56×10^{-10}	6.89×10^{-10}
**•OH**	1.86×10^{-10}	1.32×10^{-9}
**IM2**	3.73×10^{-10}	6.57×10^{-10}
**IM3**	3.88×10^{-10}	6.32×10^{-10}
**IM6**	3.84×10^{-10}	6.39×10^{-10}
**IM6-22w**	3.56×10^{-10}	6.89×10^{-10}
**R-R9a-step2**	3.85×10^{-10}	6.36×10^{-10}
**O_2**	2.11×10^{-10}	1.16×10^{-9}
**O_3**	2.63×10^{-10}	9.33×10^{-10}
**H_2O**	1.96×10^{-10}	1.25×10^{-9}
**H_3O^+**	2.04×10^{-10}	1.20×10^{-9}

Table S17. Reaction schemes and rate constants for the kinetic model at 298 K

No.	Reaction	k_{diff} (M^{-1} s^{-1})	K_{eq}	k_{uni} (s^{-1})	k_{total}
**R1a**	R + •OH → CH_3O• + $HSO_4^–$	8.22×10^{9}	2.16×10^{-1}	3.53×10^{-16}	7.62×10^{-17} s^{-1} M^{-1}
**R1b**	RH + •OH → R• + H_2O	8.22×10^{9}	2.34×10^{-1}	2.21×10^{3}	5.17×10^{2} s^{-1} M^{-1}
**R2**	R• → HCHO + SO_3•$^–$		8.12×10^{-1}	3.00×10^{-1}	2.43×10^{-1} s^{-1}
**R3**	R• + O_2 → ROO•	8.04×10^{9}	3.63×10^{1}	6.44×10^{10}	8.01×10^{9} s^{-1} M^{-1}
**R4***	ROO• → O_2 + HCHO + SO_3•$^–$		3.68×10^{-1}	1.13×10^{-28}	4.16×10^{-29} s^{-1}
**R5**	2ROO• → ROOOOR	7.42×10^{9}	9.22×10^{-6}	4.86×10^{4}	4.48×10^{-1} s^{-1} M^{-1}
**R7**	ROOOOR → RO• + ROOO•		2.43	3.61 s^{-1}	8.76 s^{-1}
**R8**	RO• + ROOO• → 2RO• + O_2		4.29×10^{-1}	1.36×10^{12}	5.83×10^{11} s^{-1}
**R10**	R• + O_3 → ROOO•	7.65×10^{9}		No $\Delta G^‡$	7.65×10^{9} s^{-1} M^{-1}
**R6a**	RO_4R → ROH + ROOOH		9.38×10^{-6}	1.69×10^{-18}	1.59×10^{-23} s^{-1}
**R6b**	RO_4R → ROH+RO• + HO_2•		1.52×10^{-4}	4.28×10^{-9}	6.49×10^{-13} s^{-1}
**R9a-step1**	RO• → •O(H)$COSO_3^{2–}$ ···H^+	8.29×10^{9}	9.52×10^{-1}	1.01×10^{8}	9.50×10^{7} s^{-1} M^{-1}
**R9a-step2**	•O(H)$COSO_3^{2–}$···H^+ → •CHO + $HSO_4^–$	8.19×10^{9}	1.04	5.80×10^{13}	8.19×10^{9} s^{-1} M^{-1}
**R9a-22w-step1****	RO• → •O(H)$COSO_3^{2–}$ + H^+	8.10×10^{9}	2.42×10^{14}	2.87×10^{13}	8.10×10^{9} s^{-1} M^{-1}
**R9a-22w-step2****	•O(H)$COSO_3^{2–}$ + H^+ → •CHO + $SO_4^{2–}$ + H^+		1.54×10^{3}	5.17×10^{11}	8.10×10^{9} s^{-1}

* **R4** could only be found without 6 explicit water molecules with SMD implicit water model.

** The optimization level was set to be M06-2X-D3(0)/def2-SVP to speed up the calculations.

For **R1a,** This large $\Delta G^{\ddagger}$ likely stems from the substantial energy required to cleave the strong S–OCH_3 bond. The total reaction rate is 7.62×10^{-17} s^{-1} M^{-1}. As such, **R1a** is not expected to contribute significantly to the oxidation of methyl sulfate anion. Compared to **R1a**, the total reaction rate of **R1b** is 5.17×10^{2} s^{-1} M^{-1}, indicating the dominance of this reaction.

The corresponding total decomposition rate of **R2** is 2.43×10^{-1} s^{-1}. Although the relatively high barrier renders it kinetically less favorable, it remains accessible and may serve as a **minor contributor** to inorganic sulfate formation via subsequent $SO_3\bullet^-$ initiated reactions in the aerosol phase. In contrast, the calculated overall rate constant of **R3** is 8.01×10^{9} s^{-1} M^{-1}. Interestingly, this rate is comparable to that reported by Adam Marchaj for the $CH_3\bullet + O_2$ system (ca. 3×10^{9} s^{-1} M^{-1}), suggesting a similar reactivity trend despite structural differences.[9]

For **R5**, the calculated overall bimolecular rate constant is 4.48×10^{-1} s^{-1} M^{-1}, corresponding to a unimolecular rate constant of 4.86×10^{4} s^{-1} and a small K_{eq} of 9.22×10^{-6} M^{-1}. This may be attributed to the steric hindrance introduced by the bulky sulfate ester groups in $\bullet OOCH_2OSO_3^-$, which can impede efficient radical-radical association in the condensed phase.

For **R6** reaction, our calculated rate constants further support the conclusion in main text: **R6a** and **R6b** exhibit extremely slow rates of 1.59×10^{-23} s^{-1} M^{-1} and 6.49×10^{-13} s^{-1} M^{-1}, respectively. These results indicate that **Russell-type mechanisms** are unlikely to contribute to the overall reaction pathways in the presence of sulfate ester functionalities.

For **TS1-R9a[6]** and **TS2-R9a[6]** reaction, the calculated rate constants for the two steps (9.50×10^{7} s^{-1} M^{-1} and 8.19×10^{9} s^{-1} M^{-1}, respectively) indicate a **rapid, sequential reaction** under aqueous conditions.

Cartesian coordinates of all species

Initial OH reactions with methyl sulfate via R1a or R1b:

R

C	3.52518900	-1.30509400	-0.85532800
H	4.13575400	-1.56351700	-1.71510800
H	3.95951800	-0.45623400	-0.32938000
H	3.43126900	-2.16239900	-0.19069900
O	2.23638300	-0.94967900	-1.38908600
S	1.13557400	-0.51628000	-0.34007800
O	0.95470100	-1.63159300	0.57107500
O	1.65316600	0.67031600	0.33456300
O	-0.01180900	-0.24047700	-1.18186800
O	-4.09573300	0.39630500	-0.20276900
H	-3.50436100	0.09935000	0.55546000
O	-2.53020000	-1.43643600	-1.83829000
H	-3.11502500	-0.80402500	-1.38662600
H	-1.64757600	-1.03699200	-1.77971200
O	-2.50105300	-0.51334100	1.73212300
H	-2.13858800	-1.34412000	1.36664300
H	-1.74664700	0.09222200	1.86473400
O	-1.61632300	-2.86042900	0.45178600
H	-0.68774400	-2.57367400	0.42191700
H	-2.00210300	-2.53198000	-0.38120700
O	-0.61198400	1.55977900	1.94886100
H	-1.08525000	1.90578300	1.17019600
H	0.25413400	1.30033300	1.59655400
O	0.75912000	3.12308600	-0.84490700
H	0.82173500	3.80139200	-0.16176600
H	1.14479800	2.32522400	-0.44208300
O	-1.81647200	2.04734400	-0.60114400
H	-0.99037900	2.52416900	-0.81722000
H	-1.59664000	1.12357600	-0.79336400

R1a: $CH_3OSO_3^{-}$ + •OH → CH_3O• + HSO_4^{-}

IM1a

C	1.82075600	-2.07937900	-0.34404600
H	2.59459700	-2.82545400	-0.49871400
H	1.61918000	-1.96608500	0.72071500
H	0.91595800	-2.36215600	-0.88206500
O	2.35064200	-0.85228100	-0.87845900
S	1.37529500	0.39275000	-0.85450900
O	0.21340000	0.03286400	-1.65035900
O	1.02912500	0.60528200	0.55364500
O	2.17046000	1.45638700	-1.41700500
O	-2.97928900	-0.39276300	1.76814600
H	-2.03778000	-0.55400400	2.06544700
O	-1.83541600	-1.64985900	-0.64130500
H	-2.33801100	-1.17042500	0.03714700
H	-1.14596900	-1.03104500	-0.94103900

O	-0.41758700	-1.01619700	2.41428600
H	-0.42816300	-1.92711100	2.06007600
H	0.14696900	-0.51374800	1.80274600
O	-0.81623600	-3.50287300	1.15689100
H	-0.03480200	-3.96860800	0.83715600
H	-1.11944600	-2.94984300	0.40724700
O	-1.77428300	1.68148900	0.32418200
H	-1.35376000	2.26726000	-0.33026700
H	-1.04781900	1.11281300	0.61623200
O	0.92523700	3.37655700	1.29659100
H	0.11659400	3.46587100	1.81583300
H	1.02159200	2.41852100	1.15786000
O	-0.18664500	3.46095700	-1.30621400
H	0.25332800	3.61083800	-0.44795300
H	0.45507300	2.94580500	-1.81314000

TS1a

C	2.72401600	-0.99862600	-0.32003500
H	3.55151800	-1.53422200	-0.78353800
H	3.11090300	-0.14006300	0.23098800
H	2.21393900	-1.66809900	0.37428000
O	1.87074200	-0.57114600	-1.37257200
S	0.27833700	-0.06849700	-0.97300600
O	-0.01274200	-1.58970900	-1.04176300
O	0.62085200	0.53960600	0.31861100
O	0.13317500	0.73543200	-2.17682500
O	-1.45273500	-0.07164300	-0.65458300
H	-1.58402200	-0.29111800	0.29722700
O	-3.05216800	-2.27401800	-1.45634500
H	-2.50641900	-1.47333000	-1.34434000
H	-2.71238300	-2.70290600	-2.25045000
O	-1.73883500	-0.93985500	1.95587500
H	-1.75411400	-1.87827300	1.67433100
H	-0.86318600	-0.79293400	2.33359400
O	-1.94220200	-3.46814300	0.82020100
H	-1.04731100	-3.74035200	0.58476100
H	-2.33139200	-3.13000600	-0.01205800
O	-1.37022600	2.36774600	1.43131500
H	-1.42631200	2.75038200	0.53883000
H	-0.75152600	1.63222800	1.30427000
O	1.56111900	3.23526100	0.06777900
H	1.34948100	3.67402100	0.90059300
H	1.33074900	2.30147400	0.21692600
O	-0.96480200	3.26979900	-1.28650400
H	-0.08414200	3.47407200	-0.92402800
H	-0.83686000	2.39552600	-1.69026300

P1

C	2.43986600	-1.49800100	-0.34582900
H	3.35958100	-2.07584900	-0.54733300

H	2.50741300	-1.10454600	0.67209600
H	1.59715700	-2.18661200	-0.46684600
O	2.43817900	-0.50419100	-1.30275900
S	-0.73166600	0.31107100	-1.06205100
O	-0.73890100	-0.97324800	-1.74465500
O	0.06788300	0.27513500	0.16955100
O	-0.37724200	1.41595300	-1.93311400
O	-2.19499400	0.61312500	-0.60698000
H	-2.40734700	0.14882400	0.28533600
O	-0.77619400	-3.53263900	-0.56746900
H	-0.73353200	-2.67142500	-1.02345000
H	0.04487300	-3.98119600	-0.80233100
O	-2.67199100	-0.51462600	1.63196100
H	-1.91681900	-1.12046900	1.80443100
H	-2.61002700	0.17694900	2.30421200
O	-0.36425000	-2.03186800	1.77795000
H	0.03725800	-1.28900600	1.29495500
H	-0.50654100	-2.70211800	1.08199200
O	-0.85909200	2.28019300	2.07352000
H	-0.58640900	2.90296900	1.37744800
H	-0.64617000	1.41964000	1.68250000
O	2.28834500	1.90259200	0.85053600
H	2.08936700	2.04155800	1.78468600
H	1.62273300	1.25524800	0.55541900
O	0.27127500	3.65528200	-0.21145000
H	1.09655500	3.26383700	0.12408700
H	-0.02857200	3.01094200	-0.87481000

R1b: $CH_3OSO_3^-$ + •OH → $•CH_2OSO_3^-$ + H_2O
IM1b

C	3.52871300	-1.07372500	-0.41861000
H	4.31036600	-1.49665700	-1.04219500
H	3.81804100	-0.07890900	-0.08050700
H	3.33020100	-1.72954300	0.42819600
O	2.36483000	-0.98597900	-1.26341300
S	1.03079100	-0.50395300	-0.57091100
O	0.70769900	-1.47311000	0.46091500
O	1.32270400	0.81243400	0.00480700
O	0.09077200	-0.45667100	-1.66719300
O	2.87243600	0.59228200	2.37368500
H	2.27766800	0.78103800	1.61231700
O	-2.75457500	-0.34047000	-1.16754200
H	-2.86299200	-0.61450800	-0.23700200
H	-1.81577700	-0.49859500	-1.36105700
O	-2.72564700	-1.08586400	1.58538500
H	-2.26054900	-1.88232400	1.27849600
H	-2.03543200	-0.41534900	1.75522600
O	-1.50292300	-3.17884200	0.02565500
H	-0.67911800	-2.66285000	0.06944700
H	-1.97458700	-2.82378200	-0.73845700

O	-1.01285900	1.12292400	1.87595400
H	-1.44128000	1.61069200	1.14099400
H	-0.11195300	0.96685700	1.56014500
O	0.26394400	2.96348600	-1.52690600
H	0.58281300	3.80362500	-1.17714800
H	0.73461700	2.28005200	-1.01928900
O	-2.20371600	2.31762500	-0.34514000
H	-1.39439000	2.58873300	-0.81971600
H	-2.45979100	1.46138500	-0.73613900

TS1b

C	3.36716100	-1.11939000	-0.37005300
H	4.15484000	-1.36117400	-1.07930600
H	3.79423200	-0.25717400	0.31159700
H	3.11308400	-1.95147700	0.28612100
O	2.26178400	-0.67131900	-1.10262100
S	0.90305700	-0.42267300	-0.27564400
O	0.67484700	-1.62918400	0.48386700
O	1.18836300	0.73675400	0.57077700
O	-0.05139000	-0.14433300	-1.31628700
O	4.00115700	0.82118100	1.16463000
H	3.04787200	1.03635400	1.20367700
O	-2.84657900	-0.64108800	-1.30738400
H	-3.09321900	-0.63109700	-0.36067600
H	-1.87879100	-0.56379700	-1.32370500
O	-3.17428100	-0.82673000	1.47714600
H	-2.65689500	-1.64549200	1.42185500
H	-2.56828000	-0.12246800	1.76662600
O	-1.79835400	-3.03529700	0.26791900
H	-0.89559300	-2.67769400	0.28860300
H	-2.20320100	-2.64238000	-0.51641500
O	-1.48913300	1.45992100	1.65662700
H	-1.90074300	1.72342300	0.80871200
H	-0.59033700	1.17471900	1.43907800
O	0.11854000	3.21703300	-0.44652600
H	-0.29731700	3.37474600	0.40839000
H	0.64510200	2.41552000	-0.30037100
O	-2.50693700	2.19232200	-0.85993200
H	-1.61951800	2.43550100	-1.16535200
H	-2.72724800	1.33750700	-1.26395800

IM2

C	3.50926700	-1.37703600	-0.23312000
H	4.40510800	-1.51330100	-0.81680500
H	4.39784100	1.09823700	0.38341900
H	3.34413100	-1.86932300	0.71476800
O	2.40340800	-1.11800600	-1.02216900
S	1.00470500	-0.86651800	-0.27473400
O	0.73345500	-2.05935100	0.49773200
O	1.22360100	0.30746900	0.56594200

O	0.10708300	-0.63649000	-1.37972000
O	3.74026400	1.79700800	0.48665000
H	2.90006600	1.31973500	0.56420300
O	-2.75709700	-0.17196500	-1.37239200
H	-3.03017100	-0.47938900	-0.48740300
H	-1.81782300	-0.41083300	-1.42676300
O	-3.15335000	-1.07328100	1.30419700
H	-2.68039900	-1.86502200	0.99064700
H	-2.46216100	-0.43858300	1.57596200
O	-1.84110900	-3.15370700	-0.11279300
H	-0.92445800	-2.87062800	0.04363100
H	-2.08073100	-2.75198700	-0.95779900
O	-1.32427400	0.98867000	1.85150300
H	-1.65845600	1.55907500	1.13064100
H	-0.41665100	0.77358300	1.58872800
O	0.54725800	2.71630000	-0.88950600
H	0.94124100	3.48466900	-0.45984000
H	0.81637700	1.95618200	-0.34780600
O	-2.20422400	2.43700900	-0.40282300
H	-1.27922600	2.61204600	-0.66111800
H	-2.44367600	1.60543500	-0.85322700

Alkyl sulfate radical (•$CH_2OSO_3^-$): Fragmentation via R2 vs O_2/O_3 addition

R2: •$OOCH_2OSO_3^-$ → HCHO + SO_3•$^-$
IM2-R2

C	-3.71233200	-0.57513500	-0.34477400
H	-4.77101700	-0.43515400	-0.48911200
H	-3.12357300	-1.26899800	-0.92849700
O	-3.08581700	0.56949700	0.11469800
S	-1.51184300	0.46566300	0.41028800
O	-0.89931600	0.09294500	-0.85788600
O	-1.19139200	1.80730400	0.84333700
O	-1.36127900	-0.55437200	1.42662300
O	-0.18486400	-2.69433000	-0.77391500
H	-0.29090700	-2.90903200	0.16153000
H	-0.48863300	-1.77357600	-0.84898400
O	1.44982400	-1.13428700	1.88755200
H	0.49510200	-1.13094500	1.71277500
H	1.86584600	-1.42758500	1.05446100
O	2.52643800	-1.85037100	-0.65223700
H	2.43836000	-0.92934000	-0.95728200
H	1.65716100	-2.25103700	-0.83662700
O	1.87132900	0.88160500	-1.20606700
H	1.98420800	1.17756100	-0.28043000
H	0.94321400	0.59564700	-1.24138300
O	1.77876500	1.62642500	1.51311600
H	1.69986700	0.68573400	1.77184700
H	0.86576500	1.94414300	1.45924700
O	0.30405500	3.40417900	-1.10625200

H	-0.23253400	2.92454900	-0.45658900
H	0.97357000	2.75891000	-1.37544100

TS-R2

C	-3.59013900	-0.21643900	-0.86982400
H	-3.69435400	0.55173900	-1.63564200
H	-3.74075300	-1.26264200	-1.13477300
O	-3.23704900	0.09819600	0.30262000
S	-1.19488600	0.11644400	0.59448200
O	-0.71339100	-0.26175200	-0.73914000
O	-0.84749800	1.48355300	0.97834800
O	-0.92194200	-0.88303600	1.62018700
O	0.25325000	-2.98518900	-0.72335200
H	0.22525500	-3.23665400	0.20837100
H	-0.15285000	-2.10244400	-0.75258700
O	2.00259200	-1.17828400	1.79574600
H	1.03858600	-1.27887300	1.75883800
H	2.33219600	-1.48680700	0.92934800
O	2.86524600	-1.91271000	-0.80738600
H	2.68843500	-1.01818600	-1.14911900
H	2.01717300	-2.38339600	-0.91250000
O	1.93320000	0.73114300	-1.43842400
H	2.13841200	1.06315800	-0.54096200
H	1.03641700	0.36706500	-1.34878100
O	2.14696100	1.56132300	1.24470300
H	2.15527800	0.63170600	1.55187500
H	1.21347400	1.81768400	1.28244200
O	0.18843800	3.13154500	-1.20901300
H	-0.22517000	2.65431600	-0.47341300
H	0.86911300	2.51978000	-1.52493200

P2

C	-3.57931400	-0.97185900	-0.85369100
H	-4.02821100	0.02750000	-0.95240400
H	-3.04162100	-1.36606600	-1.72775200
O	-3.68705500	-1.61201800	0.16351500
S	-1.40907300	0.78928400	0.98553400
O	-1.14997800	0.43326400	-0.42644200
O	-0.78292300	2.06333700	1.37310800
O	-1.13109400	-0.31587400	1.90982600
O	-0.55327200	-2.39928000	-0.68416900
H	-0.72690100	-2.77251300	0.18910400
H	-0.78921500	-1.45969500	-0.60065700
O	1.67590200	-1.15756800	1.79601100
H	0.70904000	-1.08146800	1.80711800
H	1.90139300	-1.46885200	0.89805800
O	2.20334100	-1.88888700	-0.89801100
H	2.14278400	-0.95906700	-1.18189900
H	1.27938700	-2.20160200	-0.92816600
O	1.61510900	0.88016700	-1.34321400

H	1.91903900	1.13873500	-0.44862800
H	0.67628700	0.66684400	-1.21624200
O	2.18930700	1.54854300	1.32432700
H	2.07640300	0.61774400	1.60670700
H	1.33504700	1.96705800	1.50530400
O	0.35397900	3.55452000	-0.87987700
H	-0.06682900	3.11982600	-0.12230200
H	0.86846500	2.84792600	-1.29516000

R3: $\bullet CH_2OSO_3^- + O_2 \rightarrow \bullet OOCH_2OSO_3^-$

IM2b

C	2.07480400	-0.98354700	1.51599600
H	1.56319400	-0.27380000	2.15170000
H	3.04439600	-1.38677200	1.75905700
O	4.24095000	0.30174800	-1.30019100
O	3.42200600	-0.53335000	-1.09222600
O	1.29136800	-1.88388300	0.81698200
S	-0.10713900	-1.33878000	0.24654800
O	0.23678300	-0.32296700	-0.73616000
O	-0.70147100	-2.52749600	-0.31277300
O	-0.81521600	-0.79416800	1.39363100
O	-3.12852200	-1.44451900	-1.63164600
H	-2.76052200	-0.63173200	-2.00722500
H	-2.35720400	-1.94851400	-1.32835300
O	1.81845700	2.05031000	0.11043000
H	1.53863300	1.23531500	-0.33493400
H	1.32082800	2.02284100	0.94481800
O	-1.97450900	1.42324800	-1.36567900
H	-2.56572400	1.19511200	-0.62501700
H	-1.19026200	0.86601700	-1.22217300
O	-0.54942600	3.66725800	-0.45656100
H	-1.11517900	2.98697300	-0.86808500
H	0.33944000	3.27529300	-0.46819800
O	-3.50880500	0.16486700	0.71226500
H	-3.58457800	-0.49728100	0.00181900
H	-2.69830700	-0.09262400	1.17761700
O	-0.44436400	2.00921800	1.96904500
H	-0.71060700	2.58466500	1.23303300
H	-0.65671400	1.10414100	1.68825500

TS-R3

C	2.38537600	-1.00055900	1.05519400
H	2.01635700	-0.06423400	1.45150400
H	3.27890600	-1.47231900	1.43067500
O	4.30432400	0.48298100	-0.82940600
O	3.37666500	-0.27142700	-0.94603600
O	1.47016900	-1.90491200	0.57637700
S	0.01527200	-1.34172600	0.17090400
O	0.25289500	-0.34164600	-0.85649700
O	-0.65281300	-2.53028400	-0.29489400

O	-0.53601600	-0.77661000	1.39040900
O	-3.20841400	-1.60038300	-1.45603200
H	-2.93171500	-0.73847500	-1.80026400
H	-2.38174800	-2.03975600	-1.20234800
O	1.55422700	2.21111300	-0.06056500
H	1.32938900	1.37159300	-0.49094900
H	1.07425400	2.16551700	0.78373800
O	-2.15670300	1.26305100	-1.20362100
H	-2.62298000	0.99823600	-0.38952900
H	-1.30908400	0.78894600	-1.16190200
O	-0.91201900	3.69743700	-0.56863000
H	-1.43798500	2.94056000	-0.88834100
H	0.00264800	3.37140300	-0.59197200
O	-3.39374300	-0.11596500	1.00613200
H	-3.52202500	-0.75385500	0.28174400
H	-2.53184100	-0.35584400	1.37889900
O	-0.58147600	2.07268300	1.90667900
H	-0.95380800	2.61924400	1.19638000
H	-0.70373600	1.15163900	1.62541400

IM3-R3

C	2.57358700	-0.73627300	0.62992000
H	2.11232500	0.14863500	1.07050400
H	3.36824000	-1.14454900	1.24801900
O	4.05661500	0.55928500	-0.45830600
O	3.15761800	-0.34913300	-0.63991000
O	1.66361100	-1.75405200	0.34449600
S	0.13268800	-1.31237000	0.08165700
O	0.19002900	-0.30919000	-0.96840800
O	-0.47955900	-2.55673400	-0.30481900
O	-0.33404400	-0.77597300	1.34795500
O	-3.24880400	-1.82331600	-1.10149200
H	-3.05984700	-0.96483900	-1.50799800
H	-2.37702700	-2.22052300	-0.95191500
O	1.40021200	2.35768500	-0.45800900
H	1.13600600	1.50665100	-0.83986500
H	1.08006800	2.29852500	0.45768100
O	-2.34565400	1.10934500	-1.11938600
H	-2.70356300	0.85341300	-0.24923100
H	-1.47076100	0.68718200	-1.15380200
O	-1.23456900	3.62726100	-0.55189700
H	-1.72133100	2.83117600	-0.83722600
H	-0.30568300	3.40581700	-0.72785800
O	-3.24353000	-0.20632800	1.27680100
H	-3.41311500	-0.89978600	0.61462100
H	-2.33562700	-0.38003600	1.56742800
O	-0.39219200	2.07109200	1.81817400
H	-0.90223100	2.56156100	1.15319300
H	-0.48983900	1.13280100	1.58881800

R4: •OOCH$_2$OSO$_3^-$ → HCHO + SO$_3$•$^-$···O$_2$ → HCHO + SO$_5$•$^-$
(SMD implicit solvent model without 6 explicit water molecules):

IM3

C	0.94914400	-0.65183200	-0.31156200
H	0.77764900	-1.14153300	0.64423800
H	1.47586100	-1.27709600	-1.02660900
O	1.82097500	0.47550400	-0.08196300
O	1.73106700	0.92641900	1.13053900
O	-0.23134400	-0.23110000	-0.92310400
S	-1.43429800	0.37828700	0.00268900
O	-2.54927300	0.29407800	-0.90920100
O	-1.51289400	-0.50132600	1.14951000
O	-1.02688500	1.72859600	0.32546400

TS-R4:

C	1.60240400	-1.37143000	-0.49777600
H	1.65035500	-1.84846100	0.49603400
H	2.58921300	-1.41016400	-0.98976100
O	1.55394800	0.14825900	-0.07235900
O	2.43903900	0.48644100	0.79829100
O	0.55135600	-1.58608100	-1.22517300
S	-1.17170300	0.42487800	0.16737600
O	-1.82380000	0.18649000	-1.06531000
O	-1.10065400	-0.58918400	1.15191500
O	-0.74710400	1.73901500	0.48262900

P3

C	1.09252800	-1.58695400	-0.60199000
H	1.09258600	-1.69230300	0.49297100
H	1.97127900	-1.10876200	-1.05895700
O	0.41332700	1.15355400	-0.07718400
O	1.16396700	1.30334000	0.96458800
O	0.17446900	-1.98771000	-1.27366600
S	-1.26895200	1.00012000	0.34494300
O	-1.80179200	0.82931900	-0.97784800
O	-1.28252600	-0.15994900	1.19356800
O	-1.55488600	2.24934400	0.99357600

R5: 2 •OOCH$_2$OSO$_3^-$ → $^-$O$_3$SOCH$_2$OOOOCH$_2$OSO$_3^-$

IM3-R5

C	-1.88652900	-1.63141000	-1.48073500
H	-2.34150900	-2.59962000	-1.28076400
H	-2.61201500	-0.82674400	-1.56573400
O	-0.96680800	-1.25674900	-0.50040300
S	0.07297900	-2.36969200	0.06464500
O	1.07547800	-2.52009100	-0.96795800
O	0.55270100	-1.71611000	1.26493900
O	-0.71364800	-3.56122900	0.29725200
O	-1.23757100	-1.79107800	-2.75809300
O	-0.84232500	-0.65365100	-3.23667900

O	-2.90664000	1.24593800	0.03589000
O	-2.80251300	2.11343500	-0.92459100
C	-1.46407800	2.17324700	-1.45049200
H	-1.57438700	2.50281800	-2.47910400
H	-1.02009700	1.18513300	-1.35729200
O	-0.72428300	3.15746100	-0.78219000
S	-0.03456200	2.79487200	0.63929300
O	0.48867100	1.44975000	0.48178600
O	0.97728800	3.81856900	0.74818800
O	-1.08645200	2.88300500	1.62976800
O	1.17285700	1.30280000	3.94999100
H	1.80136700	1.56822700	3.25534600
H	0.41703100	0.89498400	3.49617400
O	-1.11921000	-4.99893300	-2.20524600
H	-0.94002000	-4.29448600	-2.83913400
H	-0.96780200	-4.58974400	-1.33890800
O	1.82796500	0.23657700	-1.72800700
H	1.26798200	0.69539500	-1.07842200
H	1.51394300	-0.68105200	-1.72234800
O	3.11796400	2.13958400	2.02925500
H	3.12798400	1.28801300	1.54736900
H	2.57751800	2.73512000	1.49009600
O	3.13867700	-0.37492700	0.79275400
H	2.94779800	-0.15000100	-0.13388200
H	2.32360500	-0.80929100	1.09298800
O	-1.17956200	0.12881300	2.70018400
H	-1.32539200	0.94904000	2.20349800
H	-0.65640300	-0.43797400	2.11056800

TS-R5

C	-1.95567700	-1.86839400	-1.05924900
H	-1.58528500	-2.74712300	-1.58636200
H	-2.98959300	-1.97781400	-0.74631700
O	-1.18689700	-1.57281700	0.08265100
S	0.30973900	-2.17547700	0.11650200
O	0.92901300	-1.76989900	-1.13035300
O	0.84745100	-1.53782900	1.30104400
O	0.15215900	-3.60988600	0.22793200
O	-1.86786500	-0.80006900	-1.99313400
O	-2.79182900	0.11671500	-1.75285200
O	-2.14296000	1.39645300	-0.59549600
O	-1.88532400	2.45498300	-1.34829000
C	-0.51855400	2.48805700	-1.75427000
H	-0.50568500	2.90367600	-2.75725700
H	-0.11456200	1.47830800	-1.70693900
O	0.20745200	3.38848100	-0.95222300
S	0.58649500	2.95532200	0.55718600
O	1.06208600	1.58600100	0.45775500
O	1.61012000	3.91524000	0.89632700
O	-0.63307000	3.07598900	1.32973200

O	1.44545100	1.69305100	3.96567000
H	2.17105400	1.78278600	3.32309600
H	0.72157800	1.24302300	3.49851400
O	0.41282300	-4.42027600	-2.57336900
H	0.67184300	-3.51936300	-2.80341200
H	0.32489200	-4.38483100	-1.60868300
O	2.62735100	0.47804000	-1.64064100
H	2.15477300	1.06842800	-1.03077000
H	2.07496200	-0.32086200	-1.63407300
O	3.62914900	2.09525800	2.15580500
H	3.58946500	1.23333200	1.69492400
H	3.13552200	2.71169400	1.59510900
O	3.53086000	-0.43916100	0.93910400
H	3.45370400	-0.17682600	0.00534400
H	2.64754400	-0.78046400	1.15502900
O	-0.81313600	0.46893500	2.63693100
H	-0.91964200	1.25974500	2.08457400
H	-0.30036600	-0.15116100	2.09426900

IM4-R5

C	-2.48252700	-1.98418800	-1.04866400
H	-2.09433600	-2.77314300	-1.69404900
H	-3.48018500	-2.21795100	-0.68663600
O	-1.66750900	-1.78057800	0.08958900
S	-0.15084600	-2.30760000	0.00315000
O	0.40030700	-1.75005100	-1.21682400
O	0.40895100	-1.77497400	1.22919800
O	-0.23766500	-3.75415100	-0.03171600
O	-2.51938500	-0.84122100	-1.86462400
O	-3.37910000	0.10063000	-1.25456900
O	-2.61336900	1.04754600	-0.63034200
O	-2.28628000	2.00562000	-1.62043100
C	-0.89465700	2.00932400	-1.83473500
H	-0.76048900	2.31301700	-2.86999400
H	-0.47903100	1.02241300	-1.63675600
O	-0.24893200	3.00539400	-1.06456100
S	0.07344600	2.70547300	0.48377200
O	0.65306200	1.37291800	0.50512200
O	1.00710900	3.75743300	0.81186300
O	-1.19151800	2.78094500	1.18720600
O	0.94994100	1.55836200	3.93381000
H	1.67868100	1.67007800	3.29941400
H	0.25289900	1.07066000	3.46310200
O	-0.27367100	-4.59594700	-2.80847000
H	-0.17600600	-3.75433900	-3.26868100
H	-0.21956100	-4.36523800	-1.86713400
O	2.31430600	0.35287100	-1.56050000
H	1.85490800	0.94374900	-0.94123000
H	1.71827900	-0.41365400	-1.59717100
O	3.14208100	2.01440800	2.11945400

H	3.10251700	1.12801900	1.70914600
H	2.61492800	2.58734200	1.54382400
O	3.08327400	-0.61027800	1.04996000
H	3.06273000	-0.36278800	0.10957400
H	2.19214600	-0.96119500	1.21253100
O	-1.24652100	0.22254000	2.61860000
H	-1.37659100	0.98798100	2.03588300
H	-0.73138600	-0.40942400	2.09189000

R6a:

IM4-R6a

C	0.97494200	-1.29187100	1.29367700
H	0.67353400	-1.79800600	2.20744000
H	0.70091700	-0.23701500	1.31472200
O	2.37322200	-1.47318100	1.17821800
S	3.20352600	-0.34538500	0.38939400
O	2.65429500	-0.29959600	-0.95311600
O	2.99400500	0.88608000	1.12983300
O	4.55286200	-0.86121500	0.46433600
O	0.29002600	-1.81275700	0.18613200
O	0.34474100	-3.22210300	0.21435700
O	-0.49464000	-3.68047700	1.19226300
O	-1.81991100	-3.55098300	0.69678600
C	-2.55195400	-2.84736600	1.66918200
H	-2.38342800	-3.27619000	2.65423900
H	-3.59057500	-2.96141800	1.35709700
O	-2.16460600	-1.49383100	1.75659800
S	-2.87443600	-0.44651600	0.76607600
O	-2.79445500	-1.01864200	-0.56493800
O	-4.24068300	-0.32277700	1.24375700
O	-2.07135000	0.74105100	0.96001700
O	3.75550700	1.92588200	-2.46315700
H	3.27336400	1.17918500	-2.07192800
H	3.92922900	2.51328300	-1.70912500
O	6.19853100	0.81411700	-1.27884600
H	5.50384500	1.07200500	-1.90720000
H	5.77234200	0.15576600	-0.70702700
O	-3.37412800	1.02637100	-2.54588400
H	-3.07815900	0.27999700	-1.99968400
H	-3.18706800	1.80865800	-2.00221600
O	-3.44059300	2.99189900	-0.33563500
H	-4.33021200	2.60110800	-0.35567500
H	-2.91705000	2.36409300	0.18726400
O	-5.71054500	1.20327600	-0.76735100
H	-5.36292900	0.62215100	-0.07130900
H	-5.14509000	1.02359000	-1.53631100
O	4.74842900	2.97172400	0.08654300
H	4.13080700	2.37607000	0.54124200
H	5.45768900	2.38302600	-0.21976800

TS-R6a

C	1.65453000	-0.16310000	-0.08505500
H	0.75460500	-0.02710200	0.51208400
H	1.83351400	0.69824800	-0.72907100
O	2.71514300	-0.38688200	0.81919500
S	4.18424000	0.09227400	0.36991500
O	4.47047800	-0.59185900	-0.87757600
O	4.09459100	1.53299000	0.21613500
O	4.99367100	-0.33808200	1.48767100
O	1.49931800	-1.23699000	-0.97890000
O	0.98547400	-2.36749500	-0.25881000
O	-0.24096800	-2.17179100	0.04787700
O	-2.10510800	-2.35464100	-0.51266700
C	-2.35230600	-1.67092000	0.53516100
H	-1.28059900	-1.69131100	1.12044500
H	-3.01661400	-2.11382500	1.29222000
O	-2.57448800	-0.27933500	0.34320500
S	-4.10098200	0.17670800	0.13525600
O	-4.58617900	-0.56431200	-1.01476200
O	-4.78783300	-0.17434700	1.36583500
O	-3.96678500	1.59958000	-0.08407500
O	6.88647000	0.61139200	-1.99179200
H	6.03868400	0.19082100	-1.77474900
H	6.85115900	1.47248000	-1.54424600
O	7.81385900	0.00793700	0.72538500
H	7.68426500	-0.01015700	-0.23739600
H	6.94468000	-0.22462200	1.08853000
O	-7.13975400	0.64949000	-1.80429700
H	-6.29216800	0.18870000	-1.69750900
H	-6.98743900	1.52581000	-1.41287300
O	-6.61601500	2.81902400	0.09396400
H	-7.05647600	2.14521600	0.63719500
H	-5.68141500	2.55675900	0.09952100
O	-7.65900200	0.23862800	1.04894400
H	-6.74125200	0.00064300	1.25879500
H	-7.70406000	0.17237700	0.08076200
O	6.76995200	2.68424200	0.09977100
H	5.83476900	2.44609700	0.20603100
H	7.25029700	1.94990000	0.51607600

P4

C	0.66669700	-0.49822000	0.27593600
H	-0.31846000	-0.14504800	0.57311100
H	0.84179200	-0.36613200	-0.79192100
O	1.59390100	0.24230600	1.05183400
S	3.04352000	0.50693800	0.41607200
O	3.61184100	-0.79947400	0.13634900
O	2.81122400	1.28991100	-0.78521500
O	3.70973300	1.23222000	1.47558100
O	0.78979400	-1.87492000	0.50750200

O	0.23058700	-2.16603300	1.77121200
O	-1.01201300	-2.75021300	1.54249700
O	-0.96988900	-3.31480900	-1.92466000
C	-1.82275300	-2.67954400	-1.38632500
H	-1.62897700	-1.99367600	1.45519000
H	-2.67920600	-3.10264300	-0.85146400
O	-1.77866600	-1.32093900	-1.42423800
S	-2.98831800	-0.46780000	-0.70456400
O	-4.19678100	-0.90517600	-1.35821000
O	-2.91111700	-0.84686900	0.69588800
O	-2.58085300	0.88285500	-0.98102000
O	6.07999100	-0.47801200	-1.37291900
H	5.24574200	-0.72285500	-0.93978600
H	5.89382700	0.38667800	-1.77413400
O	6.55600900	1.42359800	0.80779200
H	6.60504900	0.64642900	0.22662000
H	5.66470700	1.38010000	1.18927200
O	-6.40217000	0.87289200	-0.42781800
H	-5.83055500	0.24173800	-0.88979700
H	-5.81700600	1.62330100	-0.22761100
O	-4.34653100	2.58415600	0.73050300
H	-4.50268400	1.91878600	1.42193600
H	-3.62630600	2.21780200	0.19667400
O	-5.24753400	0.21263800	2.17233300
H	-4.48222800	-0.28633900	1.84959500
H	-5.84035600	0.26179200	1.40351800
O	5.36341200	2.35320000	-1.71064600
H	4.44904200	2.10965400	-1.49256500
H	5.82553800	2.33171100	-0.85652300

R6b:
IM4-R6b

C	-2.15854500	-1.20620900	-0.15214800
H	-1.53075900	-2.04190600	0.14696100
H	-2.59639700	-1.36751200	-1.13721400
O	-3.14494700	-1.06186900	0.84947500
S	-4.57108600	-0.47083500	0.40054700
O	-4.30243800	0.81487300	-0.21694800
O	-5.12140500	-1.43997600	-0.53035700
O	-5.27438700	-0.39270200	1.66146600
O	-1.39132100	-0.03569000	-0.28994900
O	-0.57497200	0.10269200	0.84839100
O	0.56790200	-0.63629600	0.63051800
O	1.39811400	0.11221100	-0.22563000
C	2.13737500	1.02438900	0.54868100
H	1.48717900	1.54857400	1.24476900
H	2.58095400	1.70671400	-0.17628000
O	3.11783100	0.36756700	1.32591200
S	4.56317200	0.14744100	0.65703700
O	4.33733600	-0.60359200	-0.56409300

O	5.08838800	1.47924500	0.41339700
O	5.26240300	-0.58627800	1.68856900
O	-6.77044200	1.69663000	-1.49746700
H	-5.87580400	1.47984100	-1.18836700
H	-7.20938600	0.83534400	-1.58889400
O	-7.75960400	1.10961300	1.20617100
H	-7.48258400	1.58394400	0.40498000
H	-6.94854200	0.68009300	1.52138900
O	6.92876800	-1.56735400	-1.51915700
H	6.00510300	-1.37583000	-1.28984000
H	7.40053000	-1.51967900	-0.67106100
O	8.10635600	-0.59126600	0.97911100
H	8.11083900	0.19302700	0.40609800
H	7.22273100	-0.59148900	1.38022900
O	7.50503600	1.29195600	-1.19800600
H	6.67726200	1.49166500	-0.73106000
H	7.35132600	0.41583300	-1.58838800
O	-7.96830800	-0.93316000	-0.87967900
H	-7.05684300	-1.25620500	-0.79183400
H	-8.11083100	-0.40380800	-0.07732700

TS-R6b

C	-2.34280400	-1.74611500	-0.69160000
H	-2.05076900	-2.79284900	-0.63769700
H	-2.78630300	-1.51702200	-1.66297000
O	-3.23560700	-1.51208500	0.37586700
S	-4.34436600	-0.36144100	0.17555600
O	-3.61325500	0.86375600	-0.08886800
O	-5.15304400	-0.79237000	-0.95049000
O	-5.03138400	-0.38600000	1.44680400
O	-1.22436500	-0.90848500	-0.63247400
O	-0.36726000	-1.36798700	0.51042300
O	0.11122000	-2.50612900	0.28722600
O	1.76664800	-1.94529000	-0.65541500
C	1.83931200	-0.73093000	-0.35816500
H	0.67418600	-0.53974700	0.12757300
H	1.79494900	0.03340500	-1.14629800
O	2.66377500	-0.37449200	0.74257700
S	4.14658000	0.12967700	0.37271500
O	4.75632900	-0.95540000	-0.37383000
O	3.96411600	1.33141600	-0.42203600
O	4.72734800	0.36163900	1.67582100
O	-5.52575400	2.93016700	-0.87034300
H	-4.78627400	2.32639400	-0.69323800
H	-6.22597600	2.35397100	-1.21784000
O	-6.84760700	1.92938800	1.54122000
H	-6.37008500	2.48543100	0.90306500
H	-6.26148500	1.16859700	1.67916000
O	7.61527500	-0.43585000	-0.67273500
H	6.70500600	-0.76596600	-0.60285100

H	7.70936800	0.15981300	0.08908200
O	7.26093500	1.79911500	1.19190100
H	7.04387700	2.17723000	0.32373800
H	6.42566200	1.41768700	1.50490700
O	6.49381900	2.09909500	-1.63287700
H	5.58163200	1.91058900	-1.35818700
H	6.93403500	1.23365100	-1.60372900
O	-7.57837700	0.82484800	-1.08595900
H	-6.84851700	0.18544900	-1.12020300
H	-7.61627000	1.09116700	-0.15276200

P5

C	-1.12186700	-1.99986200	2.06789300
H	-0.87896000	-2.23579000	3.10183100
H	-1.48871300	-2.86863700	1.52206600
O	-2.09174300	-0.97567400	2.12657900
S	-2.65152500	-0.35211500	0.75348900
O	-1.68207400	0.65194900	0.35039100
O	-2.75602800	-1.45811700	-0.17886600
O	-3.91987000	0.20252800	1.17435700
O	0.02126200	-1.61422900	1.35672000
O	0.65653400	-0.55851300	2.10664300
O	1.70833600	-1.07456700	2.85266000
O	-1.18391500	-0.37948200	-2.75003700
C	-0.27358700	0.06048400	-2.12582800
H	1.09328900	0.02030700	1.22552500
H	-0.08444500	1.11971700	-1.92864200
O	0.65602300	-0.80437800	-1.60502900
S	2.01514100	-0.21128900	-0.94415800
O	2.61839000	0.61531500	-1.94691900
O	1.54415100	0.60890200	0.19719700
O	2.70361200	-1.39889700	-0.54608400
O	-2.69012900	2.25655300	-1.86039000
H	-2.27756300	1.88292800	-1.06395500
H	-2.98424300	1.47017100	-2.34867800
O	-5.31692200	1.89328100	-0.69135600
H	-4.52054400	2.29782600	-1.07483900
H	-4.96848200	1.37847700	0.05458200
O	4.75901700	1.73419600	-0.13941300
H	4.15092000	1.72947200	-0.89007500
H	5.18586700	0.86040900	-0.16663700
O	5.55607900	-1.02467100	0.30627700
H	5.08224300	-0.75859900	1.11917800
H	4.85910100	-1.40213800	-0.24677200
O	4.03973000	0.22850200	2.29616400
H	3.16457400	-0.20319200	2.43264300
H	3.91120800	0.90227300	1.61165600
O	-4.26512900	-0.21000300	-2.44339200
H	-3.75111400	-0.74491800	-1.81874100
H	-4.81862500	0.36178200	-1.88604400

R7:

IM4-R7

C	-2.42789600	-2.43039500	-0.67125700
H	-1.96319100	-3.27620500	-1.17884200
H	-3.43446100	-2.66889600	-0.33721500
O	-1.69687900	-2.02592700	0.46806600
S	-0.16876500	-2.52664000	0.57965800
O	0.50378000	-2.05277300	-0.61561600
O	0.26059600	-1.85509200	1.79394800
O	-0.22957800	-3.96517700	0.67963600
O	-2.47141600	-1.41730900	-1.64315200
O	-3.37598300	-0.42491200	-1.20295100
O	-2.64885600	0.63621600	-0.73578000
O	-2.31054900	1.41213000	-1.86981800
C	-0.91773500	1.36395600	-2.07535500
H	-0.77596100	1.51156100	-3.14331400
H	-0.51640500	0.41226800	-1.73111800
O	-0.25996500	2.45113100	-1.45392800
S	0.02807000	2.38569300	0.12849800
O	0.48349900	1.02730400	0.38010700
O	1.05327000	3.39103000	0.29133600
O	-1.22524800	2.68972300	0.78743100
O	0.86714700	1.90191300	3.67516900
H	1.59998200	1.84172900	3.03826700
H	0.14769600	1.35549000	3.31651600
O	-0.04859800	-1.72308700	-3.40049300
H	0.11772100	-1.83926900	-2.45038700
H	-0.92241400	-1.31604200	-3.43354400
O	2.53085400	0.17426200	-1.40630800
H	3.11927500	0.93941100	-1.40525100
H	1.76357600	0.45352900	-0.87578900
O	3.09247800	1.88572700	1.84638500
H	3.04541900	0.93743000	1.61440800
H	2.57819500	2.34617100	1.16630000
O	2.97006200	-0.87198000	1.24245400
H	2.94308800	-0.71453200	0.28258800
H	2.06343800	-1.13450800	1.47231000
O	-1.39066200	0.39944000	2.65966200
H	-1.51792400	1.04190300	1.94423900
H	-0.86565700	-0.31527100	2.26314300

TS-R7

C	-1.96371800	-2.19417000	-0.73485200
H	-1.60245400	-3.14620800	-1.15119400
H	-2.99648200	-2.31775300	-0.40490900
O	-1.22116200	-1.83499100	0.43219900
S	0.30824000	-2.30998800	0.50391400
O	0.96250700	-1.77158200	-0.67725100
O	0.74830300	-1.68389600	1.74013400

O	0.28217600	-3.75402400	0.53873400
O	-1.85250100	-1.27815200	-1.73187300
O	-2.93526400	0.15745100	-1.09695800
O	-2.17295000	1.07546000	-0.70380100
O	-1.86866900	1.94058500	-1.82193300
C	-0.47423400	1.92940000	-2.01564300
H	-0.34020000	2.18877800	-3.06330100
H	-0.06676600	0.94524400	-1.78451100
O	0.17995300	2.94301300	-1.28227100
S	0.51095800	2.70803600	0.27728700
O	0.95399800	1.32585000	0.37690300
O	1.55325400	3.68031900	0.51171500
O	-0.72012400	2.95593300	0.99772900
O	1.37560900	1.99523900	3.79640600
H	2.10846900	1.96729600	3.15708200
H	0.65731600	1.46496300	3.41182100
O	0.33588900	-2.71671400	-3.29259400
H	0.54750800	-2.38917300	-2.40204400
H	-0.55196600	-2.38446500	-3.46815800
O	3.05962500	0.56449600	-1.36833600
H	3.68806800	1.29244400	-1.28637700
H	2.28100300	0.85147200	-0.85888700
O	3.58096900	2.06448800	1.95473600
H	3.52206400	1.13098800	1.67068200
H	3.06898000	2.56898600	1.30501000
O	3.42965800	-0.65155900	1.20961200
H	3.39502200	-0.43755100	0.26067900
H	2.52683600	-0.93377500	1.43149900
O	-0.87272700	0.54380500	2.70770500
H	-0.98689500	1.21909500	2.02088200
H	-0.36525500	-0.16807300	2.28397800

IM5

C	-2.31307800	-2.17295100	-1.00045800
H	-2.21730000	-3.25103300	-1.21473200
H	-3.36934100	-1.97933500	-0.76230600
O	-1.57734300	-1.82489200	0.17215100
S	-0.13842800	-2.50617600	0.36679200
O	0.66295800	-2.09675300	-0.77507800
O	0.29605200	-1.91121500	1.61910000
O	-0.37042400	-3.93072400	0.42313100
O	-1.97296300	-1.48192100	-2.09120000
O	-3.83767300	0.90490500	-0.12558100
O	-2.67955300	0.79730000	-0.54193100
O	-2.49521500	1.66217900	-1.71579900
C	-1.11887000	1.67394500	-1.98652800
H	-1.04052300	1.91208200	-3.04470700
H	-0.67929600	0.70278800	-1.75848500
O	-0.44436300	2.71043900	-1.30125300
S	-0.01278600	2.48979300	0.23489300

O	0.43621900	1.10817400	0.31869400
O	1.04326400	3.46284200	0.39610900
O	-1.19445900	2.74595000	1.03172900
O	0.91422500	1.73632800	3.75263200
H	1.63324700	1.74508100	3.09705300
H	0.19796400	1.20519100	3.36577200
O	0.02233600	-3.53464600	-3.16984000
H	0.27115500	-3.02116300	-2.38301600
H	-0.64301900	-2.99580500	-3.61268900
O	2.60218400	0.46455600	-1.39945800
H	3.21077600	1.20130000	-1.26381500
H	1.80688900	0.70482600	-0.89139100
O	3.07942400	1.88778000	1.87388100
H	3.03321700	0.95122800	1.59785100
H	2.56358100	2.38024000	1.21786800
O	2.96696900	-0.83485100	1.14388500
H	2.94259100	-0.61066300	0.19735900
H	2.06737400	-1.14149600	1.34612500
O	-1.34112900	0.28511100	2.66693100
H	-1.46452900	0.97698400	1.99825500
H	-0.84013100	-0.41539600	2.21805500

R8

IM5-R8

C	-2.29379700	-2.19126900	-1.00764700
H	-2.18909300	-3.27044500	-1.21200000
H	-3.35395900	-2.00220500	-0.78338400
O	-1.57404400	-1.82911500	0.17049700
S	-0.13329600	-2.49962600	0.38789700
O	0.67984200	-2.09289800	-0.74658600
O	0.28085300	-1.89320700	1.64160700
O	-0.35686300	-3.92522800	0.45119200
O	-1.94527900	-1.50698600	-2.09993900
O	-3.84201300	0.88887300	-0.18799400
O	-2.67745000	0.78281300	-0.58645100
O	-2.48120900	1.63438900	-1.76797800
C	-1.10200500	1.64486700	-2.02370800
H	-1.01232300	1.87065900	-3.08368800
H	-0.66362500	0.67720500	-1.77930000
O	-0.43631900	2.69038000	-1.34347400
S	-0.02327500	2.48927000	0.20029400
O	0.42662100	1.10942100	0.30713700
O	1.02945000	3.46586100	0.36221200
O	-1.21464200	2.75341100	0.97981300
O	0.85891200	1.76751500	3.75018400
H	1.58614900	1.78123100	3.10378300
H	0.14957400	1.23496900	3.35261600
O	0.07443500	-3.55565900	-3.13906700
H	0.31128400	-3.03547300	-2.35308100
H	-0.59247600	-3.02593100	-3.59040200

O	2.63537900	0.45417200	-1.35204700
H	3.23495500	1.19805400	-1.21520700
H	1.82826700	0.69492700	-0.86308800
O	3.04435100	1.92243200	1.89652400
H	3.00499200	0.98189900	1.63348200
H	2.53418100	2.40386900	1.22791900
O	2.95529100	-0.81301000	1.20781200
H	2.95077000	-0.59900700	0.25853000
H	2.05244300	-1.12016200	1.39421400
O	-1.37978300	0.30939400	2.64043400
H	-1.49669100	0.99648400	1.96567100
H	-0.86960400	-0.39187500	2.20322100

TS-R8

C	-2.04523800	-2.29377600	-1.05584600
H	-1.90227400	-3.36261700	-1.28955300
H	-3.11128000	-2.15018700	-0.82546300
O	-1.33612500	-1.93638400	0.13016200
S	0.11960800	-2.57784400	0.33652400
O	0.91609500	-2.15200800	-0.80262800
O	0.52937800	-1.96651500	1.58920500
O	-0.07533900	-4.00773700	0.39765900
O	-1.72488600	-1.56686800	-2.12912100
O	-3.56359100	0.79077200	-0.08015100
O	-2.46623800	0.65437800	-0.51765900
O	-2.22135600	1.67762600	-1.85033700
C	-0.87107900	1.61805400	-2.09778300
H	-0.73347600	1.82784400	-3.16055800
H	-0.46316200	0.63475500	-1.84874600
O	-0.13086400	2.63679300	-1.42071400
S	0.25787900	2.41676700	0.11648900
O	0.68704200	1.02854100	0.22265000
O	1.32574300	3.37423200	0.30580500
O	-0.93408800	2.68869000	0.89572100
O	1.12791400	1.61766200	3.78277200
H	1.84640600	1.66522300	3.12808400
H	0.40620000	1.12432800	3.35760800
O	0.33977700	-3.60018100	-3.20975400
H	0.56126500	-3.08346500	-2.41702500
H	-0.39885000	-3.12827700	-3.61086900
O	2.91168500	0.35807800	-1.39148700
H	3.50417900	1.10853400	-1.25989500
H	2.09390000	0.60423100	-0.92227000
O	3.27757700	1.83460500	1.89458900
H	3.24556900	0.89645900	1.62235400
H	2.76128100	2.31808500	1.23149000
O	3.20628300	-0.89219200	1.17268000
H	3.20835700	-0.66905400	0.22513700
H	2.30128300	-1.19762400	1.35112300
O	-1.11615500	0.25808800	2.57080700

H	-1.20317000	0.96095400	1.90731100
H	-0.60844300	-0.44409100	2.13217100

IM6-R8

C	-2.30030800	-2.23621000	-1.03750400
H	-2.09991000	-3.28774900	-1.30441900
H	-3.36793300	-2.16665100	-0.77856900
O	-1.58123900	-1.86725900	0.13913200
S	-0.11312400	-2.49028800	0.32220700
O	0.66830000	-2.01785700	-0.80899700
O	0.29179500	-1.90766500	1.58992000
O	-0.28363600	-3.92442900	0.34422500
O	-2.06102200	-1.45754000	-2.09370100
O	-4.08898600	1.15127800	0.62797800
O	-3.40274200	0.47953900	-0.06891600
O	-2.27281500	1.89620200	-2.14159000
C	-0.97220700	1.59939200	-2.08721400
H	-0.58759300	1.51002000	-3.11481200
H	-0.83441700	0.61947100	-1.59987000
O	-0.16994300	2.59664200	-1.44954500
S	0.02789900	2.45665000	0.13502700
O	0.54535700	1.11041300	0.33986900
O	0.98609400	3.49956200	0.42175600
O	-1.27873400	2.64193300	0.73463700
O	0.75111000	1.88735100	3.69485800
H	1.52296500	1.86532100	3.10349300
H	0.08149300	1.31110300	3.28812200
O	0.08946800	-3.36000700	-3.27664700
H	0.29985400	-2.86741500	-2.46582400
H	-0.70991600	-2.94262200	-3.61725600
O	2.72609100	0.40446500	-1.32798900
H	3.33211200	1.14730800	-1.21685200
H	1.92125900	0.66844300	-0.84679800
O	3.04250400	1.94332300	1.94640900
H	3.00155000	1.00031500	1.69365300
H	2.52985800	2.41480900	1.27290800
O	2.97253900	-0.80832200	1.26579700
H	3.00592500	-0.59775700	0.31628500
H	2.06427500	-1.11957200	1.41532500
O	-1.37455600	0.30480700	2.56675200
H	-1.51411300	0.94793200	1.85352700
H	-0.84725400	-0.40493500	2.16462200

R9a[6]: •OCH_2-O-SO_3^- + 6 H_2O → •CHO + HSO_4^- + 6 H_2O

IM6-R9a[6]

C	1.26391100	1.56112500	-0.70689700
H	2.34571100	1.78611300	-0.70414300
H	0.98424500	1.29787900	-1.74419000
O	0.59488000	2.64714100	-0.34495300
O	0.99099000	0.47043100	0.18093200

S	1.42874600	-0.99187100	-0.31073400
O	0.67052900	-1.23987500	-1.52331400
O	2.85930900	-0.92326500	-0.53999400
O	1.04288900	-1.81514700	0.81435800
O	4.20302900	0.90833900	1.23131200
H	4.16865800	1.76839600	0.79810800
H	3.79629800	0.29392800	0.59924000
O	-3.26699600	-1.62153900	-0.25684500
H	-2.71645400	-1.53804700	0.54201900
H	-2.79151700	-1.08366300	-0.91520200
O	-4.57392400	0.91377500	0.19970600
H	-3.73146400	1.35353900	0.39207400
H	-4.31273400	-0.01162600	0.05740200
O	-1.87669700	1.57447900	0.88880500
H	-1.04377700	2.05705700	0.79198300
H	-1.64908500	0.72319800	1.31310000
O	-1.46287000	-1.00877900	1.85224800
H	-1.57894700	-1.19950600	2.79057100
H	-0.58326400	-1.35086900	1.61074200
O	-1.75986900	0.31783000	-1.71875800
H	-1.76737900	0.81285400	-0.87907700
H	-0.93384700	-0.19317500	-1.70748800

TS1-R9a[6]

C	-1.30652800	1.54056400	0.35080000
H	-2.75485500	1.60812400	-0.02818000
H	-1.37244400	1.31174100	1.42583400
O	-0.70555800	2.57206700	-0.06349300
O	-0.92656800	0.32396000	-0.39422300
S	-1.39699800	-1.09418900	0.13686800
O	-0.90512700	-1.20905100	1.49884400
O	-2.85483100	-1.10728600	0.06815100
O	-0.77405600	-2.01054100	-0.79718000
O	-3.80317000	1.31961100	-0.41583500
H	-4.46782600	1.65912300	0.20788900
H	-3.75468600	0.33391400	-0.30654300
O	3.23001700	-1.54336100	0.47972500
H	2.78173700	-1.47258500	-0.38171000
H	2.65868400	-1.02367700	1.07476300
O	4.44294600	1.05711900	0.13180200
H	3.59917100	1.45225100	-0.14006500
H	4.21795500	0.12045900	0.26128400
O	1.82152400	1.65384300	-0.82247200
H	0.93532600	2.04225900	-0.69222000
H	1.67956100	0.79394300	-1.26206300
O	1.65679600	-0.97503700	-1.81487800
H	1.81953700	-1.19414600	-2.73980900
H	0.80045400	-1.37927500	-1.58507100
O	1.53826400	0.33250600	1.77801900
H	1.59575000	0.84183400	0.94971100

H	0.69088200	-0.14096700	1.73380500

P-R9a^6-step1

C	-1.23349300	1.64579200	0.29124000
H	-3.11546400	1.63879600	0.00679100
H	-1.13195800	1.44403200	1.36575300
O	-0.68494200	2.63422300	-0.26826900
O	-0.93123900	0.35547800	-0.45260400
S	-1.39155200	-1.02421100	0.11895500
O	-1.05186300	-1.05412900	1.52781200
O	-2.85581400	-1.09764200	-0.08691900
O	-0.69981400	-2.00256100	-0.69093900
O	-3.93658400	1.09946400	-0.24540400
H	-4.62302000	1.25205500	0.42667000
H	-3.56512900	0.09574400	-0.17142100
O	3.21674900	-1.60615100	0.43547600
H	2.78953400	-1.50130100	-0.43271600
H	2.63564600	-1.10192000	1.03550800
O	4.48472200	0.97868700	0.20972500
H	3.65451500	1.40970000	-0.04810800
H	4.23232900	0.04360300	0.29423500
O	1.86876600	1.64734900	-0.70864100
H	0.99110700	2.07567400	-0.64601100
H	1.72978300	0.81758300	-1.20311600
O	1.67178400	-0.92406400	-1.85042000
H	1.80316600	-1.12185300	-2.78506300
H	0.82538400	-1.33476500	-1.60054500
O	1.56236000	0.23758800	1.79576900
H	1.61872600	0.78974800	0.99333100
H	0.66816800	-0.14039900	1.78929400

R-R9a^6-step2

C	-1.20219300	1.65805500	0.11447500
H	-3.12765200	1.62426400	0.17865700
H	-0.99463400	1.51244000	1.18315700
O	-0.72455500	2.62222000	-0.54147500
O	-0.92907400	0.33349100	-0.58686700
S	-1.40723300	-1.01134100	0.04266100
O	-1.07776000	-0.97854500	1.45436200
O	-2.87285800	-1.08107600	-0.16759600
O	-0.72806300	-2.03597500	-0.71835900
O	-3.97983200	1.08017500	0.13897600
H	-4.43978300	1.17415100	0.99095800
H	-3.58736800	0.07744500	0.03878300
O	3.25375900	-1.57504600	0.62470500
H	2.84521600	-1.53468000	-0.25766000
H	2.64767200	-1.04395900	1.17532400
O	4.48500200	1.01402300	0.26554700
H	3.66074200	1.42201800	-0.04282400
H	4.23594500	0.08458800	0.40496100

O	1.88146300	1.60773500	-0.75154000
H	1.01246500	2.05293900	-0.78408900
H	1.76125900	0.74722900	-1.19634900
O	1.75281900	-1.03819000	-1.72833900
H	1.92759000	-1.26541000	-2.64917600
H	0.88998600	-1.43291100	-1.51103200
O	1.52142000	0.31014900	1.80416100
H	1.60120800	0.83394700	0.98458100
H	0.63767200	-0.09060800	1.76667400

TS2-R9a[6]

C	-1.18228300	1.69689500	0.11191900
H	-3.31252100	1.63007100	0.21691300
H	-0.94816800	1.55483900	1.17528100
O	-0.71721400	2.64610200	-0.56692500
O	-0.87786700	0.33956900	-0.57908500
S	-1.38813500	-0.98208800	0.04036700
O	-1.16289700	-0.93094900	1.46963300
O	-2.85253200	-1.05686000	-0.26294700
O	-0.69376200	-2.03574600	-0.66033200
O	-4.05729000	0.98221800	0.16051500
H	-4.51634200	0.99147000	1.01588900
H	-3.51507400	-0.03599900	-0.00929800
O	3.24587500	-1.60196600	0.57247900
H	2.84465900	-1.53245900	-0.31135200
H	2.63427800	-1.09158700	1.13655500
O	4.49248000	0.98784700	0.31123800
H	3.67306500	1.41275900	0.01321100
H	4.23665000	0.05517500	0.41263700
O	1.89440100	1.61954100	-0.69192900
H	1.03602700	2.08256900	-0.74559000
H	1.77443300	0.77649700	-1.16882700
O	1.75548100	-0.99362500	-1.76860500
H	1.91889600	-1.19989200	-2.69645700
H	0.90067200	-1.40105000	-1.54717200
O	1.51960800	0.24413400	1.81291500
H	1.59163300	0.79520700	1.01028700
H	0.61538900	-0.10768700	1.80486600

P6

C	-1.37943500	2.41271300	0.18947100
H	-3.45446100	1.40001800	0.28795400
H	-0.67865000	1.93336100	0.91045600
O	-1.10235800	3.19121800	-0.64188800
O	-1.03023900	0.04197100	-0.96534400
S	-1.25244400	-1.04867200	-0.02882700
O	-1.11701000	-0.62306800	1.36142400
O	-2.74474400	-1.50794600	-0.20540000
O	-0.47532000	-2.23748000	-0.34353300
O	-4.10546400	0.67869400	0.28084300

H	-4.48013300	0.66796700	1.17112400
H	-3.33828500	-0.71586400	-0.01869300
O	3.25956000	-1.42682000	0.88900500
H	2.89166400	-1.51726300	-0.00719600
H	2.61702800	-0.84756000	1.34177500
O	4.50126800	1.05721500	0.13874800
H	3.68919300	1.42534400	-0.24119400
H	4.23044200	0.16938800	0.43013100
O	1.84823800	1.45832000	-0.86053900
H	1.10707000	1.97304100	-1.20240900
H	1.75048700	0.55776000	-1.22975400
O	1.86304800	-1.24596100	-1.57955400
H	2.04859100	-1.55177800	-2.47504000
H	1.03022100	-1.67427100	-1.30726000
O	1.44911300	0.53606400	1.79927600
H	1.56850600	0.97346300	0.93487600
H	0.56531200	0.13120600	1.74534200

R9b[6]: $\bullet OCH_2\text{-}O\text{-}SO_3^- + 6\ H_2O \rightarrow \bullet CHO + HSO_4^- + 6\ H_2O$

IM6-R9b[6]

C	0.63353700	-0.03711700	-1.70778400
H	-0.22690000	0.56229300	-2.04338500
H	1.54127300	0.45655600	-2.09395300
O	0.53889700	-1.25830400	-2.24066500
O	0.60914800	-0.02662300	-0.28049200
S	2.02165900	0.04231900	0.48386900
O	2.75805600	-1.14129300	0.08519700
O	2.64305100	1.27614400	0.03788800
O	1.60685800	0.04752800	1.87043700
O	0.62455600	3.01537000	-1.04756600
H	0.65300800	3.90411800	-0.67413200
H	1.36723900	2.54173100	-0.63636500
O	-1.53162100	-2.15366000	1.25644800
H	-1.36164900	-1.28907100	1.67008800
H	-0.67065400	-2.47643800	0.93152400
O	-2.43873100	-0.81388600	-1.09121900
H	-2.21697800	0.11033900	-0.87945400
H	-2.12902600	-1.32274600	-0.31982700
O	-1.83294500	1.85836400	-0.21478600
H	-0.98806500	2.23379200	-0.52686000
H	-1.62497700	1.47424200	0.65668100
O	-1.15682800	0.51348500	2.18227800
H	-1.42160700	0.79825500	3.06491600
H	-0.18367100	0.47063400	2.19294200
O	0.76164600	-3.22803300	-0.01167700
H	0.47232100	-2.85473700	-0.85724600
H	1.55240300	-2.70326400	0.19314200

TS-R9b[6]

C	0.08936400	-0.15049000	-1.73387600

H	-0.58439000	0.66314700	-2.02919600
H	1.38072900	0.48511900	-1.61834300
O	0.08655200	-1.26898900	-2.28485800
O	0.04398900	-0.18372600	-0.20818900
S	1.41177900	0.17573700	0.45438900
O	2.07507200	-1.03398000	0.86008100
O	2.12742600	0.82659800	-0.72076700
O	1.15855100	1.12318700	1.50587300
O	0.13369500	3.15365800	-1.05363000
H	0.21921100	3.91204200	-0.46288200
H	0.98518600	2.70007200	-0.99805900
O	-1.98228900	-2.17176600	1.10424700
H	-1.73084500	-1.34434200	1.55114000
H	-1.16360900	-2.51776300	0.70265600
O	-3.04937000	-0.62780300	-1.05813000
H	-2.74143500	0.26516000	-0.82105400
H	-2.67989400	-1.20426400	-0.36515400
O	-2.23538600	1.92815500	-0.06506200
H	-1.38492000	2.26306900	-0.40387300
H	-2.02119600	1.48480600	0.77488900
O	-1.56210600	0.41208100	2.26860000
H	-1.96611000	0.58932500	3.12674800
H	-0.64317700	0.71365800	2.33911300
O	0.33519600	-3.21536100	-0.19949700
H	0.24173600	-2.65238900	-0.98912600
H	1.02446900	-2.77012200	0.31420700

P6

C	0.85165900	-0.20317900	-2.54750100
H	-0.03399100	0.46853400	-2.47786700
H	2.70618100	0.78839800	-1.15712400
O	0.85376900	-1.31914100	-2.90775700
O	0.49363300	-0.08256700	0.06325000
S	1.83246100	-0.01597300	0.61731900
O	2.57691200	-1.25115600	0.44224100
O	2.61211300	1.09334300	-0.23482200
O	1.87280500	0.53129500	1.95762400
O	0.30297500	2.85615400	-1.20384100
H	0.42760100	3.76644300	-0.90934600
H	1.07389800	2.37668600	-0.86797200
O	-1.55024400	-2.06767100	1.39047600
H	-1.29924500	-1.20531600	1.76572500
H	-0.75346500	-2.41185600	0.94737100
O	-2.33289300	-0.82842200	-1.10237900
H	-2.21529400	0.09859100	-0.82366700
H	-2.10817000	-1.33393000	-0.30179800
O	-1.99266000	1.76468900	0.03503800
H	-1.19206500	2.13561400	-0.38170600
H	-1.68556600	1.39806800	0.88322000
O	-1.01424900	0.55860400	2.45665600

H	-1.25741900	0.80124700	3.35789100
H	-0.04700100	0.64367600	2.40752800
O	0.51590300	-3.16708800	-0.25373700
H	0.09909600	-2.83016500	-1.05724700
H	1.26325500	-2.56487900	-0.09757700

R9a[22]: •OCH_2-O-SO_3^- + 22 H_2O → •CHO + SO_4^{2-} + H^+ + 22 H_2O

IM6-R9a[22]

C	-0.84755600	1.03755000	-1.98338400
H	0.05968300	0.78264400	-2.58023800
H	-1.73701900	0.86621700	-2.62142600
O	-0.79181100	2.31458800	-1.62764800
O	-0.97946600	0.12671800	-0.90153000
S	0.34566100	-0.19111700	-0.01678300
O	-0.20151900	-1.02265400	1.03954800
O	1.30690000	-0.83902300	-0.92535900
O	0.82678000	1.14463900	0.40811900
O	-0.61565700	-1.74186500	-3.15310500
H	-1.52359900	-1.45649700	-2.93804400
H	-0.22541600	-1.08136000	-3.73968700
O	-0.52092400	-3.60901800	1.92527300
H	0.40210000	-3.89827700	1.81501500
H	-0.47839900	-2.76538200	1.44177400
O	1.78339800	2.75769800	2.61444700
H	1.27843100	3.36490900	2.03909700
H	1.25794500	1.94185600	2.56654300
O	-3.08106800	-1.08422700	1.32447500
H	-2.69664000	-0.18787200	1.42925300
H	-2.48562600	-1.54436300	0.70327700
O	2.66544000	1.75983800	-1.60691700
H	1.98006500	1.61724200	-0.92384900
H	3.26516100	0.99456800	-1.46032900
O	-2.69974700	3.87313300	0.50901700
H	-2.68546100	3.28224000	1.27810100
H	-1.75457800	4.09894300	0.42706800
O	1.35477800	4.20216000	-1.81211300
H	0.58887800	3.80755100	-2.25050800
H	2.04641500	3.52309700	-1.89164600
O	0.48589100	-3.46034900	-1.30173000
H	0.92022100	-3.08494700	-0.51130600
H	0.30243100	-2.71455600	-1.90690600
O	-1.96007400	1.42762800	1.63264100
H	-1.08977500	1.21242200	2.02053100
H	-1.87315700	1.44246700	0.66469600
O	0.15889300	0.46651300	3.27575700
H	-0.41836900	-0.35763000	3.34134700
H	0.15804600	0.83896500	4.16593400
O	-2.03893000	-3.07806400	-0.39077700
H	-2.07781500	-3.63518400	0.40032700
H	-1.15236000	-3.30736200	-0.76406900

O	1.64422900	-0.08209000	-3.63826900
H	2.09656100	0.72096000	-3.33860900
H	1.61504700	-0.60206300	-2.81721100
O	-3.19699200	-1.13312000	-2.01909200
H	-3.03764600	-0.35074400	-1.46322700
H	-2.94059300	-1.88352100	-1.44653400
O	3.49427100	-3.31944600	-1.27943600
H	3.75943300	-2.39074600	-1.36146000
H	2.60091300	-3.37575800	-1.65446900
O	2.02163500	-3.13813500	1.03726600
H	2.74572300	-3.35054600	0.41796300
H	2.23829400	-2.25385000	1.39649000
O	0.14386000	3.87064600	0.64257200
H	0.62797100	4.16207400	-0.16019600
H	0.04667900	2.91712200	0.47905900
O	-5.27600600	-0.22743700	-0.19846500
H	-5.03699400	-0.67436200	-1.02416000
H	-4.65673000	-0.62822600	0.44347900
O	2.53657400	-0.59073300	1.99407900
H	2.98100800	0.23353300	1.71224300
H	1.73610500	-0.29325200	2.46438200
O	3.96461900	-0.47021800	-0.65385500
H	3.08615600	-0.72706500	-0.31548700
H	4.42526100	-0.12580800	0.12622400
O	-3.36444900	1.60462700	-0.94807200
H	-4.18945100	1.23940400	-0.57346600
H	-3.24502800	2.50555000	-0.58456900
O	3.80847300	1.74433800	1.17180600
H	3.54232600	2.04577900	0.29202500
H	3.19289400	2.23320600	1.77338400
O	-1.43751900	-1.59072700	3.58873600
H	-2.15809900	-1.47285300	2.93854900
H	-1.02067300	-2.42037500	3.28343500

TS1-R9a[22]

C	0.74901600	1.45369900	-1.84192500
H	0.35949000	1.04370800	-2.80619700
H	0.69463100	2.58211900	-1.98169700
O	1.96660200	1.06282500	-1.63004900
O	-0.21066400	1.19207500	-0.80535300
S	-0.04040500	-0.13475800	0.14636900
O	-1.34679100	-0.23675400	0.77973100
O	0.25259100	-1.24455500	-0.79217500
O	1.08869200	0.17158600	1.02813100
O	-1.29942100	-0.88977500	-3.71154000
H	-1.57831300	0.00714500	-3.44551200
H	-0.32398300	-0.91336800	-3.66570300
O	-3.67478400	-1.93499300	1.52420300
H	-2.89687000	-2.43005100	1.19130000
H	-3.87380100	-1.30186200	0.81195100

O	2.39366000	0.69577500	3.42537400
H	2.71475800	1.24952100	2.69688100
H	1.47760500	0.49052700	3.16807200
O	-2.96825300	2.09719600	0.92362300
H	-2.02027300	2.08105400	1.20218300
H	-3.10281700	1.31003900	0.36456700
O	3.46085800	-1.25909200	-0.76520700
H	2.71747800	-0.65178100	-0.91217000
H	3.04205900	-2.14465000	-0.77150300
O	1.64134300	3.31121500	0.68022500
H	1.90864800	3.97839500	1.32858500
H	2.39394400	2.66984400	0.66072200
O	4.83860300	0.98456500	-1.57009800
H	4.07788300	1.30693200	-2.07641400
H	4.62061000	0.04184400	-1.45213700
O	-2.53068000	-1.94687600	-1.54855000
H	-1.93021700	-2.05623300	-0.79079800
H	-1.98500500	-1.56443600	-2.27673400
O	-0.55858100	2.40228200	2.07542500
H	-0.36692000	1.57123600	2.54770500
H	0.23877100	2.57972000	1.53473800
O	-0.43121100	-0.01827800	3.54769500
H	-1.41521400	0.03171000	3.37914000
H	-0.34430400	0.04312700	4.50993800
O	-4.06153600	-0.04189500	-0.63731000
H	-4.97459600	-0.19016700	-0.92120200
H	-3.53049900	-0.78676600	-1.04541900
O	1.42197900	-1.43988700	-3.27797000
H	2.13096800	-0.78131200	-3.24759100
H	1.10556000	-1.46716100	-2.35635300
O	-2.45252000	1.37064200	-2.46191500
H	-1.78065200	1.31486500	-1.75815500
H	-3.22822600	0.97719600	-2.01686100
O	-0.34225900	-4.09975500	-1.80473100
H	0.54474300	-4.01435400	-1.40761300
H	-0.51675800	-3.21298000	-2.15209200
O	-1.43144800	-3.36097800	0.70901000
H	-1.06779900	-3.71597200	-0.12543900
H	-0.66373300	-2.98738100	1.19844900
O	3.53625000	1.40600800	0.83727700
H	4.06852000	1.30971200	0.01666100
H	2.80726700	0.76804800	0.72898000
O	-2.42918200	3.86592200	-1.16244900
H	-2.52624200	3.17895100	-1.84654000
H	-2.65865800	3.36305200	-0.35289400
O	0.51000300	-2.35007900	2.31010500
H	1.45239500	-2.13120600	2.14095100
H	0.15641900	-1.53168600	2.71684600
O	1.85674800	-3.40896300	-0.25746400
H	1.23195400	-2.67877200	-0.08183200

H	2.02616100	-3.80492000	0.61028400
O	0.26602300	4.35240900	-1.50040300
H	-0.67954500	4.16679600	-1.30351600
H	0.74383500	4.08140500	-0.69224200
O	3.17994300	-1.66619200	2.05877200
H	3.32443900	-1.41429700	1.12783200
H	3.00741400	-0.82051800	2.51895000
O	-3.03873600	0.18767900	3.11364800
H	-3.09635500	0.89669400	2.44655000
H	-3.23023100	-0.62668600	2.59093200

P-R9a[22]-step1

C	1.20219000	-0.25903700	-2.38030100
H	0.37378200	-0.71709400	-2.95367300
H	1.50784800	1.67632700	-3.28709400
O	2.20156000	-0.94825100	-1.99831900
O	0.61586300	0.65512000	-1.40502000
S	0.14111900	0.12373600	0.04164200
O	-1.13642900	0.82038600	0.29691900
O	-0.01575900	-1.34200500	-0.05375200
O	1.21168700	0.53023100	0.96235500
O	-1.73849600	-2.62789400	-3.09578400
H	-1.66030100	-1.67805100	-3.27256100
H	-0.83805400	-2.92139600	-2.85396800
O	-3.32867700	-0.08689000	1.79750700
H	-3.23541600	-1.02527900	2.03532800
H	-2.51408900	0.08737700	1.28557900
O	2.50952200	1.80139500	2.96465000
H	3.11629300	1.79539800	2.20715300
H	1.64245900	1.71109000	2.53516000
O	-3.31896000	2.28388300	-0.59857300
H	-2.53075700	1.73075400	-0.45075900
H	-4.03437000	1.60783400	-0.67778000
O	3.09403800	-1.84588900	0.40403400
H	2.58623500	-1.53424400	-0.37702200
H	2.57071900	-2.58447600	0.76136600
O	2.48708300	3.10590700	-0.88843500
H	1.59992700	3.21301500	-0.32643000
H	3.03312600	2.31929500	-0.48505300
O	4.83271000	-0.59211700	-1.27818700
H	4.01971900	-0.64653400	-1.82093000
H	4.66485800	-1.30331600	-0.63633600
O	-2.88001100	-1.65465000	-0.83462600
H	-2.50481900	-1.91809300	0.04016100
H	-2.54698100	-2.28562000	-1.50860000
O	0.30674900	3.36318000	0.24289500
H	0.08299800	2.86787900	1.05988400
H	-0.29785600	3.05020200	-0.46394100
O	-0.47601700	2.21497900	2.66101700
H	-1.44315200	2.38298200	2.44715100

H	-0.29061600	2.76079800	3.43466000
O	-4.92085200	0.12195000	-0.47537100
H	-4.66047300	0.09084300	0.46433300
H	-4.39514600	-0.63398600	-0.80345000
O	0.63725100	-3.25371900	-1.82114800
H	1.46084900	-2.83696400	-2.12991300
H	0.32157000	-2.56814200	-1.19802300
O	-2.06609900	0.22488000	-2.49580400
H	-2.23889400	-0.35343200	-1.70619000
H	-2.95507000	0.49795200	-2.75699800
O	-0.65302800	-4.58531700	0.41942600
H	0.07559100	-4.41898300	1.04079600
H	-0.24735900	-4.41276000	-0.44539900
O	-2.08237900	-2.50743600	1.62525200
H	-1.65151800	-3.30693900	1.25657100
H	-1.37704100	-1.98278100	2.05989800
O	3.66726200	1.21479900	0.24697200
H	4.25468800	0.61486500	-0.29316100
H	2.86780400	0.67887700	0.42912000
O	-1.06090700	2.76677600	-2.08141300
H	-1.19828700	1.79533100	-2.15724000
H	-1.96145300	3.09146000	-1.92078400
O	-0.42508200	-0.69057500	2.91541500
H	0.54814900	-0.75810500	2.96615800
H	-0.56746900	0.26749900	2.81341500
O	1.08774500	-3.08322300	1.86117600
H	0.62898700	-2.36977400	1.37625000
H	1.42623800	-2.64546100	2.65929700
O	1.48591700	2.64952100	-3.18910800
H	0.54979500	2.83049400	-2.96874500
H	2.17164700	2.89579400	-1.84025400
O	2.41801300	-0.98158300	3.05179100
H	2.70451700	-1.04860100	2.12065200
H	2.53504800	-0.03068300	3.25037200
O	-3.01811500	2.59548000	2.16158500
H	-3.19517800	2.70183300	1.20335200
H	-3.34629700	1.69574600	2.33956000

R-R9a[22]-step2

C	1.00718500	0.51806800	-2.25227300
H	0.30684900	0.25893000	-3.06854400
H	1.23019600	2.58015800	-2.80992100
O	2.02379900	-0.20395000	-1.99045300
O	0.20026700	0.96873100	-1.11151000
S	0.02485500	0.00024900	0.18184600
O	-1.17518200	0.53326200	0.83534400
O	-0.19619700	-1.36527100	-0.36152900
O	1.26256100	0.11842200	0.95624800
O	-1.87180300	-1.22415300	-3.70443100
H	-1.87095500	-0.27399500	-3.48835300

H	-0.96372200	-1.54227100	-3.52053700
O	-3.89933900	0.01377900	1.91463100
H	-3.35120600	-0.80009000	1.83968100
H	-3.99338600	0.30544500	0.98764500
O	2.93188400	0.65705800	3.08138000
H	3.32413300	0.82096200	2.20823900
H	1.97613200	0.70919700	2.90956800
O	-1.61294000	3.20287300	0.46526100
H	-0.70612000	3.20740300	0.91311500
H	-1.76527200	2.24124600	0.38048200
O	2.71839800	-2.40219100	-0.49757200
H	2.27031700	-1.64271000	-0.92696200
H	2.00653300	-3.04791300	-0.33125700
O	2.66758900	2.77508100	-0.19196300
H	3.30062200	3.45481900	0.08176100
H	3.16356200	1.92234100	-0.10661900
O	4.65391500	-0.92010100	-1.93198400
H	3.81232100	-0.52726200	-2.23659600
H	4.33627600	-1.75382400	-1.54620000
O	-2.80157200	-1.83136200	-1.23495600
H	-1.99785000	-1.64978400	-0.71015200
H	-2.50252200	-1.67412000	-2.16564300
O	0.67836500	3.03945900	1.66161600
H	0.56533400	2.26490500	2.24869000
H	1.38682600	2.80104700	1.02478300
O	0.01010400	1.02077900	3.46763400
H	-0.92662800	1.32712500	3.30710500
H	0.18877300	1.25839100	4.38911200
O	-4.04398700	0.52846300	-0.84827200
H	-4.96200500	0.46718700	-1.14961600
H	-3.67790600	-0.38509300	-0.97046200
O	0.51427700	-2.45720700	-2.85117200
H	1.26503000	-1.83713600	-2.86800800
H	0.17983400	-2.30527200	-1.94936100
O	-2.13396600	1.40745200	-2.54824300
H	-1.41290200	1.12175500	-1.95516700
H	-2.94158200	1.20809400	-2.01527500
O	-2.19146700	-4.34902600	-0.11120300
H	-1.22787700	-4.26247000	0.01766300
H	-2.41941600	-3.62093800	-0.71771500
O	-2.38172900	-2.24984400	1.79759700
H	-2.36304500	-3.01389500	1.19259700
H	-1.44686700	-2.09958800	2.06225500
O	3.87406200	0.42505900	0.34293400
H	4.30671600	-0.02500200	-0.41536200
H	2.97344700	0.04700600	0.35710500
O	-1.22513100	3.76580600	-1.93134300
H	-1.65044900	2.94464900	-2.33067700
H	-1.38543700	3.61302500	-0.90272800
O	0.05349800	-1.71577600	2.84893000

H	0.97537800	-1.88274900	2.55386000
H	0.03865200	-0.75369500	3.03032600
O	0.49736500	-3.78729700	0.57578900
H	0.21872700	-2.84991200	0.56349500
H	0.70418200	-3.96612400	1.50483800
O	1.22143300	3.46437900	-2.38944300
H	-0.19473500	3.66216600	-2.09646400
H	1.74145800	3.28585100	-1.56835200
O	2.72489100	-2.07997900	2.27339400
H	2.77404700	-2.07372200	1.29567400
H	2.88571500	-1.15277500	2.53319700
O	-2.44425900	1.96899000	3.05151600
H	-2.28723200	2.58013100	2.31426600
H	-2.97082100	1.23675800	2.63674500

TS2-R9a[22]

C	1.16853500	0.52727600	-2.46427400
H	0.32635600	0.15833100	-3.08807100
H	1.14224600	2.66356600	-2.85827700
O	2.11348600	-0.14416500	-2.09928000
O	0.10252500	1.04880600	-1.07303800
S	-0.00350000	0.08132900	0.13431700
O	-1.21188500	0.46689500	0.89155800
O	-0.17229900	-1.30317200	-0.43367600
O	1.24802800	0.17405600	0.91199600
O	-1.92440500	-1.26559200	-3.77205300
H	-1.89556600	-0.31940400	-3.53638800
H	-1.02423700	-1.61080100	-3.59858300
O	-3.92747600	-0.04444100	1.96258000
H	-3.35723000	-0.84500900	1.89999100
H	-3.99028600	0.25542100	1.03572300
O	2.92346300	0.76923200	3.02821700
H	3.35890900	0.89827700	2.16907600
H	1.97856400	0.78019800	2.79759800
O	-1.71793100	3.11145500	0.46679600
H	-0.80206500	3.17241700	0.88938700
H	-1.82069000	2.13844200	0.41427000
O	2.66544700	-2.43910500	-0.48303800
H	2.18422000	-1.69750400	-0.89076300
H	1.96515100	-3.07700200	-0.24237400
O	2.62043800	2.81087200	-0.24326900
H	3.24846000	3.49876100	0.02107200
H	3.12747600	1.96545600	-0.16782600
O	4.75622400	-1.12991000	-1.74289300
H	4.07288300	-0.68657700	-2.27064900
H	4.25082000	-1.86652700	-1.35383500
O	-2.75035600	-1.83964300	-1.24449300
H	-1.90734500	-1.64178300	-0.78523700
H	-2.51749400	-1.70379200	-2.19604200
O	0.61116800	3.06934200	1.60771300

H	0.50849800	2.30524200	2.21193900
H	1.31574300	2.81999300	0.97176900
O	-0.00233300	1.08734400	3.45284300
H	-0.95236900	1.35805200	3.31709800
H	0.19750300	1.34161300	4.36528200
O	-3.98726100	0.50658000	-0.80508200
H	-4.90320800	0.44234300	-1.11217800
H	-3.61817100	-0.40580500	-0.93088100
O	0.41893000	-2.46912500	-2.85752900
H	1.27995600	-2.06899700	-3.04993300
H	0.20165800	-2.08662800	-1.98421600
O	-2.11231500	1.32980900	-2.60188100
H	-1.36104800	1.09670600	-2.01136100
H	-2.89770100	1.12949400	-2.03928500
O	-2.26406600	-4.33459100	-0.07692600
H	-1.29822800	-4.25014700	0.03935300
H	-2.49190900	-3.59135000	-0.66741500
O	-2.35783100	-2.26798700	1.87460700
H	-2.34702400	-3.01207100	1.24501400
H	-1.41647600	-2.08135500	2.08691900
O	3.84652200	0.47183400	0.31516200
H	4.29592800	-0.06818200	-0.37084500
H	2.93634800	0.11373600	0.35389600
O	-1.36460800	3.71263700	-1.92482800
H	-1.73873300	2.87647600	-2.34970900
H	-1.52318400	3.52721800	-0.89937100
O	0.09760000	-1.65887700	2.85122800
H	1.01341900	-1.80258200	2.53157700
H	0.05194300	-0.69034700	2.98492300
O	0.40228500	-3.70473600	0.58680200
H	0.13639000	-2.76663300	0.46633700
H	0.47190700	-3.81250400	1.54671300
O	1.09547600	3.51362600	-2.38032300
H	-0.33166900	3.65555100	-2.08189100
H	1.62681100	3.30894100	-1.57266500
O	2.79032000	-1.99228300	2.27518600
H	2.82505000	-2.00604600	1.29855200
H	2.92772100	-1.05698600	2.51618900
O	-2.50243500	1.95050200	3.07905200
H	-2.36322200	2.54396700	2.32358500
H	-3.01500600	1.19800700	2.68466000

P6

C	-1.17421700	0.98656200	-3.12970800
H	-0.07163400	0.79254700	-3.02574000
H	-1.88444900	-1.37370700	-3.24961900
O	-1.78758800	1.91970000	-2.74340200
O	-1.01281100	-0.73546300	-1.34342700
S	-0.46033200	-0.24329000	-0.04208100
O	0.78514900	-0.99871400	0.29615000

O	-0.16738900	1.23405700	-0.13788800
O	-1.49573200	-0.43426800	1.01644500
O	2.93928300	2.53623700	-3.00398400
H	2.66593400	1.63839100	-3.25328900
H	2.10130500	2.94276100	-2.71701900
O	2.99474600	-0.61608700	1.88008000
H	3.11384400	0.29138700	2.20389500
H	2.17155000	-0.55534500	1.34926500
O	-2.90513900	-1.40592500	3.06796000
H	-3.49117900	-1.36196900	2.29709100
H	-2.03057300	-1.49866600	2.65092900
O	2.68092300	-2.35762700	-1.04945600
H	2.04452600	-1.75226700	-0.61348800
H	3.56345600	-1.92782800	-0.88710100
O	-2.65249400	2.42932900	0.25590100
H	-1.84983400	1.98819800	-0.08756700
H	-2.26239100	3.11296500	0.82408800
O	-3.13872300	-2.85022100	-1.03156500
H	-2.29627700	-3.11081300	-0.45734300
H	-3.53984500	-1.98561800	-0.58098600
O	-4.42996200	1.30023700	-1.39810000
H	-3.81833700	1.31121900	-2.14693400
H	-4.00157300	1.92820600	-0.78211700
O	3.01110100	1.15305300	-0.65100600
H	2.69304700	1.49627500	0.22426700
H	3.16579600	1.89658300	-1.26799300
O	-1.07755200	-3.46400000	0.20786400
H	-0.79819900	-2.95623700	0.99936400
H	-0.40341600	-3.31652100	-0.48717400
O	-0.04174600	-2.41869400	2.57727900
H	0.83368700	-2.70962600	2.19703000
H	-0.18558700	-3.00434400	3.33015500
O	4.75343900	-0.91547200	-0.27006800
H	4.40740100	-0.94076900	0.64056900
H	4.34817700	-0.07262400	-0.56003600
O	0.64255600	3.23036400	-1.61847100
H	-0.25415600	3.36347700	-1.95658500
H	0.54669700	2.39046600	-1.11005500
O	1.72600700	-0.04719100	-2.63408200
H	2.02893400	0.31130200	-1.76420300
H	2.08584500	-0.94534300	-2.62951400
O	1.51881000	4.46777700	0.84050600
H	0.69012600	4.34133000	1.33297800
H	1.27444800	4.22501700	-0.07028500
O	2.31139900	2.01333200	1.77255600
H	2.11304600	2.95728800	1.58720300
H	1.51624600	1.61350300	2.18979200
O	-3.91857900	-0.82541300	0.16532700
H	-4.23841500	-0.08662800	-0.41303300
H	-3.00747000	-0.54661100	0.44580300

O	0.28976100	-2.92666300	-2.14466400
H	0.07114600	-1.99341700	-1.92710000
H	1.25436800	-2.99620100	-2.02655500
O	0.36153300	0.48321400	2.94859500
H	-0.58852600	0.69811700	2.93382000
H	0.36792000	-0.47096600	2.75451000
O	-0.59156100	3.12059900	1.92673300
H	-0.30003800	2.35434700	1.39164800
H	-1.02444800	2.71396000	2.69599900
O	-2.10019400	-2.31663500	-3.34344800
H	-1.23095100	-2.74471000	-3.22029400
H	-2.78379000	-2.61679000	-1.95306300
O	-2.40240200	1.34140500	3.00993300
H	-2.58042200	1.39451800	2.05517300
H	-2.65415600	0.41957000	3.22590800
O	2.33122400	-3.23445400	1.69980200
H	2.44025200	-3.19944800	0.73174200
H	2.80839900	-2.44072700	2.00208500

References

(1) Sander, R. Compilation of Henry's Law Constants (Version 4.0) for Water as Solvent. *Atmospheric Chemistry and Physics* **2015**, *15* (8), 4399–4981. https://doi.org/10.5194/acp-15-4399-2015.

(2) Onel, L.; Brennan, A.; Seakins, P. W.; Whalley, L.; Heard, D. E. A New Method for Atmospheric Detection of the CH_3O_2 Radical. *Atmospheric Measurement Techniques* **2017**, *10* (10), 3985–4000. https://doi.org/10.5194/amt-10-3985-2017.

(3) Khan, M. A. H.; Cooke, M. C.; Utembe, S. R.; Archibald, A. T.; Derwent, R. G.; Jenkin, M. E.; Morris, W. C.; South, N.; Hansen, J. C.; Francisco, J. S.; Percival, C. J.; Shallcross, D. E. Global Analysis of Peroxy Radicals and Peroxy Radical-Water Complexation Using the STOCHEM-CRI Global Chemistry and Transport Model. *Atmospheric Environment* **2015**, *106*, 278–287. https://doi.org/https://doi.org/10.1016/j.atmosenv.2015.02.020.

(4) Thornton, J. A.; Jaeglé, L.; McNeill, V. F. Assessing Known Pathways for HO2 Loss in Aqueous Atmospheric Aerosols: Regional and Global Impacts on Tropospheric Oxidants. *Journal of Geophysical Research: Atmospheres* **2008**, *113* (D5). https://doi.org/https://doi.org/10.1029/2007JD009236.

(5) Sander, R. Compilation of Henry's Law Constants (Version 5.0.0) for Water as Solvent. *Atmospheric Chemistry and Physics* **2023**, *23* (19), 10901–12440. https://doi.org/10.5194/acp-23-10901-2023.

(6) Gerritz, L.; Schervish, M.; Lakey, P. S. J.; Oeij, T.; Wei, J.; Nizkorodov, S. A.; Shiraiwa, M. Photoenhanced Radical Formation in Aqueous Mixtures of Levoglucosan and Benzoquinone: Implications to Photochemical Aging of Biomass-Burning Organic Aerosols. *J. Phys. Chem. A* **2023**, *127* (24), 5209–5221. https://doi.org/10.1021/acs.jpca.3c01794.

(7) Ranguelova, K.; Rice, A. B.; Khajo, A.; Triquigneaux, M.; Garantziotis, S.; Magliozzo, R. S.; Mason, R. P. Formation of Reactive Sulfite-Derived Free Radicals by the Activation of Human Neutrophils: An ESR Study. *Free Radical Biology and Medicine* **2012**, *52* (8), 1264–1271. https://doi.org/https://doi.org/10.1016/j.freeradbiomed.2012.01.016.

(8) Zamora, P. L.; Villamena, F. A. Theoretical and Experimental Studies of the Spin Trapping of Inorganic Radicals by 5,5-Dimethyl-1-Pyrroline N-Oxide (DMPO). 3. Sulfur Dioxide, Sulfite, and Sulfate Radical Anions. *J Phys Chem A* **2012**, *116* (26), 7210–7218. https://doi.org/10.1021/jp3039169.

(9) Marchaj, A.; Kelley, D. G.; Bakac, A.; Espenson, J. H. Kinetics of the Reactions between Alkyl Radicals and Molecular Oxygen in Aqueous Solution. *Journal of Physical Chemistry; (United States)* **1991**, *95:11*. https://doi.org/10.1021/j100164a051.

(10) Skodje, R. T.; Truhlar, D. G. Parabolic Tunneling Calculations. *J. Phys. Chem.* **1981**, *85* (6), 624–628. https://doi.org/10.1021/j150606a003.

(11) Smoluchowski, M. Versuch Einer Mathematischen Theorie Der Koagulationskinetik Kolloider Lösungen. *Zeitschrift für Physikalische Chemie* **1918**, *92U*, 129–168.

(12) Collins, F. C.; Kimball, G. E. Diffusion-Controlled Reaction Rates. *Journal of Colloid Science* **1949**, *4* (4), 425–437. https://doi.org/https://doi.org/10.1016/0095-8522(49)90023-9.
